# Site-specific DNA insertion into the human genome with engineered recombinases

**DOI:** 10.1038/s41587-025-02895-3

**Published:** 2025-11-06

**Authors:** Alison Fanton, Liam J. Bartie, Juliana Q. Martins, Vincent Q. Tran, Laine Goudy, Courtney Kernick, Matthew G. Durrant, Jingyi Wei, Zev Armour-Garb, April Pawluk, Silvana Konermann, Alexander Marson, Luke A. Gilbert, Theodore L. Roth, Patrick D. Hsu

**Affiliations:** 1 Arc Institute, Palo Alto, CA USA; 2 Department of Bioengineering, University of California, Berkeley, Berkeley, CA USA; 3 University of California, Berkeley − University of California, San Francisco, Graduate Program in Bioengineering, Berkeley, CA USA; 4 Department of Molecular and Cell Biology, University of California, Berkeley, Berkeley, CA USA; 5 Department of Chemistry, University of California, Berkeley, Berkeley, CA USA; 6 Gladstone-UCSF Institute of Genomic Immunology, San Francisco, CA USA; 7 Biomedical Sciences Graduate Program, University of California, San Francisco, San Francisco, CA USA; 8 Department of Pathology, Stanford University, Stanford, CA USA; 9 Department of Bioengineering, Stanford University, Stanford, CA USA; 10 Department of Biochemistry, Stanford University, Stanford, CA USA; 11 Helen Diller Family Comprehensive Cancer Center, University of California, San Francisco, San Francisco, CA USA; 12 Department of Urology, University of California, San Francisco, San Francisco, CA USA; 13 Center for Computational Biology, University of California, Berkeley, Berkeley, CA USA

**Keywords:** Gene therapy, Protein design, DNA recombination

## Abstract

Insertions of large DNA sequences into the genome are broadly enabling for research and therapeutic applications. Large serine recombinases (LSRs) can mediate direct, site-specific genomic integration of multi-kilobase DNA sequences without a pre-installed landing pad, albeit with low insertion rates and high off-target activity. Here we present an engineering roadmap for jointly optimizing their DNA recombination efficiency and specificity. We combine directed evolution, structural analysis and computational models to rapidly identify additive mutational combinations. We further enhance performance through donor DNA optimization and dCas9 fusions, enabling simultaneous target and donor recruitment. Our top engineered LSR variants, superDn29−dCas9, goldDn29−dCas9 and hifiDn29−dCas9, achieve up to 53% integration efficiency and 97% genome-wide specificity at an endogenous human locus and effectively integrate large DNA cargoes up to 12 kb for stable expression in non-dividing cells, stem cells and primary human T cells. Rational engineering of DNA recombinases enables precise and efficient single-step genome insertion for diverse applications across gene and cell therapies.

## Abstract (web summary)

Engineered DNA recombinases efficiently and specifically insert genetic cargos without the use of landing pads.

## Main

The ability to insert multi-kilobase DNA sequences efficiently and precisely into specified sites in the human genome in a single-step mechanism would advance both synthetic biology and gene therapy, enabling integration of gene circuits, large-scale pooled libraries and entire gene replacement rather than individual correction of diverse patient mutations^1^. However, previous approaches have been hampered by semi-random integration^2^, limited efficiency^3–6^, ceilings on donor template size^7^ or complex multi-component delivery^8–11^.

DNA recombinases are an emerging class of genome editing systems with important mechanistic advantages for achieving precise DNA insertions into the genome. The LSR enzyme family offers high recombination efficiency and site-specificity, operates independently from host DNA repair machinery and requires only two components: the recombinase and the donor DNA^12^. Natively, these enzymes facilitate mobile genetic element integration into bacterial genomes by recombining two double-stranded DNA attachment sites (attP and attB). During recombination, serine recombinases simultaneously cleave all four DNA strands, creating a covalent protein−DNA intermediate, followed by controlled strand exchange via subunit rotation and rejoining of the DNA ends^13^. They are readily adaptable to human cell engineering, integrating at either pre-installed landing pads or endogenous pseudosites (termed attH for the human genome) that closely resemble their native integration sequence^12,14,15^.

The broad application of LSRs is currently constrained by the prerequisite of DNA recognition sequences within the target genome. Pre-installing landing pads can be particularly challenging in primary cells or in vivo settings. Even when suitable pseudosites exist, ensuring high recombinase specificity remains difficult. Our previous screening of more than 60 diverse LSR orthologs in human cells revealed that many integrated into genomic pseudosites but often with low specificity, targeting hundreds of sites with varying efficiencies^12^. Attempts to engineer LSRs and other serine recombinase systems to target endogenous sequences through mutagenesis or DNA-binding-domain fusions have historically yielded inefficient or non-specific systems^3,16–20^. Engineering tyrosine recombinases to target endogenous sequences showed greater success, yet their inherent bidirectionality limits genome integration applications^21–23^. In the present study, we developed LSRs with high efficiency and high specificity for direct human genome integration without pre-engineered landing pads.

**Fig. 1:**
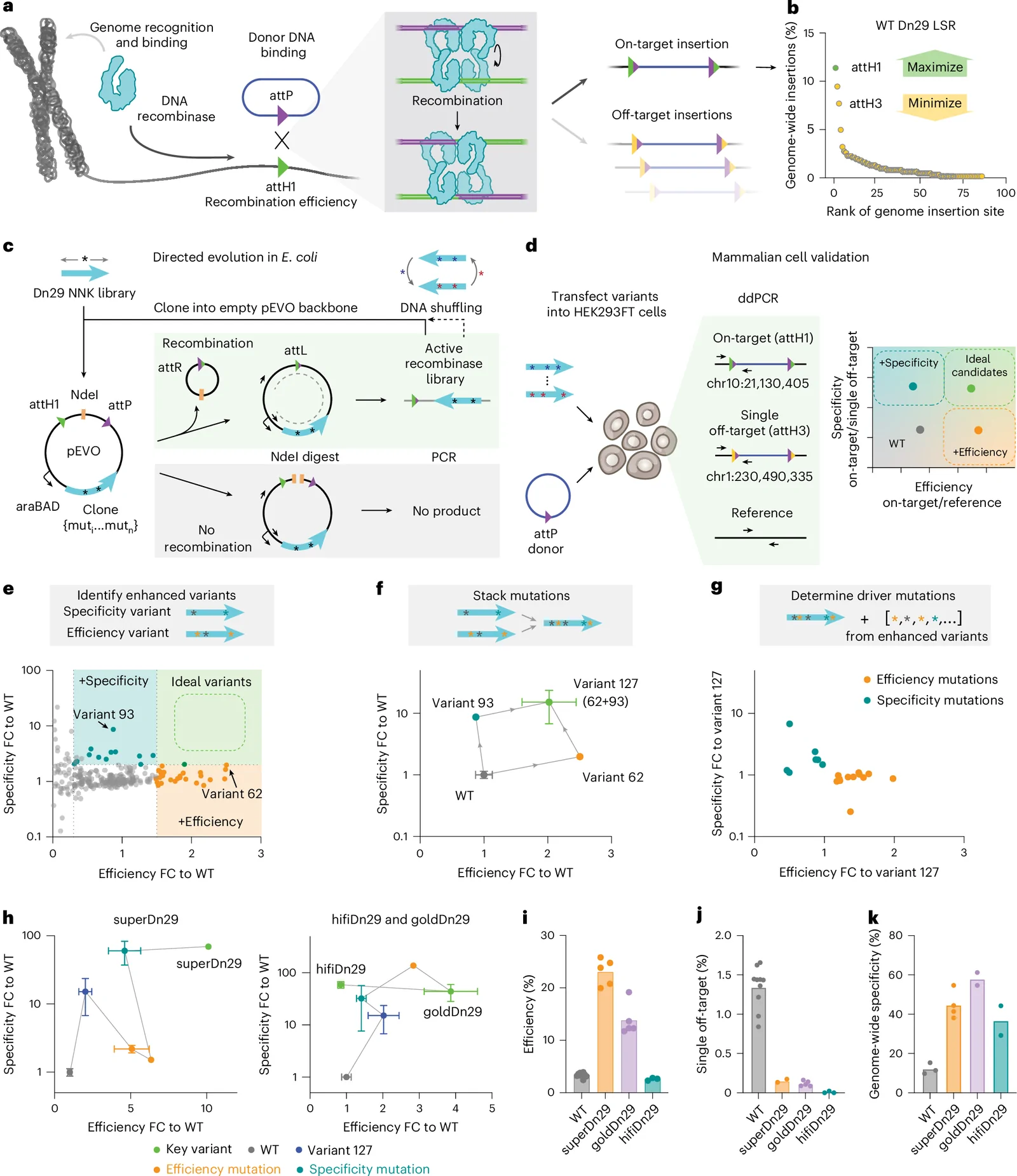
Directed evolution of LSRs with improved efficiency and specificity. **a**, Overview of engineering strategies to improve integrations into endogenous genomic sites. By improving genome recognition and binding, recombination efficiency and donor DNA binding, we aim to enhance recombination between a plasmid containing the recombinase attachment site attP and a genomic pseudosite, attH1, by maximizing on-target integrations and minimizing off-targets. **b**, Genome-wide specificity profile of WT Dn29. The green dot is the on-target site, attH1 (chr10:21,130,405). The yellow dots are off-target sites, ranked by their insertion efficiency. Data shown are the same as Fig. 2i. **c**, Schematic of Dn29 directed evolution scheme in *E. coli*. An evolution backbone, pEVO, expresses a library of Dn29 variants containing NNK codons across the coding sequence (CDS) and contains attP and attH1 sites. Active variants remove the NdeI site, allowing selective PCR recovery after digestion. The active recombinase library can be shuffled to generate higher-order combinations of beneficial mutations and re-cloned into pEVO for subsequent rounds of evolution. **d**, Schematic of mammalian cell validation of evolved LSR variants. Colonies from the active recombinase library are randomly selected and validated in HEK293FT cells. ddPCR at the on-target (attH1), a single-off target (attH3) and a genomic reference measures the efficiency (attH1/reference) and specificity (attH1/attH3). **e**, Efficiency and specificity of 247 LSR library members in HEK293FT cells, shown as fold change (FC) to WT. Colored dots represent enhanced variants with >2-fold WT specificity (teal) or >1.5-fold WT efficiency (orange). Each dot represents the mean of *n* = 2 biological replicates. **f**, Efficiency and specificity of WT Dn29 (blue, *n* = 16 biological replicates), variant 62 (orange, *n* = 2 biological replicates) and variant 93 (teal, *n* = 2 biological replicates) and variant 127 (green, *n* = 10 biological replicates), generated by stacking all mutations found on variants 62 and 93. Dots and error bars represent the mean ± s.d. of samples with *n* ≥ 3 biological replicates, shown as FC to WT. **g**, Efficiency and specificity of variants harboring driver mutations, shown as FC to variant 127. Variants are generated by adding individual mutations from the enhanced variants in **e** on top of variant 127. Dots represent the mean of *n* = 2 biological replicates. **h**, Efficiency and specificity of all variants generated across rounds of mutation stacking, following the path from WT Dn29 (gray, *n* = 16 biological replicates, same data as **f**) to superDn29, goldDn29 and hifiDn29 (green, *n* = 2, 5 and 3 biological replicates, respectively). Variant 127 is shown in blue (*n* = 12 biological replicates, same data as **f**); orange dots indicate the addition of efficiency mutations; and teal dots indicate the addition of specificity mutations, with each dot representing 2−7 biological replicates. Dots and error bars represent the mean ± s.d. of samples with *n* ≥ 3 biological replicates. Gray lines indicate the lineage of mutation addition between variants. **i**, On-target efficiency of WT (*n* = 10 biological replicates), superDn29 (*n* = 5 biological replicates), goldDn29 (*n* = 5 biological replicates) and hifiDn29 (*n* = 3 biological replicates). Data presented are the same as shown in **h**. **j**, Efficiency of integration into a single off-target (attH3) of WT (*n* = 10 biological replicates), superDn29 (*n* = 2 biological replicates), goldDn29 (*n* = 5 biological replicates) and hifiDn29 (*n* = 3 biological replicates). **k**, Genome-wide specificity of on-target integration compared to all genomic insertions of WT (*n* = 3 biological replicates), superDn29 (*n* = 4 biological replicates), goldDn29 (*n* = 2 biological replicates) and hifiDn29 (*n* = 2 biological replicates).

We reasoned that three key mechanistic limitations impeding efficient and specific endogenous integrations are genome recognition and binding, donor DNA binding and recombination efficiency (Fig. 1a). To address these challenges, we developed a framework to guide recombinase engineering, using the genome-targeting LSR Dn29 as a proof of concept. We combined four strategies—directed evolution, machine-learning-guided mutation stacking, dCas9 fusions and donor attachment site sequence optimization—to create recombinases that specifically integrate DNA cargo at a single endogenous locus in the human genome. Furthermore, we demonstrate integration in stem cells and primary T cells and show that the integration site maintains similar transgene expression to validated safe harbors, such as *AAVS1*, while reducing transcriptome perturbation.

## Results

### A framework for recombinase engineering to enable site-specific genome insertion

We previously reported that LSRs catalyze DNA integration directly into endogenous genomic pseudosites^12^, with the number and identity varying across LSR orthologs based on the sequence similarity between their native attachment site (attB) and the targeted genome (Fig. 1a). In the present study, we sought to establish engineering principles for optimizing any LSR for site-specific genome integration, choosing the genome-targeting LSR Dn29 as a proof of concept owing to its favorable specificity and efficiency profile^12^. Dn29 integrates into the endogenous genome at 5% overall efficiency and directs 12% of insertions into a top site (termed attH1, located within an intron of *NEBL*), with three prominent off-targets (attH2, attH3 and attH4) each comprising 5−10% of total insertions and approximately 80 other low-frequency off-target sites (Fig. 1b).

Our framework involves three key steps: increasing on-target integration efficiency at attH1, reducing insertion frequency at prominent off-targets and minimizing the long tail of low-frequency off-target insertions. To measure our progress toward these goals, we developed three key metrics: ‘efficiency’ as the percentage of attH1 sites that receive an insertion; ‘specificity’ as the ratio of insertions into attH1 versus the prominent off-target attH3; and ‘genome-wide specificity’ as the ratio of attH1 insertions relative to all on-target or off-target integration events (Fig. 1a, Supplementary Fig. 1a and Methods).

### Directed evolution of LSRs with improved efficiency and specificity

Because the protein−DNA recognition code of LSR enzymes for their DNA target site is unknown but unlikely to be modular like zinc finger or TALE proteins^24^, we reasoned that Dn29 would need modification via directed evolution to improve on-target integration into attH1. To increase Dn29 insertion efficiency at attH1 and disfavor integration into off-targets, we performed deep scanning mutagenesis of Dn29 at single-site saturation and tested the variant library in an intra-plasmid recombination reporter containing the attH1 and attP sites (Fig. 1c). Successful recombinants are positively selected by removing an intervening restriction enzyme site, whereas unproductive variants are eliminated via plasmid digestion^23,25^. To increase library complexity beyond single mutants, which may not be sufficient for modifying LSR target site preference, we performed DNA fragmentation and reassembly to shuffle successful mutations (Supplementary Fig. 1b)^26^.

**Extended Data Fig. 1:**
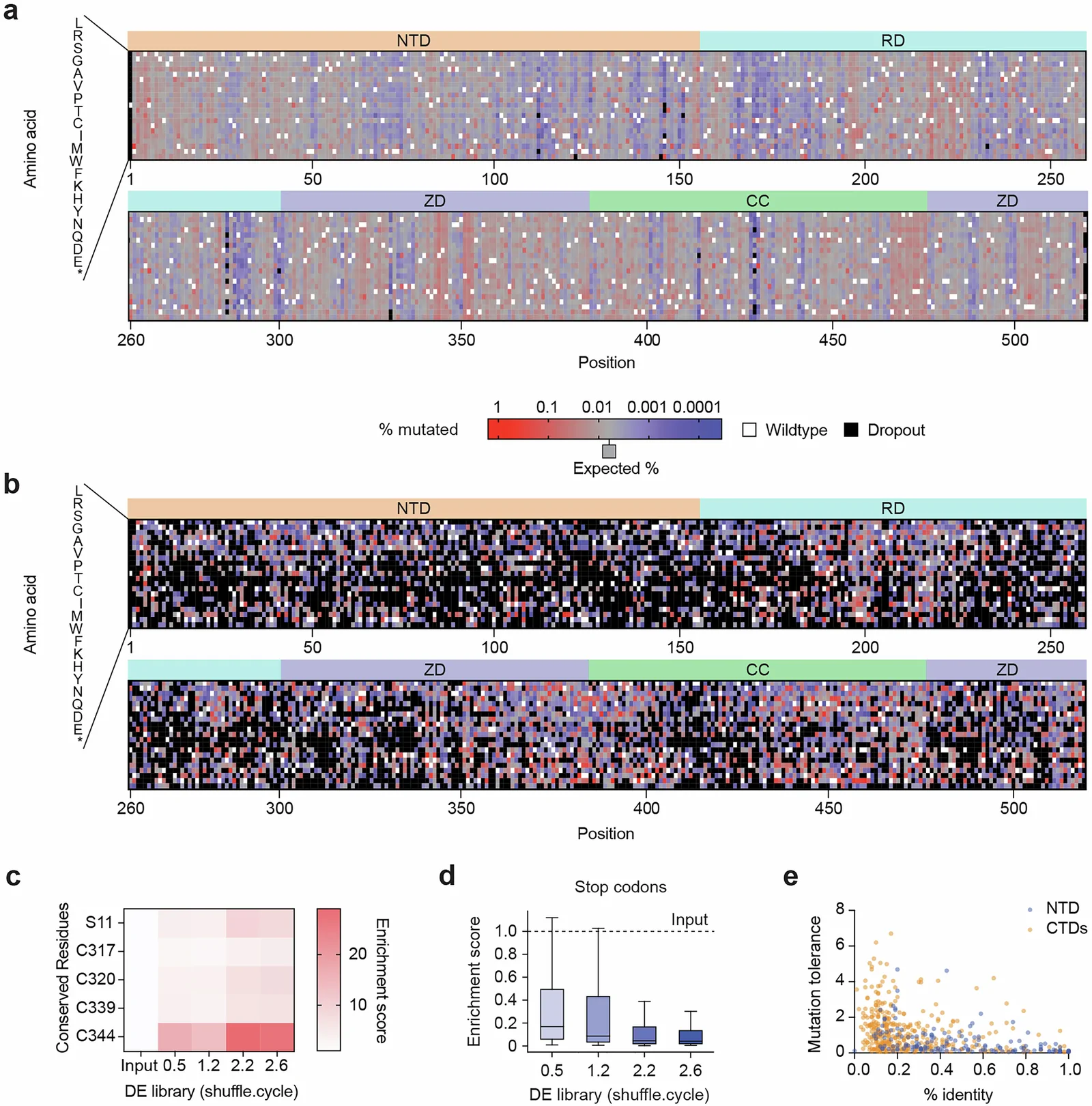
Quality control of input and output libraries. **a**,**b**, Mutational distribution of (**a**) input and (**b**) output libraries. White indicates the WT amino acid, black indicates an amino acid dropout, and red and blue indicate enrichment and depletion, respectively, compared to the expected mutation frequency (0.006%, shown in gray). Each value is normalized by the number of encoding codons in the NNK library. NTD: N-terminal domain; RD: recombinase domain; ZD: zinc-ribbon domain; CC: coiled-coil motif. **c**, Enrichment score of the catalytic serine (S11) and four conserved zinc-coordinating cysteines throughout various timepoints in the directed evolution campaign. **d**, Box plot of stop codon enrichment scores across all coding sequence positions at various time points (*n* = 515). Boxes: interquartile range (IQR); line: median; whiskers: values within 1.5 times IQR. **e**, Correlation between mutational tolerance (average non-WT residue enrichment) and phylogenetic conservation (% identity from multiple sequence alignment of 106 LSR clusters within 30% identity of Dn29). Each dot represents a position in the CDS, colored by domain. NTD: N-terminal domain, CTDs: C-terminal domains. Pearson *r* = -0.3577, two-tailed *P* = 5.432e-17.

After 12 evolution rounds and two DNA shuffles, we sequenced the output library with short-read and long-read sequencing (Supplementary Fig. 1c−f). The resulting library contained a median of three amino acid changes per variant, with 72% of the pool carrying 2−6 mutations (Supplementary Fig. 1g). Additionally, the evolution process exhibited robust selection against non-functional variants, with mutation dropout rates increasing from 0.0035% in the input library to 44.9% in the output library (Extended Data Fig. 1a,b). As expected, we saw strong retention of the catalytic serine and conserved zinc-coordinating cysteines^27^ and depletion of stop codons (Extended Data Fig. 1c,d), and we observed that phylogenetic conservation negatively correlated with mutational tolerance (Pearsonʼs *r* = −0.3577, two-tailed *P* < 0.0001) (Extended Data Fig. 1e). Compared to the input library, the output library was enriched for mutations in several hotspots, particularly in the C-terminal region (Supplementary Figs. 1e and 2). We observed higher mutational sensitivity in the N-terminal domain (NTD), consistent with its important functional roles in inter-subunit interactions, subunit rotation, catalysis and ligation^28^.

**Extended Data Fig. 2:**
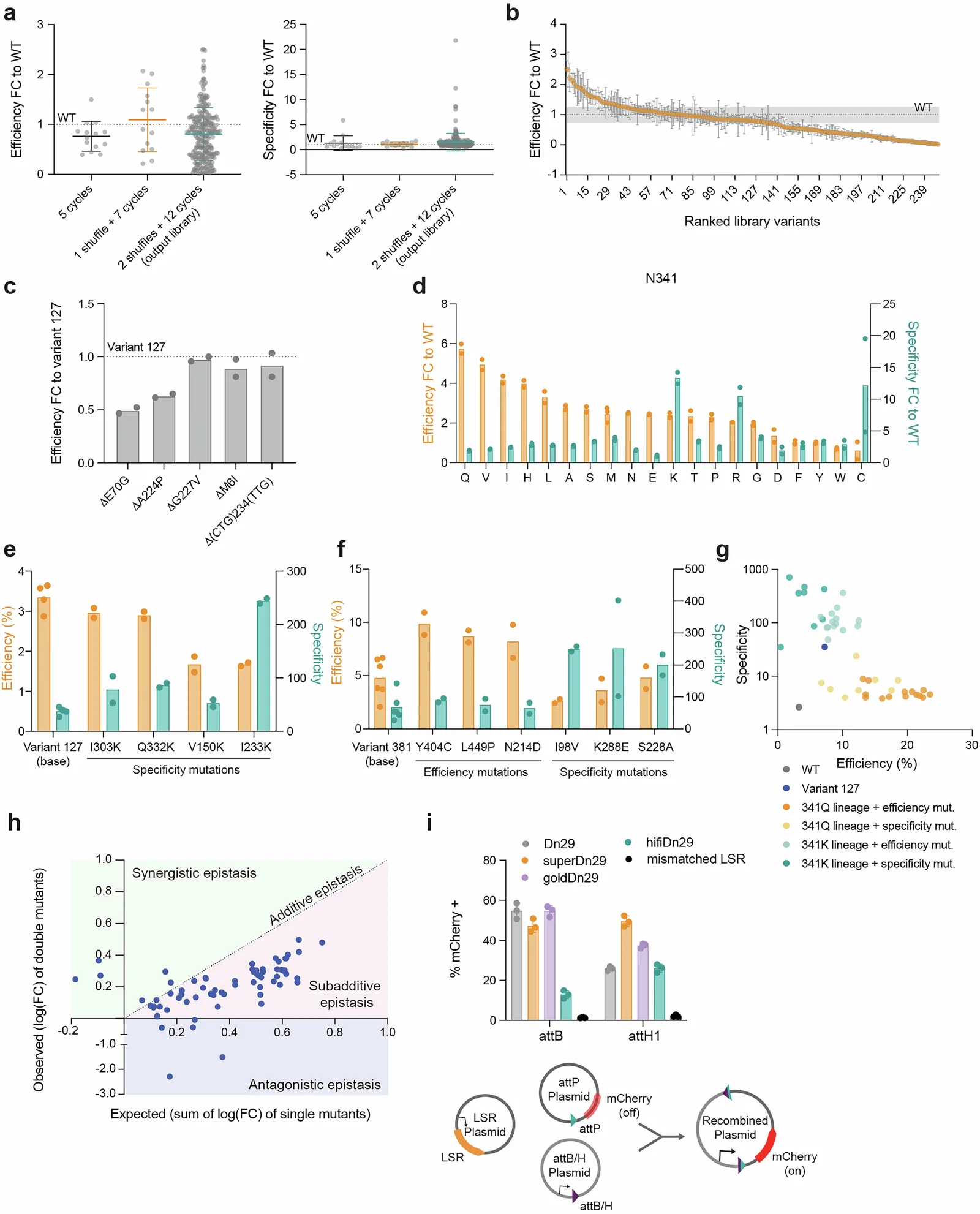
Exploration of mutational landscape with combinatorial mutations. **a**, Integration efficiency (left) and specificity (right) of variants from different libraries throughout directed evolution, as fold change to WT Dn29. Each dot represents the mean of *n* = 2 biological replicates. Dotted line represents the average WT activity. **b**, Integration efficiency of output library variants, as fold change to WT Dn29. Dots and error bars represent the mean ± s.d. of *n* = 2 biological replicates. The dotted line represents average WT activity, and gray bands represent the s.d. of *n* = 36 biological replicates of WT Dn29. **c**, Integration efficiency of mutations in variant 127 reverted to WT, shown as fold change to variant 127. Bars and error bars represent the mean ± s.d. of *n* = 2 biological replicates, shown as dots. **d**, Integration efficiency (orange, left *y* axis) and specificity (teal, right *y* axis) of variant 127 with position 341 saturation mutagenesis, shown as fold change to WT. *n* = 2 biological replicates. **e**, Integration efficiency (orange, left *y* axis) and specificity (teal, right *y* axis) of variant 127 with lysine mutations of putative DNA binding residues. *n* = 2 biological replicates. **f**, Integration efficiency (orange, left *y* axis) and specificity (orange, right *y* axis) of variant 381 with significant (one-tailed *P* < 0.05) mutations from second validation round. *n* = 6 (variant 381), *n* = 2 (other variants) biological replicates. **g**, Integration efficiency vs. specificity of 341K and 341Q lineages with driver mutations. *n* = 2 biological replicates. **h**, Epistatic interactions between rounds 2 and 3 mutations. *x* axis: expected effect (sum of single mutant log_2_(fold change)); *y* axis: observed effect (double mutant log_2_(fold change)). Dots represent *n* = 2 biological replicates, the dotted line is the identity line. Pearson *r* = 0.2841. **i**, Top, Recombination efficiencies of Dn29 variants using the three plasmid recombination assay, shown as percent of mCherry^+^ cells. Bars and error bars represent the mean ± s.d. of *n* = 3 biological replicates, shown as dots. Bottom, schematic of plasmid recombination assay for attachment site recombination. mCherry expresses upon recombination between attP and attB/H.

Next, we functionally evaluated variant library members in human cells from three directed evolution timepoints: after five cycles, after seven cycles plus one shuffle and after 12 cycles plus two shuffles (the final output library). We assessed the efficiency (insertion into attH1) and specificity (attH1/attH3 ratio) of each variant (Fig. 1d), observing a shift toward higher on-target efficiency between the first and second timepoints and more variants with improved specificity after the third (Extended Data Fig. 2a,b). Although our selection was based solely on integration efficiency, the emergence of specificity-enhanced variants suggests that some mutations improved target discrimination without compromising recombination activity.

Because most variants exhibited either enhanced efficiency or specificity, but not both, we hypothesized that combining mutations across these two classes would achieve both desired properties (Fig. 1e). First, we combined the four mutations from variant 62 (2.5-fold wild-type (WT) efficiency) and variant 93 (8.6-fold WT specificity) into variant 127, which demonstrated 13.4-fold specificity and 1.8-fold efficiency (Fig. 1f).

The ability to simultaneously improve LSR efficiency and specificity motivated systematic exploration of all mutations in our efficiency-enhanced and specificity-enhanced variants. We sought to identify the causal point mutations and remove passenger mutations, thereby enabling higher-order mutation stacking. Individual validation of each mutation in variant 62 identified E70G and A224P as the driving efficiency mutations (Extended Data Fig. 2c). The sole amino acid mutation in variant 93, N341K, was further investigated by substituting N341 with every amino acid (Extended Data Fig. 2d). Many substitutions increased activity, with N341Q improving efficiency 2.3-fold and positively charged residues robustly improving specificity (N341K: 6.7-fold, N341R: 5.3-fold).

We next assessed all point mutations from variants with at least 1.5-fold WT efficiency (*n* = 47 mutations) and two-fold WT specificity (*n* = 28 mutations) (Fig. 1g). We also included lysine mutations at putative DNA-interfacing residues, chosen based on alignment with the crystal structure of *Listeria* integrase C-terminal domain bound to attP (Protein Data Bank (PDB): 4KIS)^29^, hypothesizing that positively charged mutations could modify DNA binding (Extended Data Fig. 2e). These mutations were individually installed into variant 127, identifying 12 additional efficiency and seven additional specificity driver mutations, each contributing 1.2-fold to 2.5-fold efficiency and 1.1-fold to 6.8-fold specificity improvements over variant 127 (Fig. 1g). A final round of mutation validation (Extended Data Fig. 2f) yielded a final list of 21 efficiency and 12 specificity driver mutations for further rational engineering (Supplementary Table 1).

We optimized Dn29 variants through sequential mutation layering, introducing single mutations into the best variant(s) from the previous round. We began with two lineages: an efficiency lineage containing 341Q and a specificity lineage containing 341K (Extended Data Fig. 2g). Double mutations showed mostly additive and subadditive effects, with rare antagonistic or synergistic epistasis (Extended Data Fig. 2h). This process yielded three key variants: superDn29 (10-fold efficiency and 70-fold specificity), goldDn29 (four-fold efficiency and 44-fold specificity) and hifiDn29 that combined goldDn29 with four additional specificity driver mutations to achieve WT efficiency with high specificity that approached the droplet digital polymerase chain reaction (ddPCR) limit of detection (Fig. 1h−j and Supplementary Table 2). These variants demonstrated a substrate preference shift toward attH1 while maintaining or reducing activity at the native attB attachment site, indicating partial substrate reprogramming rather than expansion (Extended Data Fig. 2i). Finally, we conducted whole-genome insertion profiling using an unbiased next-generation sequencing (NGS)-based integration site assay that quantifies the relative frequency of on-target versus off-target integrations (Methods). This analysis confirmed the substantial improvement in genome-wide specificity, which increased from 12% on-target integration with WT Dn29 to 40−60% with the engineered variants (Fig. 1k). Overall, we identified specific mutations driving efficiency or specificity improvements, enabling rational engineering of optimized recombinases.

### Computational modeling and structural analysis of recombinase mutation stacking

**Fig. 2:**
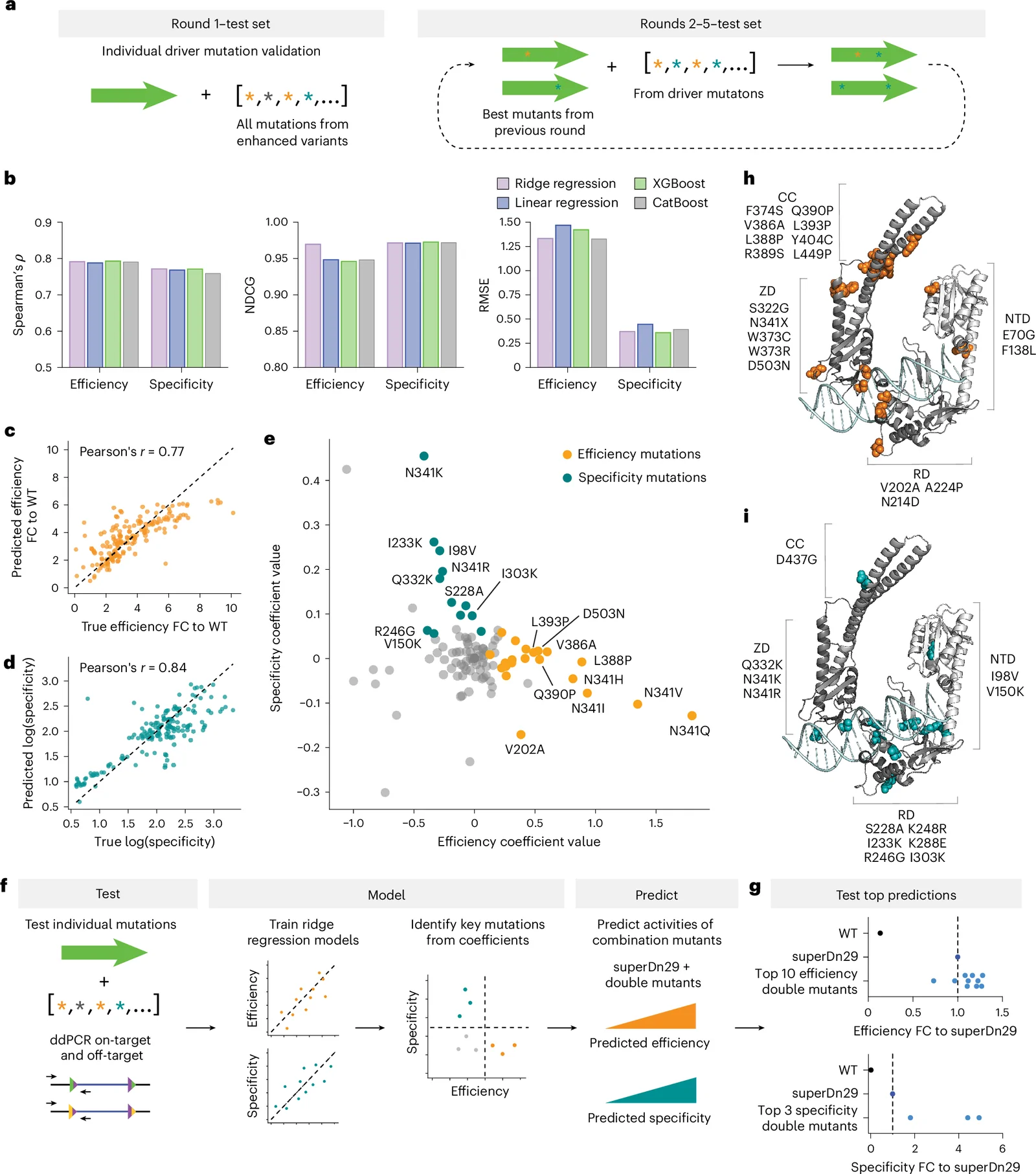
Computational modeling and structural analysis of recombinase mutation stacking. **a**, Division of data into training and test sets. Models are trained on the individual driver mutation validation data (round 1) and tested on the iterative rounds of higher-order mutants (rounds 2−5). **b**, Evaluation metrics (Spearman’s *ρ*, NDCG and RMSE) of linear regression, ridge regression, XGBoost and CatBoost models. **c**, Predicted versus true efficiency of higher-order mutants, as fold change (FC) to WT. Pearsonʼs *r* = 0.77, two-tailed *P* = 7.69 × 10^−34^. **d**, Predicted versus true specificity of higher-order mutants, as the log transformation of the ratio of attH1/attH3. Pearsonʼs *r* = 0.84, two tailed *P* = 1.18 × 10^−44^. **e**, Coefficient values of mutations in the efficiency and specificity models. Colored dots indicate mutations identified as efficiency (orange) or specificity (teal) driver mutations from Fig. 1. **f**, Schematic of the workflow for testing and modeling single mutations for the prediction of protein activities of higher-order mutants. **g**, Efficiency (top) and specificity (bottom) of top model-guided combinatorial mutants, generated by predicting the activity of combining two mutations on top of superDn29. Each dot represents the mean of *n* = 6 biological replicates for WT and superDn29 and *n* = 2 biological replicates for all model-guided variants. **h**,**i**, Efficiency (**h**) and specificity (**i**) mutations mapped to the AlphaFold3 structure of Dn29 bound to attB-R. CC, coiled-coil motif; RD, recombinase domain; ZD, zinc-ribbon domain.

We demonstrated that LSR-directed evolution with efficiency and specificity classification enables iterative mutation combinations to generate LSRs with desired functional profiles. To expedite experimental testing higher mutational loads, we developed a computational model for predicting combinatorial variant activity from single mutant data. The variants were divided into distinct rounds, with the individual mutation validation in round 1 (Fig. 1g) and the iterative mutation stacking experiments comprising rounds 2−5. We trained two linear models (linear regression and ridge regression) and two gradient boosting models (XGBoost and CatBoost)^30,31^ on one-hot encoded variant sequences from round 1 and then tested these models on rounds 2−5 (Fig. 2a).

We evaluated model performance using root mean squared error (RMSE), Spearman’s rank correlation and normalized discounted cumulative gain (NDCG). NDCG was prioritized as the key evaluation metric because it emphasizes higher-activity mutants, matching our experimental validation priorities. Although all models performed well, ridge regression excelled for both efficiency and specificity, showing high accuracy (NDCG = 0.970 for efficiency, 0.971 for specificity) in predicting higher-order mutant activities (Fig. 2b−d). The performance of linear models in extrapolating single mutant activity to higher-order mutants supports our previous observations that LSR mutations are largely additive (Extended Data Fig. 2h) and aligns with previous research showing that regularized linear models perform well across mutagenesis datasets of 14 different enzymes^32^.

**Extended Data Fig. 3:**
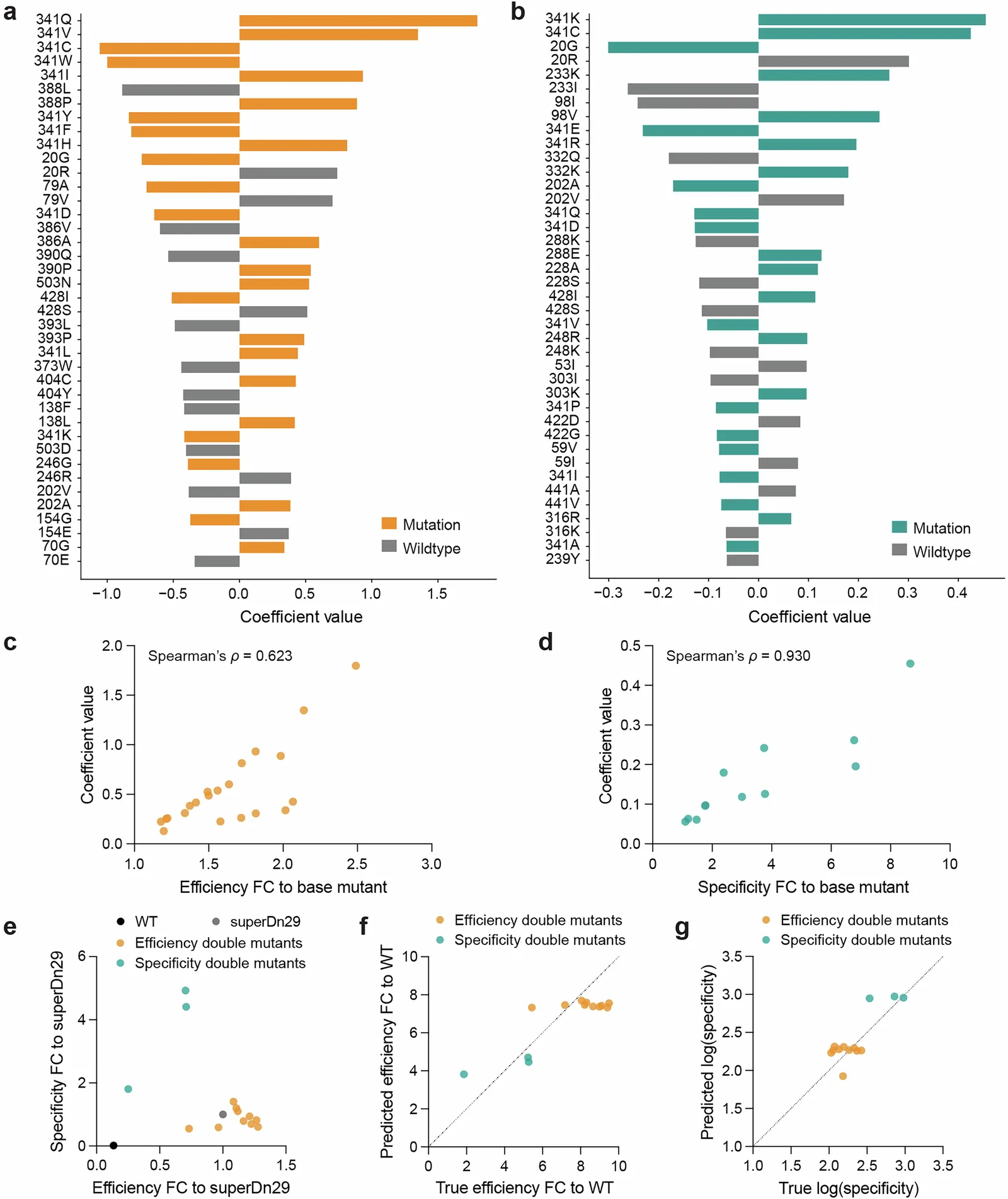
Model coefficients, predictions, and validation for recombinase engineering. **a**,**b**, Top 40 coefficients of the (**a**) efficiency model and (**b**) specificity model. **c**,**d**, Correlation between the (**c**) efficiency (Spearman’s *ρ* = 0.623, two-tailed *P* = 0.0025) and (**d**) specificity (Spearman’s *ρ* = 0.930, two-tailed *P* = 2.643e-04) models’ coefficient values and experimental impact of mutations, shown as fold change to the base mutant (variant 127 from mutations identified in the first round of individual validation (Fig. 1g, Extended Data Fig. 2C-E), or variant 381 from mutations identified in the second round of individual mutation validation (Extended Data Fig. 2F)). **e**, Efficiency and specificity of model-guided variants, which each contain two mutations on top of superDn29, and were designed to maximize efficiency (orange) or specificity (teal). Each dot represents the mean of *n* = 2 biological replicates. **f**,**g**, Comparison between the model-predicted and true (**f**) efficiency and (**g**) specificity of the model-guided variants. Each dot represents the mean of *n* = 2 biological replicates.

To identify key mutations, we examined regression coefficients, revealing strong concordance between computationally and experimentally identified impactful mutations (Fig. 2e and Extended Data Fig. 3a,b). The model coefficients strongly correlated with experimental fold changes (efficiency: *ρ* = 0.623, two-tailed *P* = 0.0025; specificity: *ρ* = 0.930, two-tailed *P* < 0.0001; Extended Data Fig. 3c,d), demonstrating that the interpretability of linear models enables the automated identification of impactful mutations.

We outline an approach for applying regression models to predict efficiency and specificity of higher-order mutants (Fig. 2f). From individual mutations measured for specificity and efficiency by ddPCR, we generated ridge regression models, examined their coefficients to quantify the impact of key mutations and predicted activity of higher-order mutants, enabling prioritization of mutants for experimental testing. To demonstrate this approach, we predicted the activities of all double mutants added to superDn29, skipping round 6 to directly test round 7 of iteration. We tested the top 10 efficiency and top three specificity variants, finding that eight of 10 efficiency variants and three of three specificity variants performed better than superDn29 (Fig. 2g and Extended Data Fig. 3e−g). Although the three key variants (superDn29, goldDn29 and hifiDn29) that we experimentally identified served as the primary variants for subsequent characterization studies, these model-guided variants demonstrate the potential for further optimization and represent valuable targets for future investigation. Overall, we demonstrate that model-guided recombinase design across multiple activity axes can further push efficiency and specificity beyond spaces easily reachable by traditional directed evolution.

**Extended Data Fig. 4:**
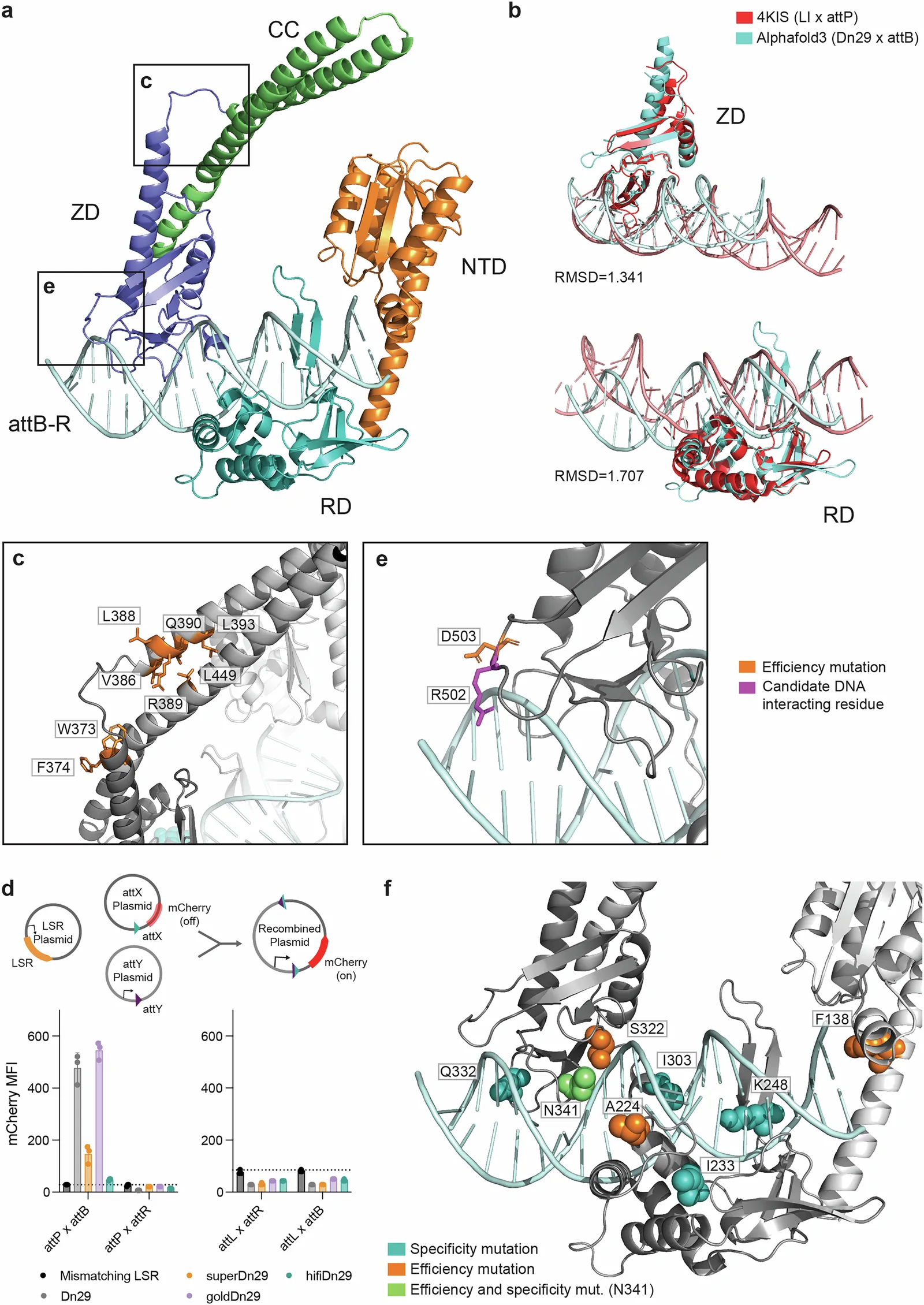
Mapping driver mutations on an AlphaFold3 model of Dn29 bound to attB. **a**, AlphaFold3 model of Dn29 bound to the attB-R half site, colored by protein domain. NTD: N-terminal domain, RD: recombinase domain, ZD: zinc-ribbon domain, CC: coiled-coil motif. **b**, Alignment of the Dn29 attB-R AlphaFold3 structure to listeria integrase (LI) C-terminal domain bound to attP crystal structure (PDB: 4KIS). Top: zinc-ribbon domain (ZD), bottom: recombinase domain (RD). Root mean square deviation (RMSD) values provided. **c**, Coiled-coil hinge region with efficiency mutations (orange). Corresponds to box **c** in panel **a**. **d**, Top, schematic of plasmid recombination assay for attachment site recombination. mCherry expresses upon recombination between attachment sites X and Y. Bottom, recombination of Dn29, key variants, and mismatching LSR control between attP, attB, attL, and attR, measured by mCherry median fluorescence intensity (MFI). Dotted line indicates the background fluorescence associated with the mismatching LSR control. Bars and error bars represent the mean ± s.d. of *n* = 3 biological replicates, shown as dots. **e**, Efficiency mutation D503N (orange) and neighboring DNA-interfacing residue R502 (magenta). Corresponds to box **e** in panel **a**. **f**, Specificity (teal) and efficiency (orange) mutations near DNA-interfacing residues. N341 (green) is both a specificity and efficiency mutation.

To gain mechanistic insights from our mutational landscape, we generated an AlphaFold3 structural model of Dn29 bound to the attB-R DNA half-site (Fig. 2h,i and Extended Data Fig. 4a)^33^, which showed high alignment (zinc-ribbon domain RMSD = 1.341, recombinase domain RMSD = 1.707) to the *Listeria* integrase C-terminal domain bound to attP (PDB: 4KIS)^29^ (Extended Data Fig. 4b). We identified an efficiency mutation hotspot (residues 373−393 and 449) in the coiled-coil motif hinge region, with four of the eight activating mutations converting to proline residues (L388P, Q390P, L393P and L449P), suggesting that destabilization of the helical secondary structure in this region enhances activity, potentially by modifying tetramer stabilization or autoinhibitory control (Extended Data Fig. 4c)^34^. Despite previous work on a different ortholog that identified mutations in this region as enabling the excision reaction^35^, our key variants maintained unidirectionality (Extended Data Fig. 4d). Another key efficiency mutation (D503N) reduces negative charge in a tri-aspartic acid stretch (503−505), likely enhancing DNA phosphate backbone interactions (Extended Data Fig. 4e).

Many specificity driver mutations localize near the DNA-binding interface, often replacing neutral amino acids with positively charged ones, potentially strengthening DNA interactions (Extended Data Fig. 4f). This combined computational and structural analysis provides a multifaceted understanding of how specific mutations impact LSR function, informing rational engineering to enhance LSR performance.

### Target and donor DNA recruitment with LSR−dCas9 fusions

**Fig. 3:**
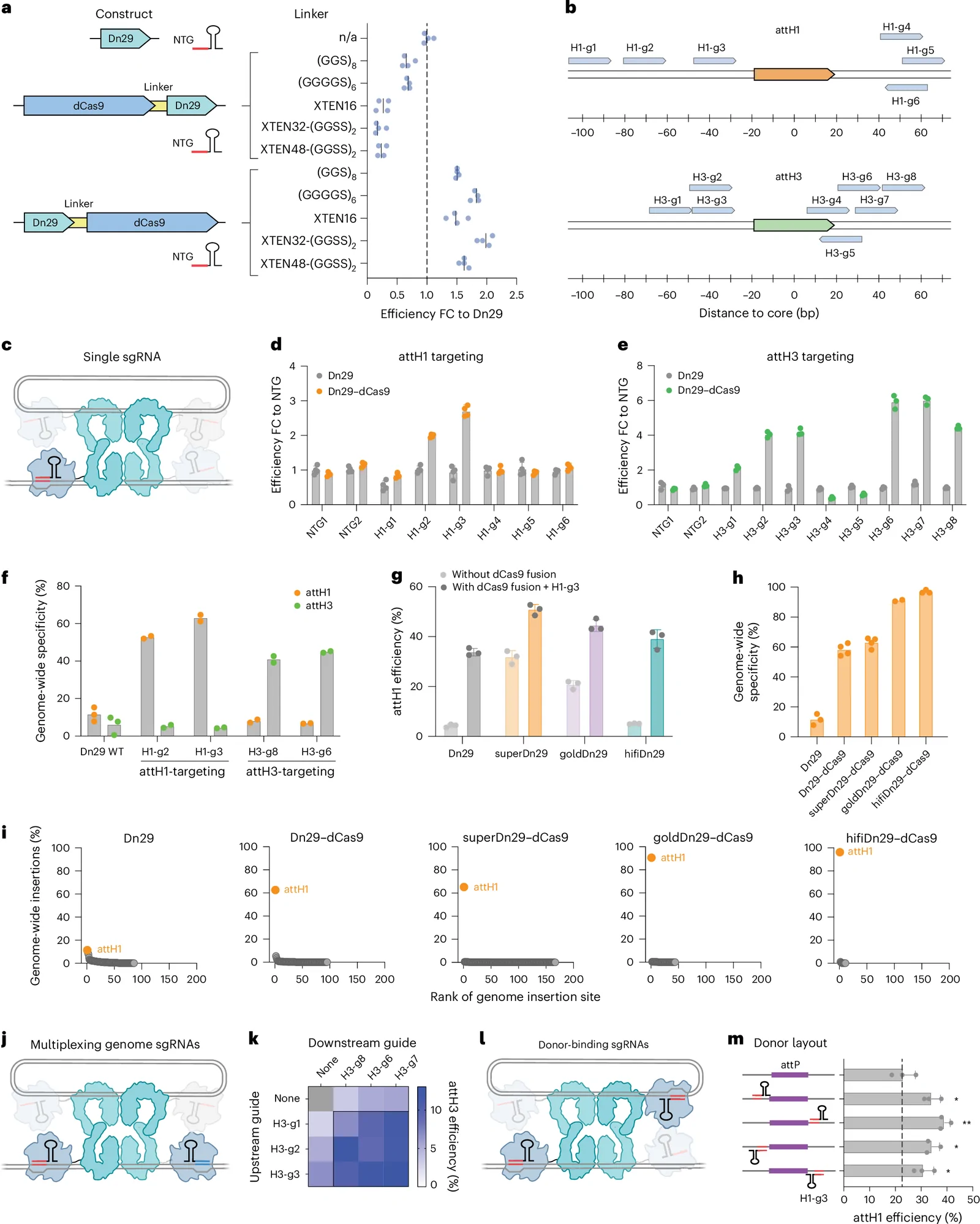
Target and donor DNA recruitment with LSR−dCas9 fusions. **a**, Schematic of LSR−dCas9 fusion orientations and recombination efficiency with various linkers at attH1 with an NTG. The lines represent the mean of *n* = 4 biological replicates, shown as dots. **b**, Schematic of sgRNA targets for the attH1 (chr10:21,130,405) and attH3 (chr1:230,490,334) pseudosites. **c**, Schematic of the LSR−dCas9 tetrameric complex, with a single sgRNA targeting the genome. **d**,**e**, Integration efficiencies of Dn29 and Dn29−dCas9 at attH1 (**d**) and attH3 (**e**) with sgRNAs targeting proximal to the respective pseudosites. Data are shown as fold change (FC) relative to NTG. Bars and error bars represent mean ± s.d. of *n* = 3 biological replicates, shown as dots. **f**, Biasing Dn29−dCas9 integrations to different pseudosites. Shown is the percent of all genome-wide insertions at attH1 (orange) and attH3 (green), using sgRNAs targeting the pseudosites. **g**, Integration efficiencies of Dn29 variants at attH1, with and without the dCas9 fusion and guide H1-g3. The bars and error bars represent the mean ± s.d. of *n* = 3 biological replicates, shown as dots. Data shown are the same as presented in Fig. 5b. **h**, Genome-wide specificity to attH1 of Dn29 and key variants fused to dCas9, targeting attH1 with H1-g3. Data shown combine replicates transfected with WT attP and e-attP. **i**, Representative replicates of genome-wide specificity profiles of Dn29 and key variants fused to dCas9. Orange dots represent the on-target locus (attH1), and gray dots represent off-target loci. Data shown for Dn29 are the same as presented in Fig. 1b. **j**, Schematic of the LSR−dCas9 tetrameric complex, with multiplexed sgRNAs targeting the genome upstream and downstream of the pseudosite. **k**, Heatmap showing attH3 integration efficiencies (%) of Dn29−dCas9 using guides targeting upstream and downstream of the pseudosite, individually and multiplexed. Each cell represents the mean of *n* = 3 biological replicates. **l**, Schematic of the LSR−dCas9 tetrameric complex with a single sgRNA targeting both the genome and the donor plasmid. **m**, Integration efficiencies of Dn29−dCas9 using donor plasmids with the H1-g3 sgRNA target sequence adjacent to the attP. Plasmid schematics show sgRNA target placement (5’ or 3’, top or bottom strand). The bars and error bars represent the mean ± s.d. of *n* = 3 biological replicates, shown as dots. Asterisks show *t*-test significance compared to WT donor plasmid. *one-tailed *P* < 0.05, **one-tailed *P* < 0.01. Exact *P* values from top to bottom: *P* = 0.0155, *P* = 0.0031, *P* = 0.0130, *P* = 0.0462.

We reasoned that the LSR protein and target DNA interaction could be further enhanced by developing LSR−dCas9 fusions that facilitate LSR recruitment to the genomic target site, as demonstrated in other recombinase systems^3,4,6,19,22^. We first optimized the fusion design, observing that an N-terminal dCas9 fusion abolished Dn29 activity, likely due to steric hindrance of tetramerization as the N terminus is located at the tetrameric complex core^29,36^. By contrast, C-terminal fusions supported robust recombination and were used for further experiments (Fig. 3a).

**Extended Data Fig. 5:**
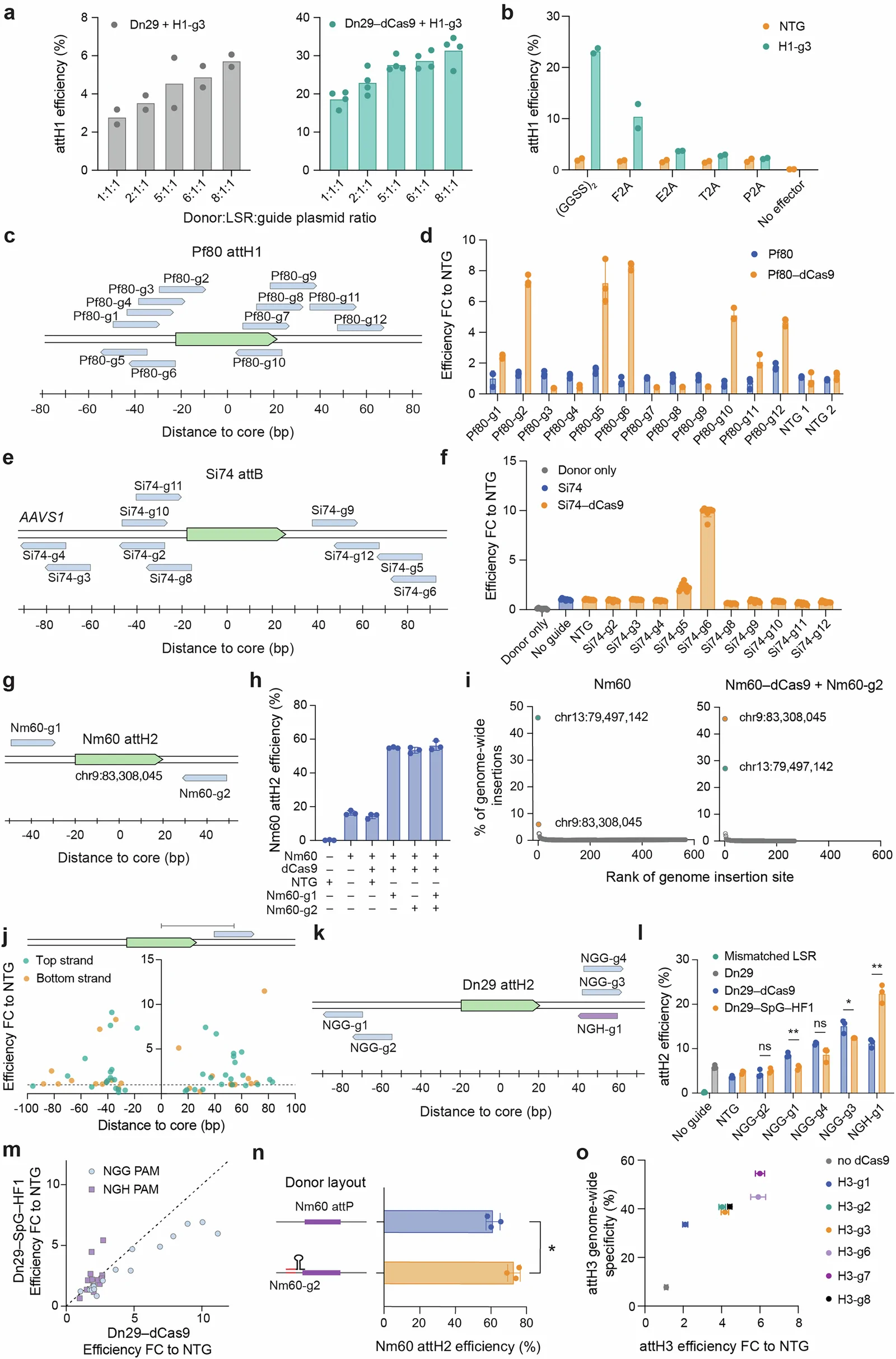
Versatility and optimization of LSR−dCas9 fusions across different recombinases and genomic targets. **a**, attH1 integration efficiency of donor:effector:guide plasmid stoichiometries for Dn29 (left, *n* = 2 biological replicates) and Dn29−dCas9 (right, *n* = 4 biological replicates). **b**, attH1 integration efficiency of Dn29−dCas9 with direct fusion or 2A peptide linkers, with H1-g3 and non-targeting sgRNA (NTG). 2A peptides ranked in order from least to most complete ribosomal skipping. *n* = 2 biological replicates. **c**, Schematic of sgRNA targets for Pf80 attH1 pseudosite (chr11:64,243,293). **d**, Integration efficiency at Pf80 attH1 pseudosite by Pf80 and Pf80−dCas9, shown as fold change to NTG. Bars and error bars represent the mean ± s.d. of *n* = 3 biological replicates. **e**, Schematic of sgRNA targets for Si74 attB, pre-inserted at the *AAVS1* locus. **f**, Integration efficiency at *AAVS1* Si74 attB with Si74 and Si74−dCas9, shown as fold change to NTG. Bars and error bars represent the mean ± s.d. of *n* = 3 biological replicates. Dots represent the 3 technical replicates per biological replicate. **g**, Schematic of sgRNA targets for the Nm60 attH2 pseudosite (chr9:83,308,045). **h**, Integration efficiency of Nm60−dCas9 at attH2 with guides targeting upstream and downstream of the pseudosite, in single and multiplex. Bars and error bars indicate mean ± s.d. of *n* = 3 biological replicates. **i**, Genome-wide specificity of Nm60 and Nm60−dCas9 with Nm60-g2 sgRNA targeting Nm60 attH2. **j**, Integration efficiency (fold change to NTG) of all LSR−dCas9 fusions (Dn29−dCas9, Pf80−dCas9, Si74−dCas9, Nm60−dCas9) at all pseudosites/gRNAs tested in Fig. 3 and Extended Data Fig. 5, relative to the sgRNA distance to the pseudosite core. The distance to the core is measured as the number of bases between the center of the pseudosite core dinucleotide to the center (11th base) of the 23 bp sgRNA target sequence + PAM sequence. The dots represent the mean of *n* = 2−6 biological replicates. **k**, Schematic of sgRNA targets for Dn29 attH2 pseudosite (chr10:58,514,256). Blue targets: NGG PAMs; purple target: NGH PAM. **l**, Absolute integration efficiency of Dn29−dCas9 compared to Dn29−SpG−HF1 at attH2. Bars and error bars represent the mean ± s.d. of *n* = 3 biological replicates, shown as dots. Asterisks show *t*-test significance. * = two-tailed *P* < 0.05; ** = two-tailed *P* < 0.01; ns = not significant. Exact *P* values from left to right: *P* = 0.3268, *P* = 0.0032, *P* = 0.0522, *P* = 0.0346, *P* = 0.0010. **m**, Correlation of Dn29−dCas9 vs. Dn29−SpG−HF1 integration efficiency with NGG or NGH PAM sgRNAs. Dots represent the mean of *n* = 3 biological replicates, with each dot representing a unique sgRNA. Dotted line represents the identity line. **n**, Integration efficiency of Nm60−dCas9 at attH2 with a donor-binding sgRNA (Nm60-g2) plasmid. Bars and error bars represent the mean ± s.d. of *n* = 3 biological replicates. Asterisks show *t*-test significance. *=one-tailed *P* < 0.05. **o**, Correlation between integration efficiency (fold change to NTG, *n* = 3 biological replicates) and genome-wide specificity at attH3 (*n* = 2 biological replicates) of various attH3-targeting sgRNAs. Dots and error bars represent the mean ± s.d. of samples with *n* ≥ 3 biological replicates. Data shown is the same as presented in Figs. 3e and 3f.

We next evaluated the impact of dCas9-mediated genomic recruitment using single guide RNAs (sgRNAs) targeting regions near attH1 or attH3 (Fig. 3b,c). This single-guide approach increased integration efficiency 2.7-fold at attH1 and six-fold at attH3 compared to non-targeting guides (NTGs) (Fig. 3d,e). We further improved efficiency by optimizing the ratio of donor, effector and guide components (Extended Data Fig. 5a) and confirmed the importance of direct tethering of Dn29 to the genomic target site, as linker replacement with a P2A peptide to induce ribosomal skipping abolished the effect^37,38^ (Extended Data Fig. 5b).

Interestingly, we could manipulate the natural integration preference of Dn29 using dCas9 recruitment. Although WT Dn29 naturally integrates into attH1 with two-fold frequency over attH3, dCas9-based recruitment to attH1 amplified this preference to 14-fold. Conversely, attH3-targeting sgRNAs reversed this bias, resulting in 11-fold higher integration at attH3 compared to attH1 (Fig. 3f). Combining dCas9 fusions with our optimized Dn29 variants further improved on-target efficiency. SuperDn29−dCas9 achieved 50.8% integration at attH1, whereas goldDn29−dCas9 and hifiDn29−dCas9 reached 44.5% and 39.1%, respectively, representing up to an 11.8-fold efficiency increase (Fig. 3g).

Next, we tested if dCas9 fusions enhanced specificity by biasing insertion toward the desired pseudosite. Whole-genome insertion profiling showed that on-target integrations improved from 12% with WT Dn29 to more than 60% with Dn29−dCas9 targeted to attH1, although rare off-target sites persisted. SuperDn29−dCas9 maintained a similar insertion profile but moderately increased rare off-targets due to higher overall activity. Notably, goldDn29−dCas9 and hifiDn29−dCas9 achieved 91% and 97% genome-wide specificity to attH1, with significantly fewer off-target loci (average 35 and 12 sites, respectively) (Fig. 3h,i and Supplementary Figs. 3 and 4). For Dn29−dCas9, 20−35% of off-target sites were shared between replicates, decreasing to 6−12% for superDn29−dCas9 and to 0% for goldDn29−dCas9 and hifiDn29−dCas9 (Supplementary Fig. 5a−d and Supplementary Table 3). Off-target site concordance across variants was low, with only 15 sites appearing in more than one sample (Supplementary Fig. 5e). Furthermore, these off-target sites did not overlap with predicted guide RNA off-target sites, indicating that they are not dCas9 mediated (Supplementary Fig. 5f). Taken together, these results suggest that specificity engineering has effectively eliminated reproducible off-target activity, with the low reproducibility potentially reflecting both rare genuine events and technical artifacts previously reported for this off-target detection method^39^.

We assessed the generalizability of our fusion approach across three additional LSR orthologs (the genome-targeting LSRs Pf80 and Nm60 and the landing pad LSR Si74), demonstrating up to 10-fold improved efficiency (Extended Data Fig. 5c−i). To elucidate optimal sgRNA design parameters, we analyzed integration efficiencies across all fusion variants and orthologs and determined optimal sgRNA placement to be approximately 40 bp from the attachment site core, agnostic of guide orientation (Extended Data Fig. 5j).

We next explored three orthogonal strategies to further improve efficiency—using dCas9 variants with alternative PAM recognition^40^, multiplexing sgRNAs and incorporating sgRNA binding sites on donor plasmids. dCas9 variants with increased PAM flexibility can expand pseudosite-proximal guide options. Using dCas9−HF1−SpG (NGN PAM), we achieved 22% insertion efficiency at the best NGH guide compared to 15% with the best NGG guide (Extended Data Fig. 5k−m). Multiplexed sgRNAs targeting upstream and downstream of the attachment site could further improve genome search and binding (Fig. 3j). At the attH3 site, the H3-g3 and H3-g7 combination achieved 13% integration versus 6.6% and 5.6% efficiencies individually (Fig. 3k). Finally, we tested the ability of an sgRNA-binding sequence on the donor plasmid to facilitate donor recruitment, across four orientations flanking the minimal attP site (top and bottom strand, upstream and downstream) (Fig. 3l). All four configurations improved integration efficiency for Dn29−dCas9, reaching up to 40% integration compared to 23% with the WT donor (Fig. 3m). Similarly, incorporating the Nm60-g1 sequence on the donor plasmid improved Nm60 attH2 integration from 61% to 73% (Extended Data Fig. 5n).

Our results show that LSR−dCas9 fusions can substantially enhance both efficiency and specificity of cargo DNA insertion by improving LSR recruitment to target and donor DNA. The positive correlation between efficiency and specificity (Extended Data Fig. 5o) suggests that optimizing and multiplexing guide RNAs are crucial for maximizing both parameters simultaneously. This approach, combined with engineered LSR variants and dual-targeting guides, achieves up to 97% specificity or over 73% efficiency at a single genomic locus.

### Exploring attP sequence space enables the design of optimized donor DNA

**Fig. 4:**
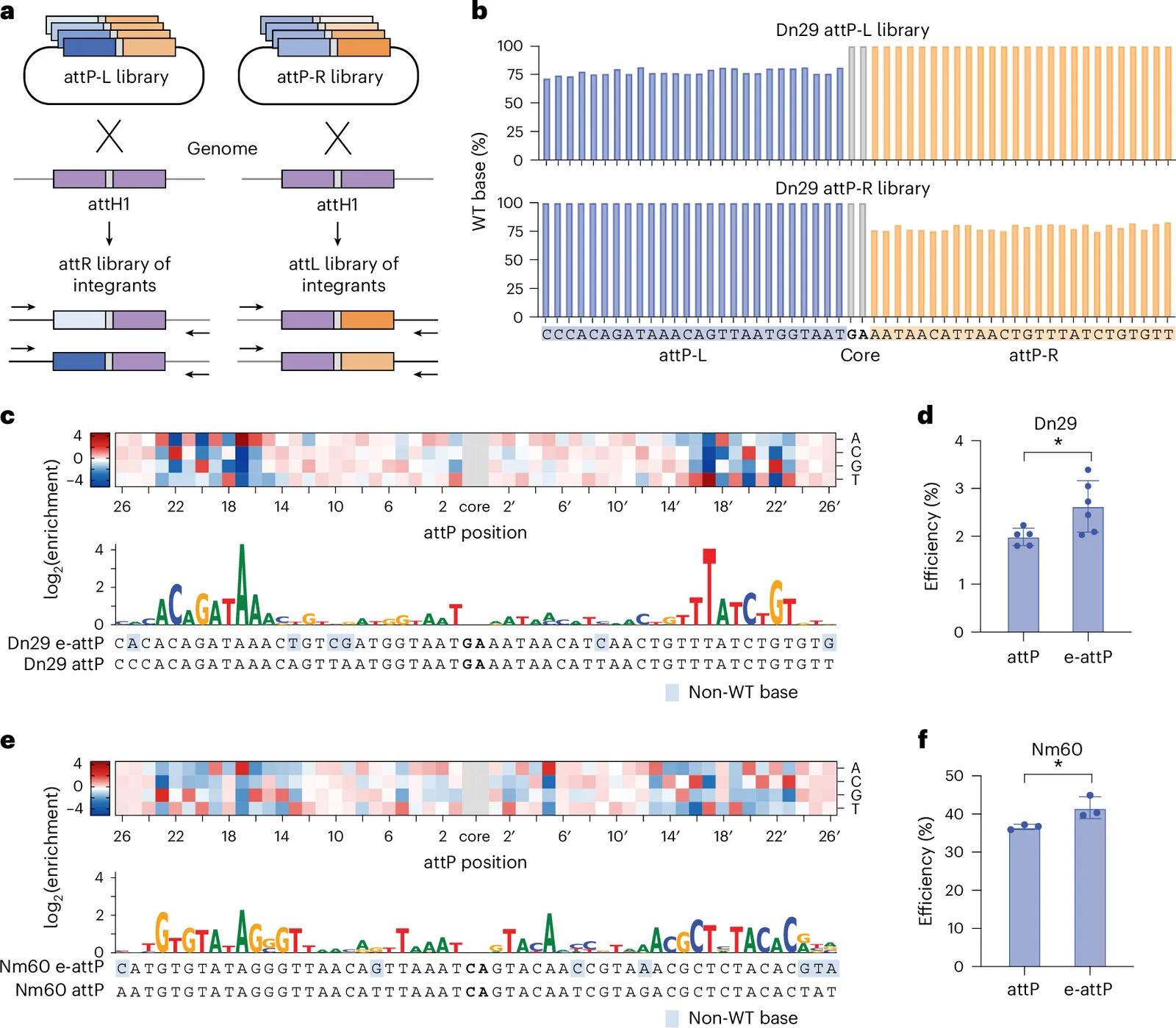
Exploring attP sequence space enables the design of optimized donor DNA. **a**, Schematic of attP optimization screen. attP-L and attP-R libraries are transfected into HEK293FT cells with Dn29−dCas9, and integrants are sequenced. **b**, Design of attP-L and attP-R libraries. Libraries are generated using mixed base oligos containing 79% WT and 7% each other nucleotide for one half-site and constant WT sequence for the other half-site. **c**, Dn29 attP nucleotide enrichment/depletion heatmap (top) and sequence logo of enriched nucleotides in e-attP (bottom). Data represent average enrichment scores of two biological replicates. **d**, Dn29 integration efficiency with attP and e-attP donor plasmids. The bars and error bars represent the mean ± s.d. of *n* = 5 (attP) and *n* = 6 (e-attP) biological replicates, shown as dots. Asterisks show *t*-test significance compared to WT donor. *one-tailed *P* = 0.0165. **e**, Nm60 attP nucleotide enrichment/depletion heatmap (top) and sequence logo of enriched nucleotides in e-attP (bottom). Data represent average enrichment scores of two biological replicates. **f**, Nm60 integration efficiency with attP and e-attP donor plasmids. The bars and error bars represent the mean ± s.d. of *n* = 3 biological replicates, shown as dots. Asterisks show *t*-test significance compared to WT donor. *one-tailed *P* = 0.0208.

Beyond dCas9 fusions, we explored enhancing donor DNA recruitment by optimizing the attP sequence. We created two Dn29 attP donor plasmid libraries, each with one constant half-site and the other mutated using custom mixed base oligos for an average of approximately 5.5 mutations per 26-bp half-site (Methods). Transfecting these libraries into cells expressing WT Dn29−dCas9 and H1-g3 guide, we sequenced attH1 integrants and calculated nucleotide enrichment scores (Fig. 4a−c). Although the WT nucleotide was generally preferred, we identified six positions where non-WT nucleotides showed moderately higher enrichment. Combining all six substitutions into an optimized e-attP sequence improved integration efficiency by 1.3-fold (Fig. 4d). Applying this strategy to Nm60 with Nm60−dCas9 and Nm60-g2, we identified an e-attP with seven mutations that improved efficiency by 1.1-fold (Fig. 4e,f).

Our library enrichment approach provides a high-resolution view of DNA specificity and recombination efficiency determinants. For both Dn29 and Nm60, native attachment sites appear highly evolutionarily optimized, with only a few positions showing incremental efficiency improvements through mutation.

We identified core-distal regions of outsized importance for functional recombination: positions 16−23 and 16’−23’ for Dn29 (Fig. 4C) and 13−23 and 13’−23’ for Nm60 (Fig. 4e). These findings are consistent with previous reports on other LSR orthologs that show stronger zinc-ribbon domain binding requirements and more relaxed sequence specificity in the recombinase domain^41,42^. Dn29’s position 17/17’ showed particularly high enrichment, strongly preferring A and T, respectively. Furthermore, analysis of depleted nucleotides reveals selection against attB-like sequences, particularly A/T at position 12/12’ for Dn29 and A/T at position 14/14’ for Nm60, which correspond to critical attB nucleotides (Supplementary Fig. 6a,b)^12^. This depletion suggests evolutionary pressure to maintain discrimination between attB and attP sites via previously described ‘discriminator bases’^41^. By analyzing dinucleotide normalized to single-nucleotide abundance, we identified preferential RY dinucleotides at positions 17/18 and YR dinucleotides at positions 22/23, suggesting that DNA flexibility as well as sequence contributes to protein recognition of the attachment site^43–46^ (Supplementary Fig. 6c).

Given the numerous DNA-binding domain mutations in our variants, we applied this approach to identify optimal attP substrates for each recombinase. The engineered variants preferred attP sequences with 7−9 mutations relative to WT Dn29 e-attP, predominantly favoring guanine/cytosine bases (Supplementary Fig. 7a−c). This G/C bias likely reflects enhanced lysine−guanine interactions resulting from the numerous specificity-enhancing lysine residues incorporated into the DNA-binding regions during protein engineering^47^. Although variant-specific e-attPs demonstrated modest improvement over the WT e-attP sequence (Supplementary Fig. 7d), we used the WT e-attP for all subsequent studies to maintain a standardized donor template across all variants.

Although LSR attachment sites are canonically imperfect inverted repeats, the importance of this asymmetry remains unclear^42^. Our data reveal that the most strongly enriched nucleotides are symmetrical across the core, aligning with previous Bxb1 studies^48,49^. However, for Dn29 and Nm60, we observed five and eight nucleotides with subtle preferences for asymmetric nucleotides at corresponding half-site positions. This suggests that slight attachment site asymmetry may be a deliberate feature of the recombination mechanism rather than a consequence of mutations or phage genome sequence constraints. Overall, this attachment site exploration deepens our understanding of LSR target site recognition and advances our ability to design optimal DNA donors.

### Unifying engineering strategies for maximal LSR efficiency and specificity

**Fig. 5:**
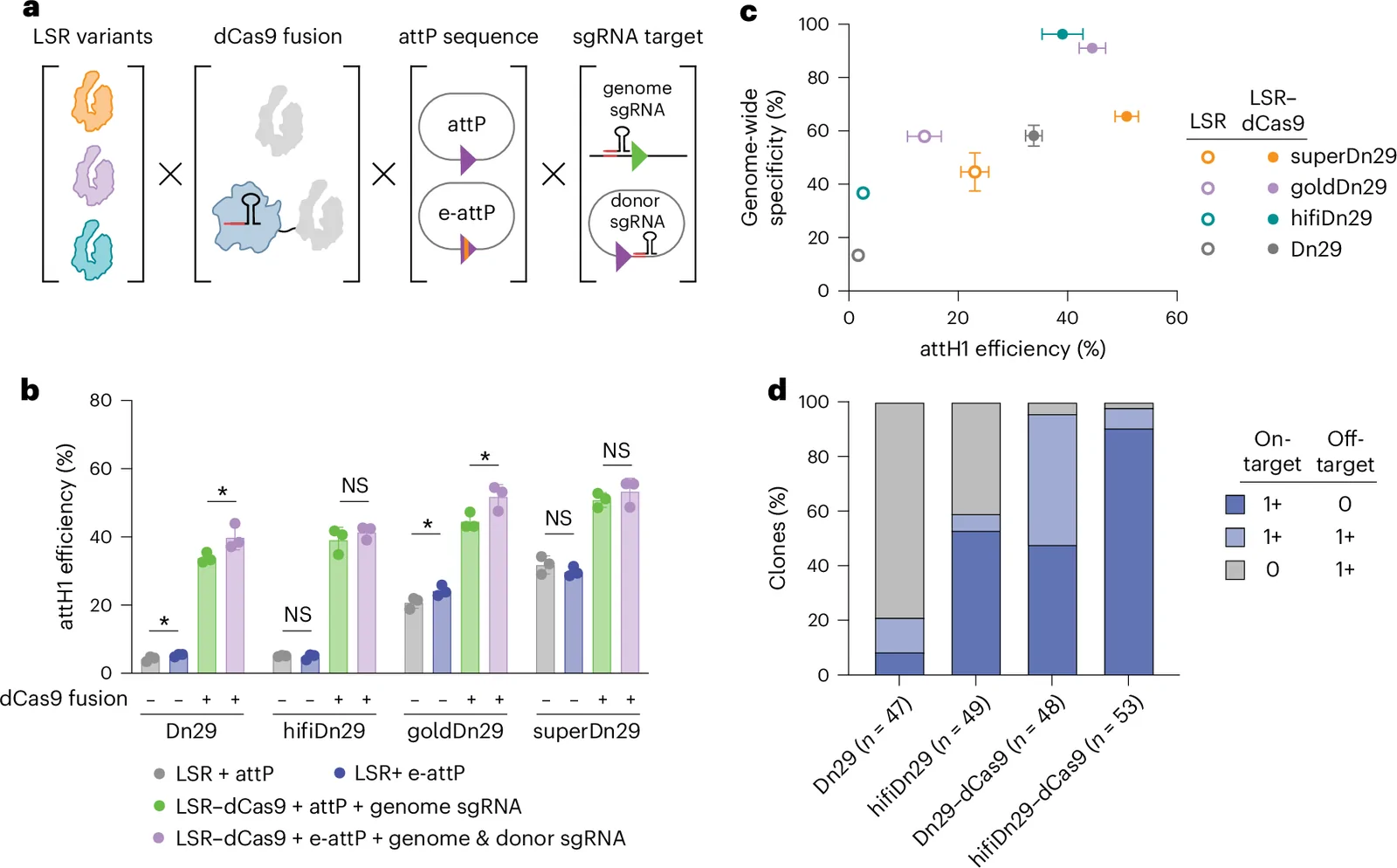
Unifying engineering strategies for maximal LSR efficiency and specificity. **a**, Schematic overview of developed engineering strategies. **b**, Integration efficiencies at attH1 of Dn29 with combined engineering strategies. The bars and error bars represent the mean ± s.d. of *n* = 3 biological replicates, shown as dots. Asterisks show *t*-test significance. *one-tailed *P* < 0.05; NS, not significant. Exact *P* values from left to right: *P* = 0.0445, *P* = 0.0278, *P* = 0.3161, *P* = 0.1991, *P* = 0.0297, *P* = 0.0230, *P* = 0.1555, *P* = 0.1991. **c**, Integration efficiency and genome-wide specificity of engineered LSRs at attH1, with and without dCas9 fusions. *n* = 2−4 specificity biological replicates and *n* = 3 efficiency biological replicates. Dots and error bars represent the mean ± s.d. of samples with *n* ≥ 3 biological replicates. **d**, Specificity analysis of edited HEK293FT single-cell clones engineered with Dn29 and hifiDn29, with and without dCas9 fusions. Number (*n*) of clones analyzed per sample is indicated on the *x* axis.

Next, we aimed to create optimal LSR tools for large DNA cargo integration by combining our orthogonal engineering efforts. Armed with directed evolution variants, dCas9 fusions with sgRNA design rules and optimized donor DNA substrates, we assessed combining these features into a single system (Fig. 5a,b). Overall, our combined engineering strategies substantially improved recombination efficiency: the variants fused to dCas9 with the optimized donor achieved 41−53% efficiency, a 9.6-fold to 12.3-fold improvement over the WT enzyme (Fig. 5b and Supplementary Table 4).

**Extended Data Fig. 6:**
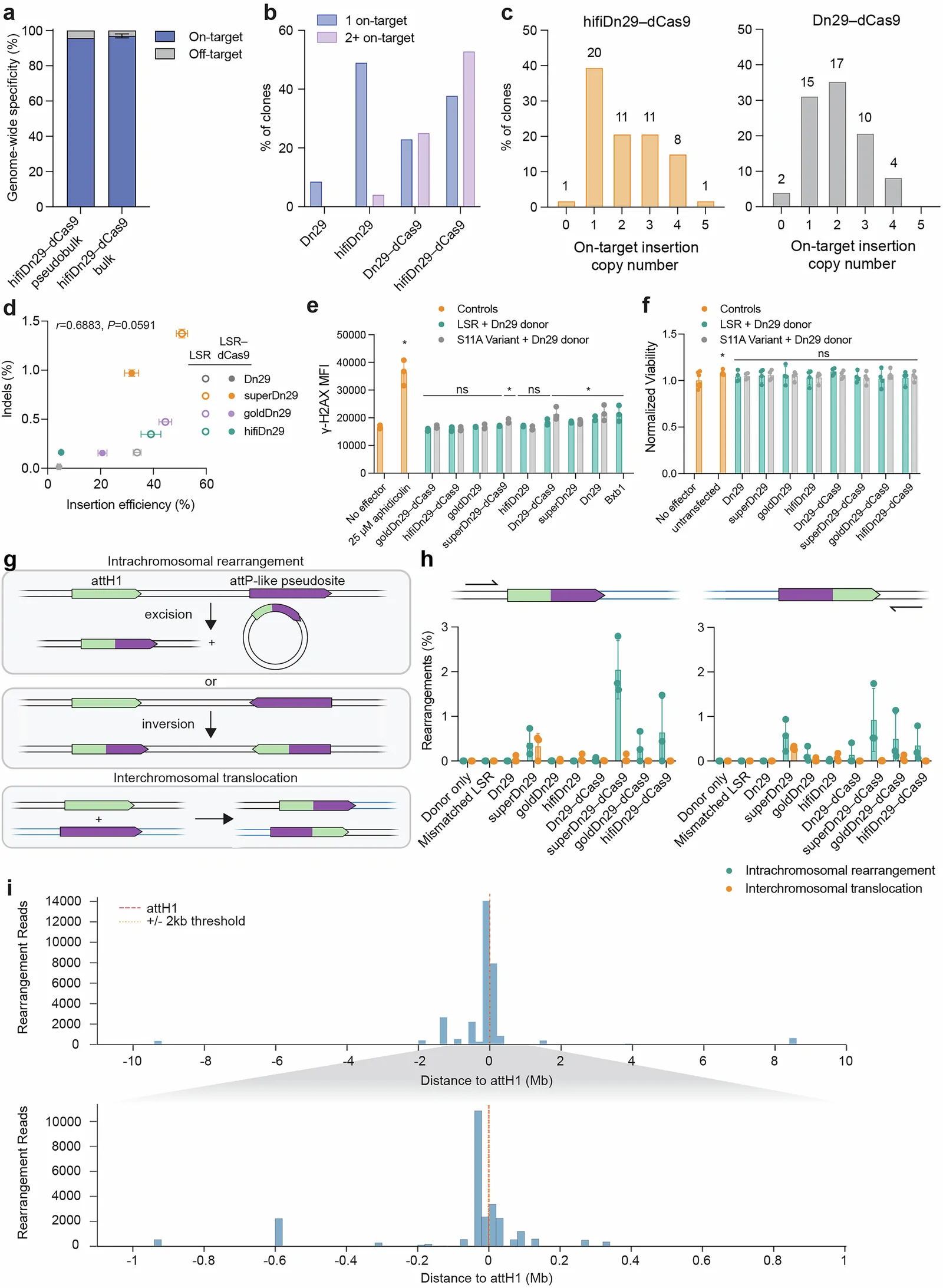
Characterization of integration copy number and undesired editing outcomes. **a**, Comparison of hifiDn29−dCas9 on-target and off-target integrations: single cell clonal genotyping (*n* = 53 clones, left) vs bulk genome-wide integration assay (mean ± s.d., *n* = 3 biological replicates, right). **b**, On-target insertion copy number per clone for hifiDn29 and Dn29, with and without dCas9 fusion. **c**, On-target insertion copy number per clone for hifiDn29−dCas9 and Dn29−dCas9. Number (*n*) of clones is labeled above each bar. **d**, Correlation of attH1 insertion efficiency by ddPCR and rate of indel formation at attH1 by NGS. Data represents mean ± s.d. of *n* = 3 biological replicates. **e**, Genome-wide γ-H2AX staining and flow cytometry, measured 2 days after transfection with LSR and donor plasmids. S11A variants have an alanine mutation in the catalytic serine. 25 μM aphidicolin was included as a positive control. Bars and error bars represent the mean ± s.d. of *n* = 3 biological replicates. Asterisks and *P* values show *t*-test significance compared to no effector control. **=two-tailed *P* < 0.01. Exact *P* values are provided in Table S5. **f**, Viability of HEK293FT cells, 2 days after transfection with LSR and donor plasmids. Bars and error bars represent the mean ± s.d. of *n* = 4 biological replicates. Asterisks show *t*-test significance compared to no effector control. *=two-tailed *P* < 0.05. Exact *P* values are provided in Table S5. **g**, Schematic of off-target genome rearrangement outcomes. Recombination between attH1 and an attP-like pseudosite on the same chromosome could lead to intrachromosomal rearrangements resulting in either excision or inversion, depending on attachment site orientation. Recombination between attH1 and an attP-like pseudosite on different chromosomes would lead to a translocation. **h**, Quantification of interchromosomal translocations and intrachromosomal rearrangements after transfection with LSR and donor plasmids, with NGS baited upstream (left panel) or downstream (right panel) of attH1. Bars and error bars represent the mean ± s.d. of *n* = 3 biological replicates. **i**, Distance of intrachromosomal rearrangements to attH1, aggregating all rearrangement reads from the downstream-baited samples. Top panel shows distribution of all rearrangements across the entire chromosome. Bottom panel shows a magnified view of rearrangements within 1 Mb upstream and downstream of attH1.

Using hifiDn29−dCas9, our most specific configuration, we measured 97% genome-wide specificity by bulk integration site sequencing (Fig. 5c). To better understand the single-cell variation of insertional mutagenesis, including on-target/off-target co-occurrence and integration copy number, we mapped integrations in approximately 50 clonal HEK293FT populations edited with hifiDn29 or WT Dn29, with and without dCas9 fusions (Fig. 5d). dCas9 fusion dramatically improved performance for both Dn29 and hifiDn29, resulting in over 95% of clones containing on-target edits. However, the most striking difference was observed in off-target insertions: 91% of Dn29 clones contained off-target insertions compared to 46% of hifiDn29 clones. Ultimately, with dCas9 fused to hifiDn29, off-target insertions were reduced to only 9% of clones compared to 52% for Dn29–dCas9. These single-cell specificity measurements closely mirrored the bulk genome-wide specificity results (Extended Data Fig. 6a).

Beyond targeting accuracy, measuring the number of integrations per cell is crucial for fully understanding editing outcomes. Because dCas9 increases efficiency, it also increases the rate of multiple on-target insertions. HifiDn29 showed the highest rate of single on-target insertion events, with half of the clones exhibiting this genotype. By contrast, dCas9 fusions decreased single on-target insertion events to 38% and increased the rate of multiple on-target insertions from 4% to 53% of clones (Extended Data Fig. 6b). Due to the pseudo-triploid genome and copy number variation/instability of HEK293FT cells^50^, on-target insertions ranged from zero to five per cell, with a median of two for both hifiDn29−dCas9 and Dn29−dCas9 clones (Extended Data Fig. 6c). Overall, these single-cell results demonstrate the enhanced precision and efficiency of hifiDn29−dCas9, nominate hifiDn29 for generating clonal cell lines containing single on-target integrations and highlight the value of single-cell analysis in evaluating gene editing outcome heterogeneity.

### Characterization of undesired editing outcomes

Genome editing can pose safety risks through unintended outcomes, including insertion and deletion (indel) formation, cytotoxicity and genomic rearrangements. Because the recombinase mechanism involves coordinated cleavage of all four DNA strands, abortive recombination could potentially lead to double-stranded break (DSB) formation, causing indels at the attachment sites^51–53^. We identified rare but significant indels at attH1 (0.01−1.4%), with indel frequency correlating with recombination efficiency (Extended Data Fig. 6d). This trend parallels observations in small serine recombinase systems, where activating mutations that increase synapse formation also demonstrate higher rates of DSB generation^54^. As a proxy for genome-wide DSB formation or DNA damage response (DDR) activation, we employed phosphorylated-H2AX (γ-H2AX) staining and flow cytometry. Dn29 and superDn29 showed low but significant γ-H2AX, whereas specificity-enhanced variants (goldDn29 and hifiDn29) reduced damage to background levels (Extended Data Fig. 6e). Notably, these variants also generated less γ-H2AX than Bxb1, previously reported as the highest-fidelity recombinase^51^ and commonly used in attachment site prime editing approaches such as PASTE and PASSIGE^8,10,39^. Interestingly, delivering catalytically dead LSR also produced γ-H2AX, suggesting that non-catalytic mechanisms such as DNA binding may damage DNA (Extended Data Fig. 6e and Supplementary Table 5).

Cytotoxicity can arise from multiple mechanisms, including off-target effects, DDR activation, immune activation, protein burden and genomic instability. Cell viability assays revealed no significant toxicity in HEK293FT cells when transfecting LSR variants and donor plasmid (Extended Data Fig. 6f). Measuring genome-wide junctions with attH1 by NGS, we detected genomic rearrangements ranging between 0% and 2% within chromosome 10 and 0−0.3% inter-chromosomal translocations (Extended Data Fig. 6g,h). Most junctions occurred within 50 kb of attH1, potentially reflecting a preference for recombining with nearby sequences or resulting from DSB-induced large deletions (Extended Data Fig. 6i). Overall, our comprehensive safety assessment demonstrates that Dn29-mediated editing, particularly with our specificity-enhanced variants, offers a favorable safety profile with minimal cytotoxicity, rare genomic rearrangements and reduced DNA damage compared to existing recombinases, positioning this system as a promising option for precise genomic integration.

### Engineered LSR systems insert multi-kilobase DNA cargo into the genome of non-dividing cells, human embryonic stem cells and primary T cells

**Extended Data Fig. 7:**
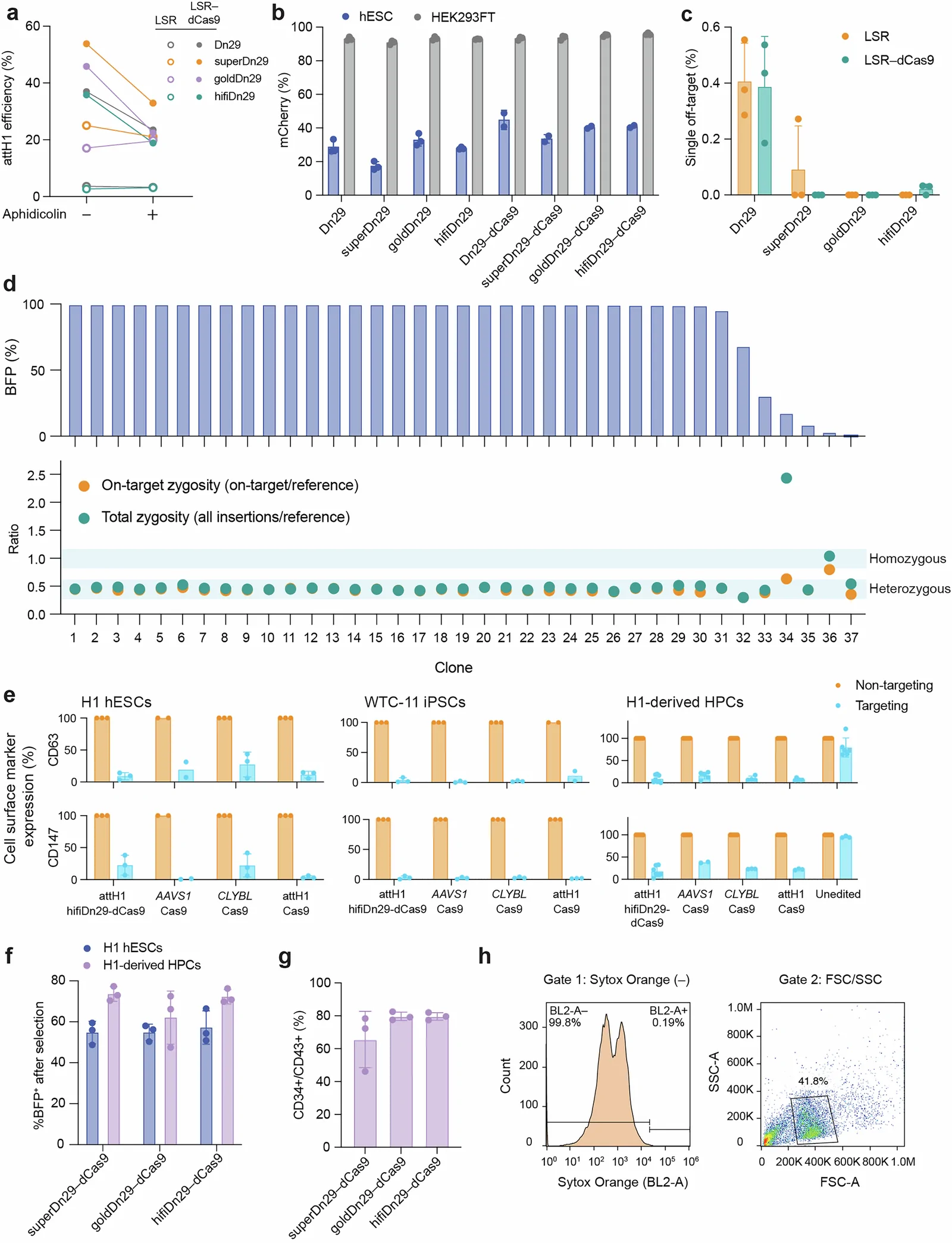
Engineered LSR systems applied to non-dividing cells, human embryonic stem cells, and differentiated HPCs. **a**, Integration efficiencies of Dn29 variants and dCas9 fusions at attH1, with and without cell cycle arrest by aphidicolin treatment. The dots represent the mean of *n* = 3 biological replicates. **b**, Donor plasmid transfection efficiency in HEK293FTs and hESCs (% mCherry^+^ cells). Bars and error bars represent the mean ± s.d. of *n* = 3 biological replicates, shown as dots. **c**, Specificity of Dn29 and variants in hESCs, measured as attH3 off-target integration efficiency by ddPCR. Bars and error bars represent the mean + s.d. of *n* = 3 biological replicates, shown as dots. **d**, H1 hESC clones (*n* = 37) edited with goldDn29−dCas9: BFP expression (top) and genotyping (bottom). Integration/reference ratio of 0.5 indicates heterozygous insertions, 1 indicates homozygous insertions. Single clone per bar/dot. **e**, Knockdown of cell surface markers CD63 and CD147 after guide transduction and selection, relative to non-targeting guide, in WTC-11 iPSCs, H1 hESCs, and H1-derived HPCs engineered with hifiDn29−dCas9 at attH1 or Cas9 at *AAVS1*, *CLYBL*, and attH1. Plots show the knockdown quantification of *n* = 2−8 biological replicates (mean ± s.d. for samples with *n* ≥ 3), calculated as target/non-target median fluorescence intensity, represented as a percentage. **f**, CRISPRi−BFP cassette expression in engineered hESCs after selection, pre- and post- differentiation into HPCs. Bars and error bars represent the mean ± s.d. of *n* = 3 biological replicates, shown as dots. **g**, HPC differentiation markers (CD34/CD43) of LSR−dCas9-edited hESCs post differentiation. Bars and error bars represent the mean ± s.d. of *n* = 3 biological replicates. **h**, Example gating strategy for HPCs. First, unstained cells are used as a negative control to set the Sytox Orange gate, indicating the boundary between live (BL2-A negative) and dead (BL2-A positive) cells (left). Each sample is first gated for Sytox Orange (−), then gated for HPCs using FSC/SSC. Within this population, the median fluorescence intensity (MFI) of the stained cell surface marker is used for determination of knockdown efficiency.

Next, we sought to benchmark our engineered LSRs in a diverse set of genome insertion tasks across various cell types. LSRs offer an advantage in genome engineering applications, including robust insertion efficiency in non-dividing cells^10^. We treated HEK293FT cells with aphidicolin to induce cell cycle arrest and observed that Dn29 and key variants showed largely equivalent integration rates to untreated cells. dCas9 fusions had decreased integration efficiency by 35−50%, but still achieved up to 30% integration (Extended Data Fig. 7a).

**Fig. 6:**
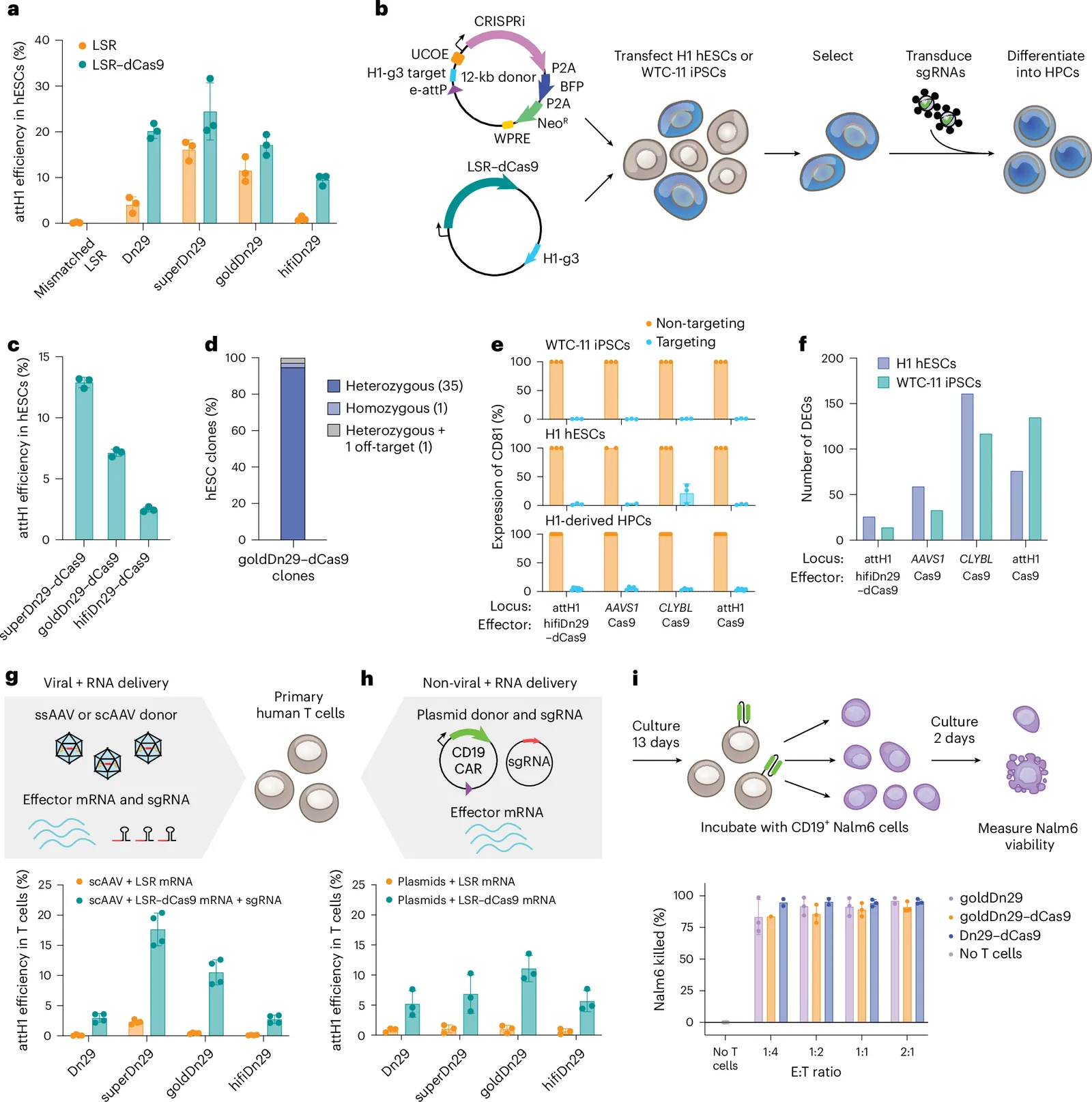
Engineered LSR systems insert large DNA cargo into the genome of stem cells and primary T cells. **a**, Integration efficiencies of Dn29 variants and dCas9 fusions in hESCs. Bars and error bars represent the mean ± s.d. of *n* = 3 biological replicates, shown as dots. **b**, Schematic of hESC engineering with LSRs. A 12-kb CRISPRi donor plasmid and an LSR−dCas9 effector/guide plasmid are transfected into H1 hESCs or WTC-11 iPSCs, selected and differentiated into HPCs. At the stem cell stage or the HPC stage, cells are transduced with lentivirus expressing sgRNAs for CD81, CD63 or CD147 for measuring CRISPRi knockdown. **c**, Integration efficiency of 12-kb CRISPRi donor by LSR−dCas9s at attH1. *n* = 3 biological replicates. **d**, Genotyping hESC single-cell clones engineered with goldDn29−dCas9. Number (*n*) of clones analyzed per sample is indicated in the legend. **e**, CD81 knockdown after guide transduction and selection, relative to NTG, in WTC-11 iPSCs, H1 hESCs and H1-derived HPCs engineered with hifiDn29−dCas9 at attH1 or Cas9 at *AAVS1*, *CLYBL* and attH1. Plots show the knockdown quantification of biological replicates (mean ± s.d.), calculated as target/non-target median fluorescence intensity, represented as a percentage. WTC-11 (*n* = 3), H1/*AAVS1* (*n* = 2), H1/others (*n* = 3), HPCs (*n* = 6). **f**, Number of DEGs from bulk RNA-seq of *n* = 3 biological replicates of each engineered line compared to WT hESCs or iPSCs. DEG significance thresholded at Benjamini–Hochberg FDR-adjusted *P* < 0.05 and log_2_(fold change) > 1. **g**, Top, schematic of ssAAV or scAAV donor and effector mRNA delivery into primary human T cells. Bottom, integration efficiencies of Dn29 variants and dCas9 fusions at attH1 in primary human T cells using scAAV donor. Bars and error bars represent the mean ± s.d. of *n* = 4 biological replicates, each originating from a different blood donor. **h**, Top, schematic of non-viral plasmid delivery of a CD19 CAR, an sgRNA expression plasmid and effector mRNA into primary human T cells. Bottom, integration efficiencies of Dn29 variants and dCas9 fusions at attH1 in primary human T cells using non-viral plasmid and mRNA delivery. Bars and error bars represent the mean ± s.d. of *n* = 3 biological replicates, each originating from a different blood donor. **i**, Top, schematic of in vitro cancer target cell-killing assay. Engineered T cells are cultured for 13 days to allow episomal plasmid dilution and then co-cultured with varying concentrations of CD19^+^ Nalm6 leukemia target cells. After 48 hours of co-culture, remaining Nalm6 cells are quantified. Bottom, percentage of Nalm6 cells killed after 48 hours at various E:T ratios. Bars and error bars represent the mean ± s.d. of samples with *n* ≥ 3 biological replicates (each an average of two technical replicates), with each originating from a different blood donor.

We then tested DNA cargo installation in H1 human embryonic stem cells (hESCs) and observed that on-target integration efficiency with engineered recombinases increased up to six-fold relative to WT Dn29, from 4.1% to 24.5% (Fig. 6a). These improvements are 25−50% of HEK293FT integration efficiencies, likely due to the 50−80% reduction in plasmid transfection efficiency in stem cells (Extended Data Fig. 7b). Insertion specificity also significantly improved, as off-target integration at attH3 for the engineered variants approached the ddPCR detection limit (Extended Data Fig. 7c).

To evaluate larger cargo installation and enable functional genomics applications, we designed a 12-kb CRISPR interference (CRISPRi) construct encoding the ZIM3−dCas9 fusion and multiple regulatory elements and marker genes, including blue fluorescent protein (BFP), neomycin resistance marker, Woodchuck post-transcriptional regulatory element (WPRE) and ubiquitous chromatin opening element (UCOE). We achieved robust insertion efficiencies up to 13% and verified transgene expression after selection in both bulk and clonal lines (Fig. 6b,c and Extended Data Fig. 7d). Genotyping of goldDn29−dCas9-edited clones revealed that 95% of clones possessed precise heterozygous attH1 insertions, with only one homozygous clone and one clone showing both on-target and off-target integrations (Fig. 6d). These results underscore the high clonal consistency achieved, demonstrating a predominance of accurate, single-copy integrations and strong maintenance of cargo gene expression in H1 stem cells.

**Extended Data Fig. 8:**
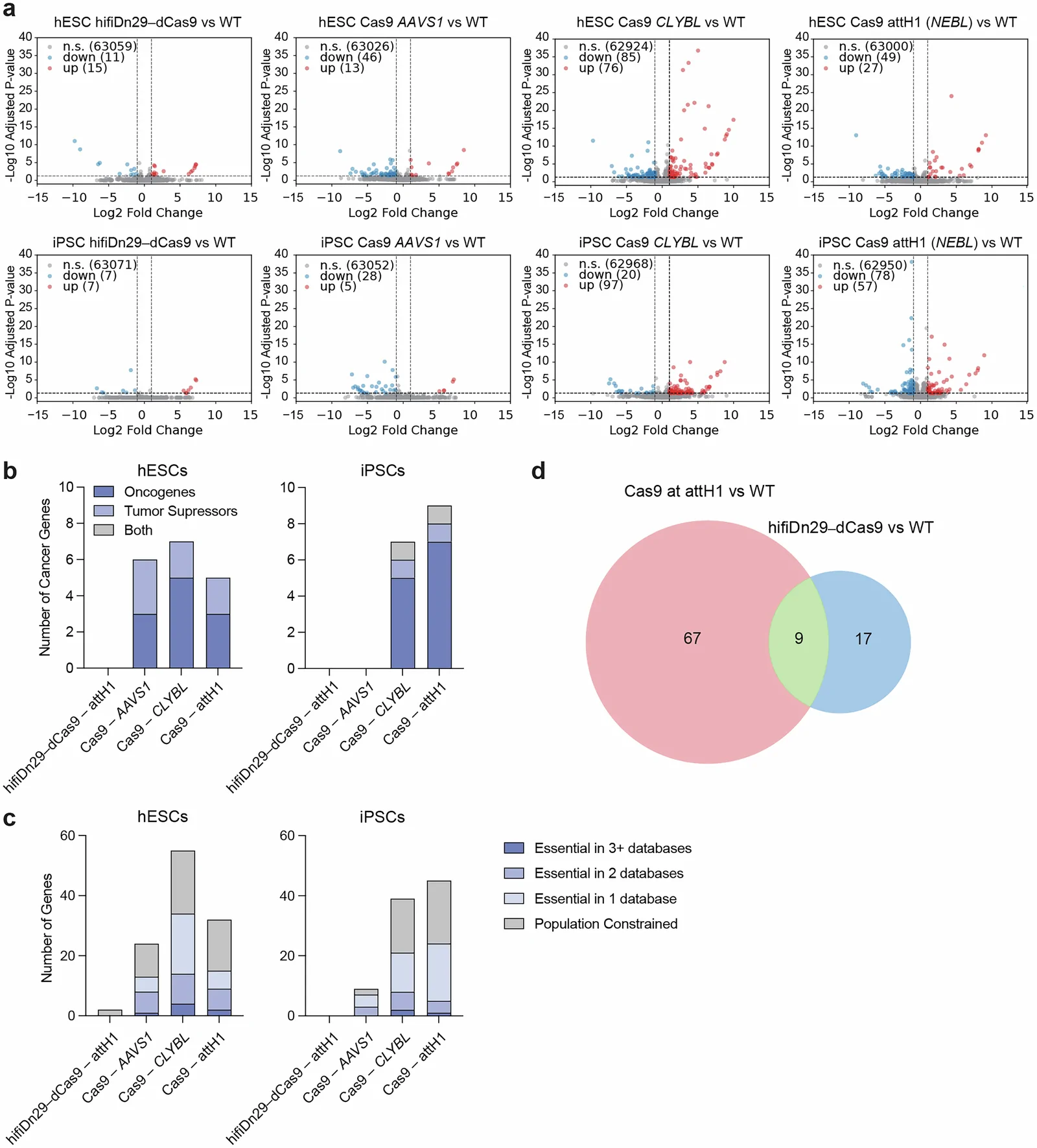
Bulk RNA-seq of hESCs and iPSCs edited with hifiDn29−dCas9 and Cas9 at various genomic loci. **a**, Volcano plots representing differentially expressed genes compared to WT for engineered hESC and iPSC cell lines. Dotted lines indicate significance thresholds, set at Benjamini–Hochberg FDR–adjusted *P* value < 0.05 (two-tailed) and log_2_(fold change) > 1. n.s. = not significant. **b**, Number of DEGs classified as cancer genes based on the OncoKB™ Cancer Gene List^87,88^. **c**, Number of DEGs classified as essential genes. Essential genes were identified using the IMPC Essential Genes Data Portal, which collates five independent databases: IMPC mouse knockout data, DepMap Achilles CRISPR screens, FUSIL cell culture screens, gnomAD population constraint metrics, and ClinGen haploinsufficiency classifications^70,71,89–92^. The following thresholds were applied: IMPC lethal phenotypes, Achilles scores < −0.75, FUSIL lethality, gnomAD pLI > 0.9, and ClinGen sufficient/emerging haploinsufficiency evidence. Genes were classified by the number of databases they appeared essential in, with an additional category for population constraint (gnomAD LoF o/e < 0.35). **d**, Venn diagram showing overlap between DEGs identified in hifiDn29−dCas9 vs Cas9 editing at attH1 in H1 hESCs.

To assess CRISPRi functionality and safety across different genomic loci and editing tools, we compared knockdown efficiency and transcriptome perturbations in H1 hESCs and WTC-11 induced pluripotent stem cells (iPSCs). We tested cells edited at attH1 using either hifiDn29−dCas9 or Cas9 with homology-directed repair (HDR) and at established safe harbors *AAVS1* and *CLYBL* using Cas9 HDR^55–58^. CRISPRi-mediated knockdown of target genes demonstrated similar and robust efficiency across all loci (Fig. 6e and Extended Data Fig. 7e**)**. Bulk RNA sequencing (RNA-seq) analysis revealed that hifiDn29−dCas9 produced the fewest differentially expressed genes (DEGs) compared to Cas9 editing at all loci in both cell types (Fig. 6f and Extended Data Fig. 8a). The DEGs in hifiDn29−dCas9 samples demonstrated the strongest safety profile, with no disruption of oncogenes, tumor suppressor genes or essential genes (Extended Data Fig. 8b−d). Critically, *NEBL* expression showed no significant change across all edited cell lines, confirming that attH1 integration does not disrupt the endogenous locus (Supplementary Tables 6 and 7).

Transgene silencing is a persistent challenge in stem cell engineering, likely due to extensive chromatin restructuring that occurs during differentiation^59^. To assess whether attH1 supports stable cargo expression during differentiation, we differentiated the CRISPRi-edited hESCs into hematopoietic progenitor cells (HPCs) under neomycin selection. After differentiation, edited HPCs maintained robust cargo expression (approximately 70% BFP^+^) with approximately 80% of cells expressing canonical HPC markers, such as CD34 and CD43 (Extended Data Fig. 7f−h). Next, we transduced sgRNAs targeting CD63, CD81 and CD147 into the edited HPCs and observed 82−96% knockdown of these cell surface markers compared to a non-targeting control, with knockdown rates similar to *AAVS1* and *CLYBL* sites (Fig. 6e and Extended Data Fig. 7e). Taken together, we demonstrate that engineered recombinases efficiently produce bulk hESC lines with near-clonal genotypes, minimal transcriptomic perturbation and stable large cargo expression throughout hESC-to-HPC differentiation, making them suitable for generating stable cell lines for CRISPR screens or potentially introducing therapeutic genetic cargoes.

**Extended Data Fig. 9:**
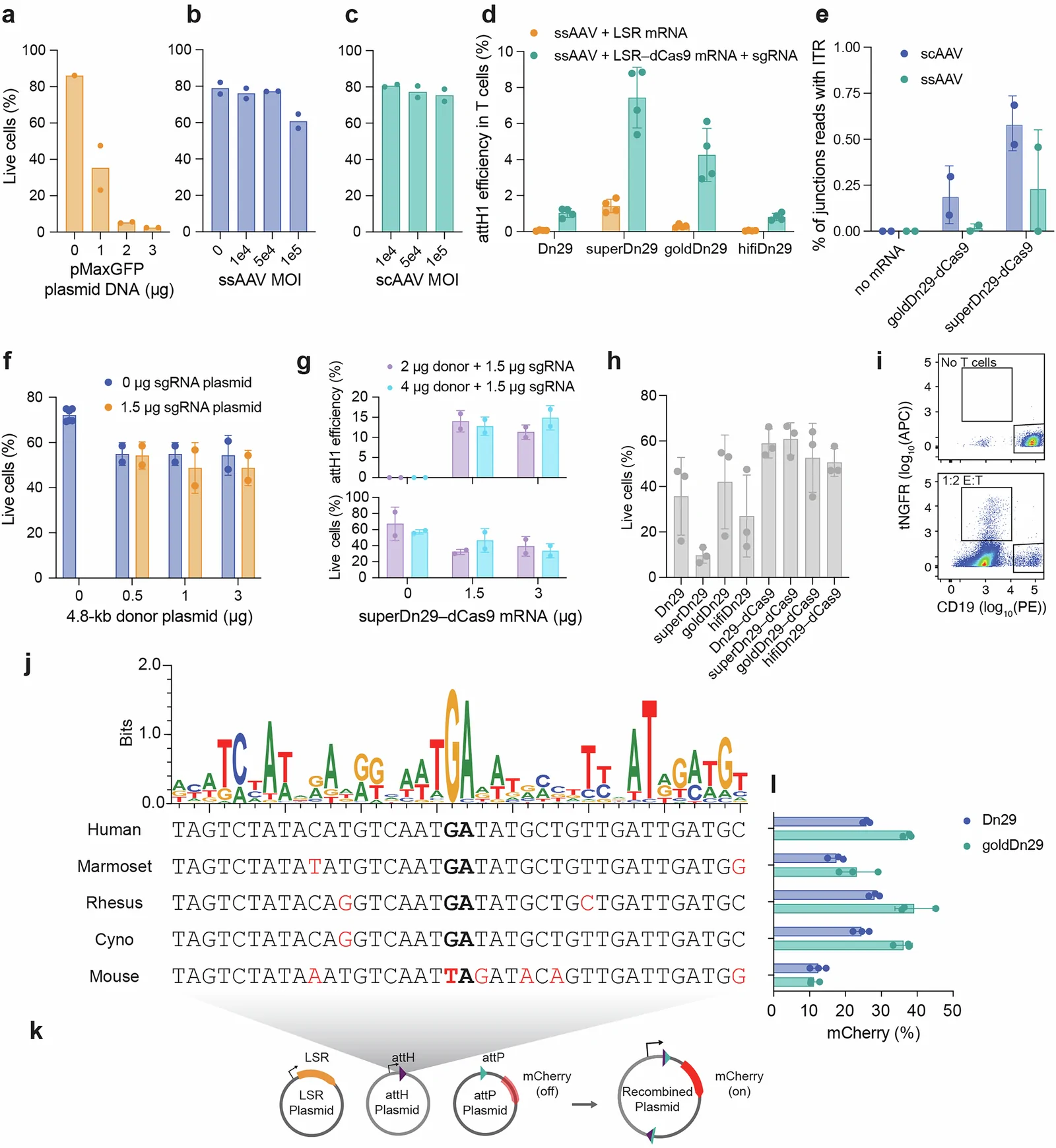
T cell engineering and cross-species compatibility of engineered recombinases. **a**−**c**, Viability of primary T cells upon (**a**) unoptimized electroporation with Lonza pMaxGFP plasmid DNA, (**b**) transduction with ssAAV, and (**c**) transduction with scAAV. *n* = 2 biological replicates from separate blood donors. **d**, Integration efficiencies of Dn29 variants and dCas9 fusions at attH1 in primary human T cells using ssAAV donor. Bars and error bars represent the mean ± s.d. of *n* = 4 biological replicates, each originating from a different blood donor. **e**, Quantification of ITR sequences at attH1-donor junctions, indicating AAV genome capture versus LSR-mediated integration. Bars and error bars represent the mean ± s.d. of *n* = 2 biological replicates. **f**, Viability of human primary T cells one day after electroporation of donor and sgRNA plasmids using optimized plasmid electroporation protocol (Methods). Bars and error bars represent the mean ± s.d. of *n* = 2 biological replicates, each originating from a different blood donor. **g**, Viability and attH1 integration efficiency of superDn29−dCas9, delivered as mRNA, in primary T cells using standard 4.8-kb donor and sgRNA plasmids. Bars and error bars represent the mean ± s.d. of *n* = 2 biological replicates, each originating from a different blood donor. **h**, T cell viability four days after electroporation with all Dn29 variants ± dCas9 fusion, 5.8-kb CAR donor plasmid, and sgRNA plasmid. Samples correspond to those in Fig. 6h. **i**, Example gating strategy for cancer target-cell-killing assay. Nalm6 target cells are identified by CD19 expression and CAR-T cells are identified by tNGFR expression. **j**, Alignment of attH1-like pseudosites in human, marmoset, rhesus monkey, cynomolgus monkey, mouse and a sequence logo of the top 100 WT Dn29 pseudosites in HEK293FTs. **k**, Schematic of plasmid recombination assay for testing attH1-like pseudosites in HEK293FTs. **l**, Plasmid recombination efficiency between attP and each pseudosite, using Dn29 and goldDn29, in HEK293FTs. For the mouse pseudosite, the cognate attP plasmid is modified to contain the matching TA dinucleotide core sequence. Bars and error bars represent the mean ± s.d. of *n* = 3 biological replicates.

Site-specific transgene insertions show great promise in immune cell engineering, enabling integration of functional cargos such as chimeric antigen receptors (CARs) and additional immune regulators for therapeutic applications. Although primary T cells can be sensitive to high doses of nucleic acid delivery (Extended Data Fig. 9a), we developed multiple effective strategies for LSR-mediated integration. We first explored viral delivery strategies, electroporating T cells with effector mRNA and synthesized sgRNAs and delivering donor templates via single-stranded AAV (ssAAV) or self-complementary AAV (scAAV) (Fig. 6g and Extended Data Fig. 9b−d). LSR−dCas9 fusions achieved up to 17% AAV integration efficiency, which maintained high cell viability even at the highest doses. The scAAV donor yielded 2.3-fold to 3.3-fold higher integration rates compared to the ssAAV donor, likely due to double-stranded DNA requirements for LSR-mediated integration. Sequencing confirmed that more than 98% of integrations were genuine LSR-mediated events rather than non-specific AAV capture (Extended Data Fig. 9e). Because LSR-mediated integration of linear AAV genomes creates DSBs requiring cellular repair mechanisms to rejoin the inverted terminal repeat (ITR) ends, future applications could benefit from circular AAV donor templates to eliminate this repair requirement^60^.

To expand cargo capacity beyond AAV genomes and circumvent DSB repair requirements, we optimized the electroporation protocol for efficient plasmid integration (up to 14.8%) while balancing reductions in cell viability across variants (Extended Data Fig. 9f,g and Methods). These findings suggest that in more sensitive cell types, the most active recombinases (superDn29) may exhibit increased toxicity, whereas the dCas9-fused recombinases showed minimal toxicity. We integrated a 5.8-kb plasmid expressing a CD19-targeted CAR into primary T cells at up to 11% efficiency, which demonstrated robust cytotoxicity against Nalm6 cells in an in vitro cancer target cell-killing assay (Fig. 6h,i and Extended Data Fig. 9h,i). This delivery format versatility provides flexibility for different applications and manufacturing contexts, strongly supporting further development of this approach for engineering primary T cells for therapeutic applications.

Finally, we investigated the cross-reactivity of our engineered LSRs across model organisms, including mice and various non-human primates. An attH1-like sequence is present in the *NEBL* intron in marmosets, rhesus monkeys and cynomolgus monkeys, with 1−2 point mutations compared to attH1, and is located intergenically in the mouse X chromosome with six point mutations. Dn29 and goldDn29 could robustly recombine attP with the model organism pseudosites in a plasmid recombination assay in HEK293FT cells (Extended Data Fig. 9j−l), enabling future advancement in preclinical animal studies that bridge the gap between laboratory research and human clinical trials.

## Discussion

In this study, we combine directed evolution, protein engineering and machine learning models to engineer DNA recombinases to efficiently and specifically insert large genetic cargos directly into the human genome, overcoming the need to pre-install attachment site landing pads. As a proof of concept, we integrated into a single genomic locus using optimized Dn29 LSRs, achieving 13-fold to 17-fold improvements in insertion efficiency. Combining mutants with dCas9 fusions and optimized donor sequences (e-attP and sgRNA target sites) yielded recombinases with 40−53% efficiency and 90−97% genome-wide specificity for an endogenous locus.

Our engineering efforts provide numerous mechanistic insights into LSR function during genome integration. Notably, our dCas9 fusion experiments demonstrate that improved genome search and DNA binding are crucial areas for increased integration efficiency. To further interrogate DNA binding, we employed structural modeling and attachment site screening to identify specific protein and DNA regions critical for target recognition. Directed evolution revealed a general tradeoff between efficiency-improving and specificity-improving mutations for Dn29, which we overcame by strategically pairing mutations across distinct LSR domains and increasing the protein−DNA interface through fusions with DNA-binding proteins.

Our current system incorporates CRISPR components, which include both protein (dCas9) and RNA (sgRNA) elements. Although this design improves efficiency by seven-fold and specificity by five-fold, it also increases the overall size of the system and introduces an additional RNA component, which increases the manufacturing and formulation complexity. In future iterations, these CRISPR components could be replaced with smaller, protein-only DNA-binding domains, such as zinc fingers^22^. Such modifications would preserve the delivery advantages of these compact recombinases and maintain a streamlined system of a single protein and single DNA donor.

Further studies of LSR safety should be conducted to advance the translational potential of these tools. Many off-target sites exhibit minimal sequence similarity to the target site, complicating off-target locus prediction. Our most highly active variant (superDn29) also exhibited higher rates of indels or chromosomal rearrangements, and lower T cell viability, than the more balanced goldDn29. LSR−dCas9 fusions also exhibited reduced efficiency in cell-cycle-arrested cells, suggesting the need for further studies to understand potential cell cycle dependence or improve the nuclear delivery of larger fusion constructs. Finally, improved methods for off-target prediction, detection and validation will be needed to assess and overcome these challenges.

During the preparation of this paper, independent efforts to engineer the Bxb1 LSR were reported^39,61–63^. These studies focused on improving Bxb1 targeting of its natural attB site to enhance integration rates into landing pads or retarget Bxb1 to endogenous sites. However, these approaches require pre-installation of the landing pad or concurrent delivery of multiple Bxb1 variants, increasing delivery complexity and increasing the space of potential off-target sites. By contrast, our study presents multiple orthogonal engineering strategies to enhance the ability of an LSR to recognize and integrate at endogenous genomic sequences. We further demonstrate the generalizability of these approaches beyond Dn29 to Nm60, improving on-target genomic insertion efficiency to 73%.

These advancements have use across diverse research and therapeutic applications of LSRs. Current research uses lentiviral engineering of cell lines and single-copy installation of pooled libraries, which can lead to unpredictable effects on gene expression, potential insertional mutagenesis and silencing of transgenes^64^. Our engineered recombinases overcome these limitations by targeting a defined integration locus at high efficiencies, which are essential for large-scale and uniform functional genomics studies. In hESCs, we demonstrate that 95% of cells have single-copy, on-target insertions, enabling the generation of homogenous bulk cell populations without single-clone selection. Furthermore, we previously demonstrated the use of LSRs for virus-free library screening in landing pad cell lines^12^. We extend this capability, showing the feasibility of integrating RNA or protein libraries directly into an endogenous human genomic locus. In hESCs, these integrations occur at copy numbers similar to low multiplicity of infection (MOI) lentivirus (MOI = 0.1), well within the standard guidelines for approximating one integrant per cell^65^.

In the therapeutic space, this approach offers advantages over prevailing CRISPR-based gene therapies, which require a guide RNA to target a distinct disease-causing mutation. By contrast, multi-kilobase insertions enable replacement of entire corrective open reading frames, providing a ‘one-size-fits-all’ approach for correcting genetic diseases with mutational heterogeneity across patient populations. Additionally, these corrective transgenes can include critical non-coding regulatory elements for enhanced control of gene expression. Furthermore, Dn29 can cross-reactively integrate into attH1-like sequences in diverse model organisms, an important consideration for future Investigational New Drug (IND)-enabling studies.

The strategies outlined in this work can be adapted to target diverse genomic loci beyond Dn29 attH1. To target a different locus, such as validated genomic safe harbors such as *AAVS1* or therapeutic targets such as *TRAC*, LSRs can be mined from the thousands of naturally occurring orthologs to find a recombinase with a closer match to the desired target sequence. These candidate LSRs can then be subjected to our joint optimization approach, combining directed evolution, machine learning predictions and DNA-binding protein fusions to enhance both efficiency and specificity.

## Methods

### Ethics statement

Our research complies with relevant ethical regulations. Experiments using hESC lines were performed under an allowance granted by the Arc Institute Stem Cell Research Oversight Committee.

### Cell lines and culture

Experiments were conducted in HEK293FT cells (Thermo Fisher Scientific, R70007, female), H1 hESCs (WiCell Research Institute, male), WTC-11 iPSCs (Coriell Institute for Medical Research, GM25256, male) and primary human T cells (STEMCELL Technologies, 200-0092) from deidentified healthy donors. HEK293FT cells were cultured in DMEM with 10% FBS (Gibco) and 1× penicillin−streptomycin (Thermo Fisher Scientific) and dissociated using TrypLE Express (Gibco). H1 hESCs and WTC-11 iPSCs were maintained in mTeSR Plus (STEMCELL Technologies) supplemented with 1× antibiotic-antimycotic (Thermo Fisher Scientific) and cultured on Cultrex (Bio-Techne) or Matrigel (Corning) coated plates. For routine passaging, hESCs and iPSCs were dissociated with ReLeSR (STEMCELL Technologies). For 96-well plating prior to transfections, single-cell dissociation was performed using Accutase (STEMCELL Technologies). hESCs and iPSCs were supplemented with 10 µM ROCK inhibitor for 24 hours after dissociation. Primary human T cells were cultured in complete X-VIVO 15 (cXVIVO 15) (Lonza Bioscience, 04-418Q), which consists of 5% FCS (R&D Systems, M19187), 5 ng μl^−1^ IL-7 and 5 ng μl^−1^ IL-15. For the cell cycle arrest experiment, HEK293FT cells were treated with 5 μM aphidicolin at the time of transfection. HEK293FT, WTC-11 and H1 cells tested negative for mycoplasma, tested monthly.

### Dn29 deep mutational scan library construction

An NNK deep mutational scanning library of the entire Dn29 coding sequence (CDS) was generated using NNK oligos and overlap extension PCRs. First, forward and reverse oligos with NNK mixed bases at each codon were designed with a melting temperature of 65 °C. Each NNK forward primer was paired with Dn29 DMS_universal_reverse that binds downstream of the CDS and each NNK reverse primer with DMS_universal_forward primer, generating amplicons flanking the mutated codon. PCR reactions contained 2.5 µl of Q5 Master Mix (New England Biolabs (NEB)), 0.01 µl of Dn29 plasmid template (100 ng µl^−1^), 0.025 µl of universal primer (100 µM), 1.465 µl of water and 1 µl of unique NNK primer (2.5 µM). Cycling conditions were as follows: 98 °C for 30 seconds; 30 cycles of 98 °C for 10 seconds, 60 °C for 30 seconds and 72 °C for 1 minute; final extension of 72 °C for 2 minutes.

Upstream and downstream amplicons (2.5 µl each) were pooled and cleaned with 2 µl of ExoSAP-IT (Thermo Fisher Scientific) and 0.5 µl of DpnI (NEB), incubating at 37 °C for 30 minutes and then at 80 °C for 15 minutes. For the overlap extension PCR, 1 µl of cleaned PCR pool was mixed with 2.5 µl of Q5 2× Master Mix, 0.025 µl of each universal primer (100 µM) and 1.45 µl of water, using the same cycling conditions.

The full mutant pool was created by combining 2.5 µl of each overlap extension PCR. The full-length Dn29 fragment was gel extracted (Monarch DNA Gel Extraction Kit; NEB). The library and pEVO backbone were digested with XbaI and HindIII-HF (NEB). Ligation used 100 ng of total DNA (3:1 molar ratio of library to backbone), 2 µl of T4 ligase (NEB), 4 µl of 10× T4 ligase buffer (NEB) and water to 40 µl. The reaction was split into two 20-µl reactions, ligated for 30 minutes at room temperature, inactivated at 65 °C for 10 minutes and purified (Clean and Concentrator-5 Kit; Zymo Research).

The ligation product was electroporated into XL-1 Blue cells (Agilent Technologies) according to the manufacturer’s instructions, recovered for 1 hour at 37 °C in 1 ml of SOC medium and plated onto four 245-mm × 245-mm BioAssay dishes. Approximately 1 million colonies were obtained. Plasmids were purified using a NucleoBond Xtra Midi EF Kit (Macherey Nagel) and sequenced with an Illumina NextSeq 2000 600-cycle P1 Kit (Supplementary Fig. 1c) using the NextSeq 1000/2000 Control Software Suite version 1.7.1.

### Substrate-linked directed evolution

For library transformation, induction and growth: 4 µl of pEVO plasmid library was electroporated into 50 µl of XL-1 Blue competent cells (Agilent Technologies), recovered in 1 ml of SOC medium (37 °C, 1 hour) and then seeded into 100 ml of LB medium with carbenicillin and L-arabinose (10 µg ml^−1^ or 0 µg ml^−1^). Cultures were grown overnight at 37 °C. Library coverage (>1 million colonies) was confirmed by plating serial dilutions. Plasmids were extracted using a Qiagen Plasmid Midi Kit (0.3 g of wet bacteria pellet per column).

Selection of active variants: 500 ng of plasmid was digested with NdeI (NEB) to eliminate inactive variants. Active variants were amplified using 25 µl of 2× Platinum SuperFi II Master Mix (Thermo Fisher Scientific), 19 µl of water, 2 µl each of SLiDE_recovery_forward and SLiDE_recovery_reverse primers (10 µM) and 2 µl of NdeI-digested material. PCR conditions were as follows: 98 °C for 30 seconds; 30 cycles of 98 °C for 10 seconds, 52 °C for 10 seconds, 72 °C for 55 seconds; final extension at 72 °C for 5 minutes. The correct size band was gel extracted (Monarch DNA Gel Extraction Kit).

Cloning for next evolution cycle: Amplified active variants and pEVO backbone were digested with XbaI and HindIII-HF (NEB) at 37 °C for 30 minutes and then heat inactivated at 80 °C for 20 minutes. Digested variants were purified using DNA Clean and Concentrator-5 (Zymo Research) and backbone with DNA Clean and Concentrator-25 (Zymo Research). Five ligation reactions (20 µl each) were set up using 100 ng of DNA (3:1 ratio of library to backbone) and T4 ligase (NEB). Ligation occurred at room temperature for 30 minutes, followed by heat inactivation at 65 °C for 10 minutes. Pooled reactions were purified (DNA Clean and Concentrator-5 Kit), eluted in 6 µl of water and electroporated into XL-1 Blue cells to start the next evolution cycle.

### DNA shuffling and fragment reassembly

Shuffling the active variants between rounds of cycling involved a uridine exchange PCR to partially exchange thymidines for uridine, USER Enzyme fragmentation at uridine sites, primerless PCR fragment reassembly and PCR for full-length gene recovery.

Uridine exchange PCR: Fragment size and yield was optimized by modifying dUTP/dTTP ratio, with the optimal ratio being 3/7. PCR mixture: 5 µl of 10× Thermopol Buffer, 1 µl of 10 mM dNTPs, 1 µl each of SLiDE_recovery_forward and SLiDE_recovery_reverse primers (10 µM), 1 µl of plasmid library, 1 µl of Taq Polymerase and 40 µl of water. Cycling conditions were as follows: 95 °C for 30 seconds; 30 cycles of 95 °C for 20 seconds, 60 °C for 30 seconds, 68 °C for 1 minute per kilobase; final extension at 68 °C for 5 minutes. Full-length gene band was gel extracted (Monarch Gel Extraction Kit).

USER Enzyme digestion: 500-ng aliquots were digested with 2 µl of USER Enzyme (NEB) at 37 °C for 3 hours. Gel electrophoresis confirmed fragment distribution (100−1,000 bp).

Fragment reassembly: Fragments were purified (DNA Clean and Concentrator-5) and reassembled in a primerless PCR reaction using the following conditions: 25 µl of purified fragments and 25 µl of 2× Q5 High-Fidelity Master Mix (NEB). Cycling conditions were as follows: 98 °C for 30 seconds; 30−50 cycles of 98 °C for 10 seconds, 30 °C for 30 seconds (+1 °C per cycle), 72 °C for 1 minute (+4 seconds per cycle); final extension at 72 °C for 10 minutes. A final PCR was performed to recover only the full-length Dn29 CDS for further rounds of directed evolution. The following conditions were used for full-length gene recovery: PCR mixture: 25 µl of Platinum SuperFi II 2× Master Mix (Thermo Fisher Scientific), 10 µl of reassembled fragments, 2 µl each of DMS_universal_forward and DMS_universal_reverse primers (10 µM) and 11 µl of water. Cycling conditions were as follows: 98 °C for 30 seconds; 35 cycles of 98 °C for 10 seconds, 60 °C for 10 seconds, 72 °C for 55 seconds; final extension at 72 °C for 5 minutes.

The gel-extracted, shuffled and reassembled genes were cloned into the plasmid backbone using XbaI and HindIII digest and T4 ligation as previously described.

### Variant library NGS and analysis

Six primer sets (DMS_NGS primers; Supplementary Table 8) were designed to amplify approximately 260-bp segments of the Dn29 CDS with Illumina adapter overhangs. Two rounds of PCR were performed to add P5/P7 adapters and i5/i7 indexes (FLAP2 primers). Amplicons were cleaned with AMPure XP beads (Beckman Coulter) between PCR rounds and after the final PCR. Amplicons were pooled in equimolar ratios, quantified using Qubit dsDNA High Sensitivity Kit (Thermo Fisher Scientific) and sequenced on an Illumina NextSeq 2000 (600-cycle kit). Full overlap between read 1 and read 2 was ensured for higher confidence in mutation calling.

Paired-end reads were merged using BBMerge (version 39.06) and analyzed with a custom Python script. The script converted Phred quality scores to error probabilities using the formula $P=1{0}^{(Q/-10)}$, where *P* is the probability of error and *Q* is the Phred quality score. Reads with a summed error probability greater than 0.5 or containing frameshifts were filtered out. Nucleotide and amino acid mutations at each position were then counted and plotted. Enrichment for each amino acid (AA) between the input and output libraries was calculated using the following formula: ((%AA_output_) / (1 − %AA_output_)) / ((%AA_input_) / (1 − %AA_input_)). To distinguish library construction-based dropouts from selection-based dropouts in the enrichment heatmaps, any amino acids with zero reads in the output library were assigned a single read.

### Nanopore sequencing and analysis

Variants were cloned into a vector containing a 100-nucleotide (nt) random unique molecular identifier (UMI) barcode with a BHVD repeat pattern. The plasmid library was linearized by Eco105I digestion. Nanopore libraries were prepared using a barcoded nanopore sequencing kit (SQK-NBD114.24) with 1 µg of linearized plasmid library and sequenced on a MinION flow cell (R10.4.1) for 72 hours using MinKnow UI control software version 6.5.15.

Sequencing reads were filtered using nanoq (version 0.9.0) with settings –*min_len 4500–max_len 5500–min-qual 10* (that is, minimum *q* score of 10, a minimum read length equivalent to 90% of the expected read length and a maximum read length equivalent to 110% of the expected read length). The UMI sequence was extracted using Cutadapt (version 1.18) with settings *–g* *‘GGCGGTCACCATCACCACCACCACGCTACACG;max_error_rate* = *0.2…ACTGTAC;max_error_rate* = *0.2’–trimmed-only–revcomp–minimum_length 95*. All UMI sequences were trimmed to 95 nt using seqkit (version 1.3-r106) with the command *seqkit subseq -r 1:95*.

Reads were clustered by UMI with mmseqs easy-linclust (version 14.7e284) with setting *–min-seq-id 0.5*. For each UMI cluster bin with at least 15 reads, a representative cluster sequence was generated by using usearch (version 11) with settings *-cluster_fast -id 0.75 -strand both -sizeout -centroids* and taking the first representative sequence of the output^66^. A final consensus sequence was generated by one round of polishing with Medaka (version 1.9.1) with settings *-m r1041_e82_260bps_hac_g632*. Counts for each unique variant were determined by tallying the total consensus sequences.

### Cloning variant library into a mammalian expression vector

Primers (DE_mammalian_forward and DE_mammalian_reverse) were designed to amplify the Dn29 CDS from the active variant PCR library, adding overhangs for Esp3I-compatible Golden Gate cloning. PCR conditions were as follows: 25 µl of 2× Platinum SuperFi II Master Mix (Thermo Fisher Scientific), 19 µl of water, 2 µl of purified active variant library and 2 µl each of primer. Cycling conditions were as follows: 98 °C for 60 seconds; 30 cycles of 98 °C for 10 seconds, 60 °C for 10 seconds, 72 °C for 55 seconds; final extension at 72 °C for 5 minutes. The product was purified (DNA Clean and Concentrator-5) and quantified by NanoDrop (Thermo Fisher).

A mammalian expression vector was designed with the EF1α promoter upstream of an Esp3I Golden Gate landing pad, used as the destination for the protein variant library. The landing pad was followed by a T2A self-cleaving peptide sequence and an enhanced green fluorescent protein (EGFP) CDS.

Golden Gate reaction mixture: 75 ng of mammalian expression vector, amplified variant library (3:1 molar ratio to vector), 1 µl of T4 DNA Ligase Buffer (NEB), 0.5 µl of T4 DNA Ligase (NEB), 0.5 µl of Esp3I (Thermo Fisher Scientific) and up to 10 µl of nuclease-free water. Cycling conditions were as follows: 35 cycles of 37 °C for 1 minute, 16 °C for 1 minute; 37 °C for 30 minutes; 80 °C for 20 minutes. Five Golden Gate reactions were performed, pooled and purified (DNA Clean and Concentrator-5). The library was transformed into Mach1 *Escherichia coli* and plated for overnight growth. Random colonies were picked, grown in 4 ml of TB-Carbenicillin and miniprepped (NucleoSpin Plasmid Transfection Grade Mini Kit; Machery-Nagel).

### Transfection of HEK293FT cells for assessing genomic integration

One day before transfection, 12,000−18,000 HEK293FT cells were plated per well of a 96-well plate, aiming for 60–80% confluency at the time of transfection.

Standard LSR + donor transfection: for transfections containing an LSR effector plasmid and a donor plasmid, each well was transfected with 725 ng of DNA, containing a 5:1 molar ratio of donor plasmid to effector plasmid, using 0.5 µl of Lipofectamine 2000 (Thermo Fisher Scientific) per well.

Standard LSR-dCas9 + donor + guide transfection: LSR−dCas9 effector plasmid, donor plasmid and guide plasmid were transfected with 725 ng of total DNA, containing a 5:1:1 molar ratio of donor:effector:guide plasmid with 0.5 µl of Lipofectamine 2000 per well, unless specified otherwise in the figure legends.

Modified transfection conditions: Experiments shown in Fig. 3d,e and Extended Data Fig. 5b were transfected with 375 ng of effector plasmid, 100 ng of sgRNA plasmid and 250 ng of donor plasmid using Lipofectamine 2000. Experiments shown in Figs. 3g, 5 and 6 used a consolidated plasmid expressing both the effector and guide RNA. In HEK293FT experiments, this consolidated plasmid was transfected at a 5:1 ratio of donor:effector/guide plasmid with 0.585 µl of Lipofectamine 2000 per well. For transfections containing two gRNA plasmids (Fig. 3k and Extended Data Fig. 5h), each well was transfected with 375 ng of effector plasmid, 75 ng each of gRNA plasmid and 250 ng of donor plasmid.

The cells were incubated and monitored for 3 days for mCherry (donor plasmid) and GFP (effector plasmid) expression. Cells were then harvested for flow cytometry (Attune NxT Flow Cytometer; Thermo Fisher Scientific) or genomic DNA extraction for downstream analyses.

### Cell harvest, ddPCR, qPCR and flow cytometry

Three days after transfection, cells were trypsinized with 50 µl of TrypLE (Gibco) for 10 minutes and then quenched with 50 µl of Stain Buffer (BD Biosciences). The 100-µl cell suspension was split into two 50-µl aliquots in U-bottom 96-well plates and centrifuged (300*g*, 5 minutes), and the supernatant was aspirated. One plate was resuspended in 200 µl of Stain Buffer and analyzed with an Attune NxT Flow Cytometer with autosampler (Thermo Fisher Scientific).

The other plate was resuspended in 50 µl of QuickExtract DNA Solution (Biosearch Technologies), vortexed for 15 seconds and thermocycled: 65 °C for 15 minutes, 68 °C for 15 minutes, 98 °C for 10 minutes. DNA was cleaned with 0.9× AMPure XP (Beckman Coulter) beads.

To assess integration efficiency and specificity, qPCR/ddPCR primers and probes were designed to span the left integration junction of attH1 and attH3, using a constant primer that binds to the donor plasmid sequence (ddPCR_donor_reverse_1), a genome binding primer near the pseudosite (ddPCR_attH1_forward_1, ddPCR_attH3_forward) and a FAM probe within the amplicon (ddPCR_attH1_probe_1, ddPCR_attH3_probe). For attH1, a second set of primers/probes was designed to target the right junction to verify measurement accuracy (ddPCR_attH1_2 primers/probe). Genomic reference primers and probes located nearby each attachment site were designed to measure pseudosite copy number for efficiency percentage calculations.

ddPCR reaction mix (22 µl total): 11 µl of ddPCR Supermix for Probes (no dUTP) (Bio-Rad), 1.98 µl of each primer (10 µM), 0.55 µl of each probe (10 µM), 1.65 µl of cleaned gDNA, 0.22 µl of SacI-HF (NEB) and water to volume. Each reaction contained primers and probes for the target site (FAM probe) and a nearby reference locus (HEX probe). Reactions were run on a QX200 AutoDG Droplet Digital PCR System (Bio-Rad) using Bio-Rad QX Manager Software version 2.1.0, and data were analyzed and visualized using Microsoft Excel (version 16.89.1) and GraphPad Prism (version 10.3.0). For off-target detection or low-concentration samples, primers were increased to 20 µM and volume halved, and gDNA volume was increased to 4.95 µl.

qPCR reaction mix (40 µl total): 1 µl of each primer, 0.8 µl of each probe, 20 µl of TaqMan Fast Advanced Master Mix (Thermo Fisher Scientific), 2.4 µl of genomic DNA and 12 µl of water. The master mix was split into three 10-µl technical replicates in a 384-well plate and run on a LightCycler 480 (Roche) using LightCycler 480 software version 1.5.1.62. Primer pairs for ddPCR and qPCR are provided in Supplementary Table 8.

### Three-plasmid recombination assay in HEK293FT cells

A fluorescent reporter assay was used to assess episomal plasmid recombination in HEK293FT cells. One day before transfection, 12,000−18,000 HEK293FT cells were plated per well of a 96-well plate, aiming for 60−80% confluency at the time of transfection. Three plasmids at a 1:1:1 molar ratio were transfected into the cells using Lipofectamine 2000: (1) 200 ng of the effector plasmid expressing the Dn29 variants and GFP; (2) 50.5 ng of the donor plasmid containing the attP attachment sequence and mCherry; and (3) 70.6 ng of the acceptor plasmid containing an EF1α promoter and the cognate attB attachment sequence. Upon recombination of the two attachment sequences, the EF1α promoter will drive expression of the mCherry CDS, which is read out by flow cytometry (Extended Data Fig. 4d). To assess the excision reaction, the attP in the donor plasmid is replaced with the left post-recombination attachment site (attB-L:attP-R), called attL, and the attB is replaced with the right post-recombination attachment site (attP-L:attB-R), called attR. To assess attP recombination with model organism pseudosites, the attB sequence is replaced with the pseudosite sequences. Mismatching LSR (Bxb1) controls with each donor and acceptor plasmid is used to correct for the leaky mCherry background expression, defining the flow cytometry gating boundaries. Three days after transfection, the cells were trypsinized with 50 µl of TrypLE (Gibco) for 10 minutes, quenched with 50 µl of Stain Buffer, transferred to U-bottom 96-well plates and centrifuged (300*g*, 5 minutes), and then the supernatant was aspirated. Plates were resuspended in 200 µl of Stain Buffer and analyzed with an Attune NxT Flow Cytometer with autosampler.

### Site-directed mutagenesis for combinatorial mutant cloning

Site-directed mutagenesis (SDM) primers were designed using the script from Bi et al.^67^, selecting primers with melting temperature closest to 65 °C. For each mutation, a forward and reverse primer were generated, each containing the desired mutation at the center. PCR reactions were set up combining forward SDM primer with DMS_universal_reverse primer or reverse SDM primer with DMS_universal_reverse primer. The PCR mixture (12.5 µl total) contained 6.25 µl of Platinum SuperFi II Master Mix, 0.5 µl each of primer (10 µM), 1 µl of plasmid template DNA (1 ng µl^−1^) and water to volume. PCR was run using the standard Platinum SuperFi II Master Mix protocol with annealing temperature at 65 °C. Products were cleaned with 0.5× AMPure XP beads. For Gibson assembly, 1 µl each of cleaned PCR product, 5 µl of Gibson master mix and 3 µl of water were incubated at 50 °C for 15 minutes and then transformed into Mach1 *E. coli* and plated. For simultaneous cloning of two or more mutations, universal primers were replaced with other mutationsʼ forward and reverse primers. Two mutations required a two-piece Gibson assembly, three mutations required a three-piece assembly and so forth.

### Genome-wide integration site mapping

A Tn5 tagmentation and PCR amplification-based assay was used to unbiasedly measure the relative efficiency of all integration sites, as described in Durrant et al.^12^. In brief, extracted genomic DNA is tagmented with Tn5 transposase to randomly add adaptors throughout the genome. Then, two nested PCRs are performed, with primers that bind the donor plasmid and the Tn5 adaptor to amplify the donor−genome junction and add Illumina sequencing adaptors. UMIs on the donor plasmid enable counting of the relative frequencies of integration events at each genomic locus.

HEK293FT cells were transfected as previously described, with a non-matching LSR (Bxb1) plasmid replacing the effector plasmid as a control for donor plasmid dilution. Cells were cultured for 2−3 weeks, passaging and analyzing by flow cytometry every 2−3 days at 80% confluency, until the non-matching LSR control was less than 1% mCherry^+^, indicating that the plasmid had nearly completely diluted out. Genomic DNA was extracted using a Quick-DNA Miniprep Plus Kit (Zymo Research), quantified by Qubit HS dsDNA Assay (Thermo Fisher Scientific), and 1 µg of gDNA per sample was DpnI digested (NEB) to remove residual donor plasmid.

Tn5 transposase was purified following the Picelli et al.^68^ protocol. Tn5 adaptors were prepared by annealing top and bottom oligos (100 μM each) at 95 °C for 2 minutes, followed by slow cooling to 25 °C over 1 hour. The transpososome was assembled by combining 85.7 μl of purified Tn5 with 14.3 μl of pre-annealed oligos and incubating at room temperature for 1 hour. Tagmentation reactions contained 150 ng of gDNA, 4 μl of 5× TAPS-DMF, 1.5 μl of transpososome and water to 20 μl total volume. Samples were mixed thoroughly and incubated at 55 °C for 20 minutes. Reactions were placed on ice and purified with Zymo DNA Clean and Concentrate Kit according to the manufacturer’s protocol, eluting in 11 μl of nuclease-free water. Sample quality was confirmed by Bioanalyzer to verify fragmentation of approximately 1.5−2.5 kb.

For round 1 PCR, each reaction contained 12.5 μl of 2× SuperFi II Master Mix, 1.5 μl of TMAC (0.5 M), 0.5 μl of outer nest donor-specific primer (PR_N165, 10 μM), 0.25 μl of outer nest i5 primer (PR_N163, 10 μM), 1.25 μl of DMSO and 9 μl of tagmented DNA. Cycling conditions were as follows: 98 °C for 2 minutes; 12 cycles of 98 °C for 10 seconds, 68 °C for 10 seconds, 72 °C for 90 seconds; followed by 72 °C for 5 minutes. Products were purified using 0.9× Agencourt AMPure XP SPRI beads and eluted in 11 μl of water.

For round 2 PCR, each reaction contained 25 μl of 2× SuperFi Master Mix, 3 μl of TMAC (0.5 M), 2.5 μl of DMSO, 2.5 μl of i5 primer (PR_N149, 10 μM), 5 μl of i7 donor-specific primer (PR_N184-PR_N204, 10 μM), 2 μl of water and 10 μl of purified round 1 PCR product. Cycling conditions were as follows: 98 °C for 2 minutes; 18−20 cycles of 98 °C for 10 seconds, 68 °C for 10 seconds, 72 °C for 90 seconds; followed by 72 °C for 5 minutes.

For size selection, approximately 40 μl of round 2 PCR product was loaded on a 2% agarose gel, and the smear between 300 bp and 800 bp was excised. DNA was extracted using the Monarch Gel Extraction Kit according to the manufacturer’s protocol. Purified libraries were quantified using a Qubit dsDNA HS Assay Kit (Thermo Fisher Scientific) and pooled at equimolar ratios. Final library quality and molarity were assessed using a KAPA Library Quantification Kit (Roche). Pooled libraries were sequenced on a NextSeq 2000 or Illumina MiSeq with 2 × 300-bp paired-end reads, using Nextseq 1000/2000 Control Software Suite version 1.7.1 or MiSeq Control Software version 4.1.0 and Illumina BaseSpace Software version 7.38.0. Raw sequencing data were processed using a custom bioinformatics pipeline as described in Durrant et al.^12^.

To reduce occurrence of index hopping, unique dual i7 and i5 barcodes were used for the attH1 targeted samples in Fig. 3. To directly compare specificity of samples with different numbers of measured integration events, samples were downsampled to the same total UMI count.

### LSR−dCas9 and gRNA plasmid design and cloning

Fusion proteins consisting of a catalytically dead Cas9 fused to an LSR and a P2A−GFP were constructed by Gibson assembly into a pUC19-derived plasmid containing the EF1α promoter and a SV40 poly(A) tail. Variable flexible linkers, including (GGS)_8_, (GGGGS)_6_ XTEN16, XTEN32-(GGSS)_2_ and XTEN48-(GGSS)_2_, were used to link the dCas9 and LSR. Spacers targeting loci proximal to the LSR integration site and non-targeting controls were cloned into an sgRNA-expressing plasmid via oligo ligation and Golden Gate cloning. Spacer selection was based on PAM sequence and pseudosite proximity.

### Designing and cloning the attP library

Two plasmid libraries (attP-L and attP-R) were constructed to determine nucleotide preference within the attP, with each 26-bp half-site mutagenized separately. Integrated DNA Technologies (IDT)-synthesized oligo pools contained 79% WT base and 7% each of the other bases at each position. Single-stranded oligo pools were subjected to second-strand synthesis. First, an oligo anneal reaction containing 2 µl of Library Oligo (100 µM), 4 µl of Klenow primer (100 µM), 3.4 µl of 10× STE Buffer and 24.6 µl of water was heated at 95 °C for 5 minutes and then cooled to room temperature. Next, a Klenow extension reaction containing 34 µl of annealed libraries, 8 µl of water, 5 µl of 10× NEBuffer2, 2 µl of 10 mM dNTPs (NEB) and 1 µl of DNA Polymerase I, Large (Klenow) Fragment (NEB, 5,000 U ml^−1^) was incubated at 37 °C for 30 minutes, purified (DNA Clean and Concentrator-5) and eluted in 20 µl of nuclease-free water.

The purified product was cloned by Esp3I Golden Gate cloning into the donor plasmid backbone: 75 ng of pre-digested backbone, 3:1 molar ratio of attP library to backbone, 0.5 µl each of T4 DNA ligase (NEB) and Esp3I (Thermo Fisher Scientific), 1 µl of T4 DNA Ligase Buffer (NEB) and water to 10 µl were incubated at 37 °C for 1 hour, purified and eluted in 6 µl of nuclease-free water. Then, 1 µl of purified library was electroporated into Endura Electrocompetent Cells (Biosearch Technologies) at 10 μF, 600 Ω, 1,800 V, recovered in 2 ml of Lucigen Recovery Medium (37 °C, 1 hour), plated on 245-mm × 245-mm BioAssay dishes and incubated at 30 °C overnight. Final libraries were scraped, purified (Nucleobond Xtra Maxi EF Kit) and sequenced with an Illumina NextSeq 2000.

### attP library transfection, harvest and library preparation

Next, 2.2 × 10^6^ HEK293FT cells were plated on 10-cm dishes 1 day before transfection to achieve 70% confluence at transfection. Then, 24 μg of total plasmid DNA was prepared at a 5:1:1 molar ratio (attP library:LSR effector:sgRNA). DNA and 72 μl of Lipofectamine 2000 were separately mixed with 1.5 ml of OMEM, incubated for 5 minutes and then combined and incubated for 10 minutes before adding dropwise to cells. After 3 days, cells were harvested with TrypLE (Gibco), and genomic DNA was extracted using a Quick-DNA Midiprep Plus Kit (Zymo Research).

Integration events were amplified by single-step PCR with i5/i7 index-adding primers using all available genomic DNA. Biological replicates had 1-bp staggered amplicons to increase nucleotide diversity. PCR conditions were as follows: 25 μl of NEBNext High Fidelity PCR Master Mix, 2.5 μg of genomic DNA, 1.25 μl each of the attL or attR i5 or i7 primers (Supplementary Table 8) and water to 50 μl. Cycling conditions were as follows: 25 cycles of 98 °C for 10 seconds, 63 °C for 10 seconds, 72 °C for 25 seconds. PCR products were pooled and run on 2% agarose gel, and correct size bands were extracted (Monarch DNA Gel Extraction Kit). Libraries were quantified (Qubit 1× dsDNA High Sensitivity Assay; Thermo Fisher Scientific), pooled equimolar with 35% PhiX spike-in and sequenced on an Illumina NextSeq 2000 (150-bp paired-end reads).

### attP library enrichment analysis

Libraries were demultiplexed using Illumina BaseSpace automatic demultiplexing workflow. Paired-end reads were merged using BBMerge (version 39.06) and analyzed with a custom Python script. Reads were filtered for exact amplicon length and QScore ≥ 30. Next, percent abundance of each nucleotide at each attP position was calculated for input and output libraries. Enrichment scores were computed using the following equation: $r=\frac{{A}/1-A}{B/1-B}$, where *A* and *B* represent the read counts for selected nucleotides in output and input libraries, respectively, normalized to the total number of reads. Enrichment scores were converted to sequence logos, generated using Logomaker^69^ and matplotlib packages.

Unique library members recovered as integration events were assessed by generating the set of unique reads. The number of unique integration events from NGS analysis was compared to ddPCR analysis of bulk genomic DNA for validation.

Dinucleotide enrichment analysis was performed by first counting individual nucleotide frequencies at each position across all reads, followed by counting all possible dinucleotide combinations using a 2-bp sliding window at consecutive position pairs. Raw counts were normalized to total reads to calculate probabilities for both single nucleotides and dinucleotides at each position. To assess deviation from independence, observed dinucleotide probabilities were divided by the product of their constituent single-nucleotide probabilities: P(dinucleotide) / (P(nucleotide_1_) × P(nucleotide_2_)).

Enrichment scores were calculated by comparing output to input library frequencies using $r=\frac{{A}/1-A}{B/1-B}$, where *A* represents output library frequency and *B* represents input library frequency for each dinucleotide. Final values were log_2_ transformed and averaged across dinucleotide categories based on purine (R: A,G) and pyrimidine (Y: C,T) classification: RR (purine−purine), YY (pyrimidine−pyrimidine), RY (purine−pyrimidine) and YR (pyrimidine−purine).

### Stem cell transfection

H1 hESCs and WTC-11 iPSCs were cultured in mTeSR Plus medium (STEMCELL Technologies) on Cultrex-coated (Bio-Techne) or Matrigel-coated (Corning) six-well plates. Cells were routinely subcultured at a 1:12 ratio using ReLeSR Passaging Reagent (STEMCELL Technologies) every 4 days or at 70−80% confluency. Three days after splitting (60% confluency), the cells were dissociated for 10 minutes with Accutase (STEMCELL Technologies) and plated in Cultrex-coated 96-well plates at 25,000−30,000 cells per well with 10 µM ROCK inhibitor. The next day (at 70% confluency), media were changed to include 50 µM ROCK inhibitor 2 hours before transfection. Then, 3 µg of plasmid DNA containing a 1:1 molar ratio of combined effector/guide plasmid to donor plasmid in 10-µl volume was diluted in 81 µl of mTeSR Plus and thoroughly pipette mixed. Next, 9 µl of FuGENE HD Transfection Reagent (Promega) was added to the DNA/mTeSR mix, thoroughly mixed and incubated for 12 minutes. After another thorough pipette mix, 7 µl of the DNA was added dropwise to each well. The cells were incubated at 37 °C, splitting 1:2 if 90% confluency was reached. After 3 days, the cells were dissociated with Accutase and split into two V-bottom plates, one for flow cytometry and one for gDNA harvest with QuickExtract DNA Solution (Biosearch Technologies).

### HPC differentiation and surface marker staining

hESCs were differentiated into HPCs using the STEMdiff Hematopoietic Kit (STEMCELL Technologies). On day 10 of differentiation, 250 µl of non-adherent cells were collected from the supernatant using wide-bore P1000 tips and transferred to a V-bottom 96-well plate. Next, the cells were pelleted at 400*g* for 5 minutes, supernatant discarded and resuspended in 95 ml of Stain Buffer containing 1 µl of each antibody with a wide-bore pipette. The following antibodies were used: APC CD81 (BD Biosciences, 551112), APC CD147 (Thermo Fisher Scientific, A15706), Alexa Fluor 647 CD63 (BD Biosciences, 561983), APC/Cyanine7 CD34 (BioLegend, 343514) and PE CD43 (BioLegend, 343204). The cells were incubated in the dark for 20 minutes to 1 hour, washed once with Stain Buffer and flowed on the Attune Flow Cytometer (Thermo Fisher Scientific) using Attune Cytometric Software version 5.3.0 for collection.

### hESC single-cell dilution and genotyping

hESCs were diluted to one cell per 100 µl in mTeSR Plus medium supplemented with 1× CloneR (STEMCELL Technologies) and plated into two 96-well plates per sample. Cells were maintained until colonies were visible, and then wells with multiple colonies were removed. Single colonies were expanded to 24-well dishes when they covered half the surface area of the 96-well plate. At the next split, one quarter of each well was pelleted for gDNA extraction using QuickExtract DNA Solution (Biosearch Technologies). The extracted gDNA was cleaned with 0.9× AMPure XP beads and genotyped by ddPCR. Primers and probes were designed to target the attH1 junction (ddPCR_attH1_1 set), the donor sequence (Amp_forward, Amp_reverse, Amp_probe) and a nearby genomic reference sequence. On-target zygosity was determined by the attH1/reference ratio, and total zygosity was measured by the donor/reference ratio.

### Bulk RNA-seq—cell line generation, RNA isolation and sequencing

Stem cells were transfected as previously described. Two days after transfection, cells were selected using Geneticin (Gibco) at 100 μg ml^−1^ and penicillin−streptomycin (Gibco) at 100 U ml^−1^. Cells were maintained in culture for 3 weeks until selection was complete and sufficient cell expansion was achieved for downstream applications, including cryopreservation and RNA extraction. Throughout the culture period, cell quality was monitored daily via microscopy to assess morphology and identify spontaneous differentiation events. Culture medium consisting of mTeSR Plus supplemented with penicillin−streptomycin and Geneticin was replaced daily. Upon reaching 70−80% confluency, cells were clump passaged using ReLeSR according to the manufacturer’s instructions.

If spontaneous differentiation was observed, cells were subjected to a straining protocol to remove differentiated cells and maintain pluripotent populations. In brief, media were aspirated, and four drops of ReLeSR were added to each well and incubated for 10 minutes. Cells were gently dislodged by pipetting or tapping the side of the culture dish to release cell clumps. The cell suspension was passed through a 40-μm cell strainer placed on a 50-ml Falcon tube and rinsed with 6 ml of PBS. The strainer was then inverted onto a fresh Falcon tube, and clumps were collected with 3 ml of culture medium before replating into six-well plates.

For RNA-seq, cells from one well of a six-well plate were harvested by adding 1 ml of TRIzol reagent. The lysate was mixed by pipetting until a homogeneous viscosity was achieved and stored at −80 °C until RNA extraction.

For RNA extraction, 200 μl of chloroform was added, followed by vigorous shaking for 15 seconds and incubation at room temperature for 10 minutes. Samples were centrifuged at 12,000*g* for 15 minutes at 4 °C, resulting in phase separation. The upper aqueous phase containing RNA was carefully transferred to a fresh tube, and 0.5 ml of isopropanol was added and mixed. After a 5−10-minute incubation at room temperature, samples were centrifuged at 12,000*g* for 10 minutes at 4 °C to precipitate RNA. The supernatant was removed, and the RNA pellet was washed with 1 ml of 75% ethanol, mixed and centrifuged at 7,500*g* for 5 minutes at 4 °C. The RNA pellet was air dried for 5−10 minutes before resuspension. The extracted RNA was analyzed on a High Sensitivity RNA ScreenTape (Agilent Technologies) to measure the RNA integrity number (RIN) score, which was higher than 9 for all samples.

mRNA enrichment was performed using the Roche/KAPA mRNA HyperPrep Kit according to the manufacturer’s protocol. After mRNA enrichment, sequencing libraries were prepared using the HyperPrep Library Preparation Kit according to the manufacturer’s instructions. Final libraries were sequenced on an Illumina NovaSeq X at a depth of at least 20 million reads per sample.

### RNA-seq data processing

Raw paired-end FASTQ files for each stem cell sample were first subjected to adapter and quality trimming using Trim Galore (version 0.6.7)^70^ with default settings, retaining reads ≥20 nt. Quality of raw and trimmed reads was assessed with FastQC (version 0.11.9)^71^ and aggregated using MultiQC (version 1.15)^72^. Trimmed reads were aligned to the GRCh38.p13 reference genome (GENCODE version 46 primary assembly, FASTA and GTF obtained from GENCODE) using STAR (version 2.7.10a)^73^ in two-pass mode. Alignment metrics and insert size distributions were evaluated with Picard (version 2.27.4)^74^, RSeQC (version 4.0.0)^75^, Qualimap (version 2.2.2)^76^, dupRadar (version 3.21)^77^ and Qualimap RNA-seq modules, with reports again aggregated by MultiQC. Concurrently, Salmon (version 1.10.0)^78^ was used to quantify transcript abundances (–validateMappings), and transcript-to-gene summarization was performed to produce gene-level count and transcripts per million (TPM) matrices. All steps were orchestrated via the nf-core/rnaseq pipeline (version 3.12.0)^79^ under Nextflow (version 24.10.0)^80^ with the Docker (version 28) profile, specifying –strandedness reverse^81^.

### Differential expression analysis

Gene-level count matrices (salmon.merged.gene_counts.tsv) were imported into R (version 4.3.1)^82^, and DESeq2 (version 1.38.1)^83^ was used for normalization and differential expression. A sample metadata table containing sample_id, condition and group_id was preprocessed so that identifiers matched the column names of the count matrix. For each stem cell line (group_id), the wild-type (‘WT’) condition was identified, and pairwise comparisons were performed between each edited condition and the corresponding wild-type condition. Differential expression was modeled in DESeq2 with the formula ‘~ condition’ (R formula syntax), meaning that gene counts were fit as a function of the experimental condition (edited or wild-type). Wald tests were used to estimate log_2_ fold changes, and *P* values were adjusted for multiple testing by the Benjamini–Hochberg (false discovery rate (FDR)) method. DEGs were defined as those with adjusted *P* < 0.05 and |log_2_ fold change | > 1.

### HEK293FT single-cell sorting and genotyping

HEK293FT cells were transfected as previously described. Eight days after transfection, cells were placed under puromycin selection (0.5 μg ml^−1^) for 10 days. On day 18, cells were trypsinized and strained through a 35-µm filter, and single mCherry^+^ cells were sorted into four 96-well plates per sample using a FACSAria Fusion (BD Biosciences). Single-cell colonies were expanded for 2 weeks until more than 50% confluent, with visual inspection to ensure single colony growth. Wells with zero or multiple colonies were excluded from analysis.

Confluent colonies were harvested with QuickExtract DNA Solution (Biosearch Technologies) and amplified in two separate PCRs: PCR 1 using primers UMI_reverse and ddPCR_attH1_forward_1, flanking the UMI and attH1 donor/genome junction, and PCR 2 using primers UMI_reverse and UMI_forward, flanking the UMI on the donor plasmid. Amplicons were sequenced via Sanger and/or NGS to determine on-target UMI count (PCR 1) and total UMI count (PCR 2), allowing calculation of on-target and off-target insertion counts per colony.

### Quantification of indels at attH1

HEK293FT cells were transfected with LSR and donor plasmids at a 1:5 ratio, as described above. After 3 days, cells were passaged into a 24-well dish for expansion. On day 5 after transfection, genomic DNA was harvested using the Zymo Quick-DNA Miniprep Plus Kit according to the manufacturer’s instructions. PCR primers with 0−5 stagger base pairs were designed to amplify the attH1 site. Each PCR reaction contained 1 μg of gDNA, 40 μl of Platinum SuperFi II Master Mix, 3.2 μl of forward primer (PR_N284−PR_N289, 10 μM), 3.2 μl of reverse primer (PR_N290−PR_N295, 10 μM) and water to 80 μl total volume. After 25 cycles under standard conditions, products were purified with 0.8× AMPure XP beads. A second PCR amplification added Illumina indexes using 1 μl of purified product, 12.5 μl of Platinum SuperFi II Master Mix, 1 μl each of uniquely indexed FLAP2 primers and 9.5 μl of water. After seven cycles, libraries were purified with 0.7× AMPure XP beads, quantified via Qubit, pooled and sequenced using Illumina chemistry.

Indel rates were calculated using Crispresso2 with the following command: CRISPResso -a CATTGGTGAATGTCTCATGTGGGTTTGAAAAGAGTGTGTATTCTGCTGTTGTTGGGTAAAGTAGTCTATACATGTCAATGATATGCTGTTGATTGATGCTGGTGTTGAATTCAACTATGTCCTTGCTGATTTTCTGCCTGCTGGATCTGTCTGAC -g GTCTATACATGTCAATGATA -r1 Read_1.fastq.gz -r2 Read_2.fastq.gz–keep_intermediate -w 20 -q 30–min_bp_quality_or_N 30–exclude_bp_from_left 10–exclude_bp_from_right 10–plot_window_size 20–ignore_substitutions. The Modified% output value represented the percentage of unintegrated cells containing indels. Background indel rates from untransfected cells were subtracted from each sample. The final percentage of cells with indels was calculated by multiplying the Modified% by the percentage of uninserted cells (1 minus the average insertion percentage determined by ddPCR).

### Cell viability assay

HEK293FT cells were plated in black-walled, clear-bottom optical plates, excluding edge wells and transfected with LSR and donor plasmids at a 1:5 ratio with four replicates per sample. Two days after transfection, cell viability was assessed using the CellTiter-Glo Assay (Promega). Cells were first refreshed with 100 μl of fresh D-10 medium and then treated with 100 μl of combined room temperature CellTiter-Glo Buffer and Substrate. Plates were orbitally shaken at 510 r.p.m. for 2 minutes on a Tecan Spark Microplate Reader and incubated for an additional 8 minutes, and then luminescence was measured with 1,000-ms integration time. Background luminescence from empty wells was subtracted from all measurements. Final viability values were normalized to control cells transfected with donor plasmid and pUC19 stuffer plasmid in place of the LSR effector plasmid.

### Phosphorylated H2AX staining and flow cytometry

HEK293FT cells were plated into 96-well plates and transfected as described above. Two days after transfection, cells were dissociated with TrypLE and transferred to a V-bottom plate. Cells were centrifuged at 300*g* for 5 minutes and washed with 200 μl of DPBS. Next, cells were centrifuged again and resuspended in 50 μl of 4% paraformaldehyde (diluted in DPBS) for fixation. Cells were incubated for 10 minutes at room temperature. After fixation, cells were washed three times and stored in PBS overnight. To permeabilize cells, samples were resuspended in 0.25% Triton-X (diluted in DPBS) and incubated for 15 minutes at room temperature in the dark. Next, cells were washed twice with DPBS and incubated in blocking buffer composed of the following: 10% goat serum (Sigma-Aldrich, G6767), 0.5% NP-40 (Sigma-Aldrich, I3021) and 5% w/v saponin (Sigma-Aldrich, 84510) diluted in DPBS. Samples were incubated in blocking buffer for 30 minutes at room temperature in the dark. After incubation, samples were centrifuged and resuspended in a 1:1,000 dilution of Alexa Fluor 647-conjugated anti-phospho histone H2A.X (Ser139) antibody (Sigma-Aldrich, cat. 05-636-AF647, clone JBW301, lot 4214083) diluted in blocking buffer. Samples were incubated for 2 hours, washed twice with DPBS and analyzed on the Attune flow cytometer.

### Quantification of translocations and genomic rearrangements

HEK293FT cells were transfected with LSR and donor plasmids at a 1:5 ratio. After 3 days, cells were passaged into a 24-well dish for expansion. On day 5 after transfection, genomic DNA was harvested using the Quick-DNA Miniprep Plus Kit. Tn5 tagmentation was performed as described above, with two reactions performed per sample.

For enrichment of translocation junctions, tagmented DNA underwent a two-step nested PCR. The first PCR combined 10.5 μl of tagmented DNA with 12.5 μl of Platinum SuperFi II Master Mix, 1 μl of outer nest primer (PR_N296 for upstream or PR_N297 for downstream of attH1) and 1 μl of PR_N163 (Tn5 adaptor binding). Reactions were amplified for 12 cycles (standard three-step protocol, 60 °C annealing, 1-minute extension), purified with 0.9× AMPure XP beads and eluted in 11 μl of water. The second nested PCR added indexes and Illumina adaptors using 10 μl of the first PCR product, 25 μl of Platinum SuperFi II Master Mix, 2.5 μl of inner primer (PR_N298−PR_N327 for upstream samples or PR_N328−PR_N356 for downstream samples), 2.5 μl of PR_N149 and 10 μl of water. After 20 cycles, products were purified with 0.9× AMpure XP beads, quantified by Qubit and pooled equimolarly. Amplicons between 300 bp and 900 bp were selected by gel extraction, quantified using the KAPA Library Quantification Kit and sequenced with Illumina chemistry for 600 cycles.

Genomic rearrangements and translocations were identified using a custom pipeline. After merging paired-end reads, the sequence between the inner primer and the attH1 dinucleotide core (‘upstream sequence’) was searched for, allowing up to three mismatches to account for sequencing errors. Reads containing the upstream sequence were processed to extract downstream portions (minimum 20-bp length), which were then aligned to both WT and donor insertion references using BWA-MEM (-a -M -k 8 -T 20)^79^. Reads were classified based on alignment quality (≥80% alignment and mapping quality (MAPQ) ≥ 20) into WT aligned, donor insertion aligned or potential translocations.

Potential translocation reads underwent further analysis by BWA alignment to the human reference genome (hg38). The resulting alignments were converted to sorted BAM files using SAMtools (version 1.22) for visualization and BED files using BEDTools (version 2.31.0) for genome browser compatibility.

Translocation events were classified into four categories: (1) close to target (within 2 kb of on-target site, reclassified as WT aligned); (2) EF1α promoter aligned (mapping to chr6 region 73,519,610−73,522,070, reclassified as donor insertion aligned); (3) non-chr10 translocations; and (4) chr10 rearrangements.

To quantify the presence of ITRs at the attH1/donor junction of AAV integrations, the same protocol was used, using the upstream bait primers. All reads containing the upstream sequence were aligned to WT and donor insertion references using BWA (version 0.7.19). All reads that did not align to these references were then aligned to the human genome. Finally, all reads that did not align to the human genome were aligned to AAV ITR sequences using Geneious Prime (version 11.0.20.1+1).

### Lentivirus production and HPC transduction

sgRNA spacers targeting cell surface markers CD81, CD147 and CD63 were cloned into the LentiGuide-Puro construct (Addgene, 52963). Lentivirus was generated using the LV-MAX Lentiviral Production Kit (Invitrogen) according to the manufacturer’s instructions and concentrated 100× with Lenti-X Concentrator (Takara). HPCs were diluted to 50,000 cells per well in 100 µl of Medium B (STEMCELL Technologies, STEMdiff Hematopoietic Kit) in a 96-well plate. Each well received 1 µl of LentiBOOST (SIRION Biotech) and 1 µl of lentivirus. Media were changed the next day. Four days after transduction, a subset of cells was stained for cell surface markers. Remaining cells were treated with 1 μg ml^−1^ puromycin for 4 days to select for transduced cells, followed by cell surface marker staining. Antibodies used for cell surface staining were as follows: APC CD81 (BD Biosciences, cat. 551112, lot 2061009, clone JS-81, 1:100 dilution); APC CD147 (Thermo Fisher Scientific, cat. A15706, lot 540242, clone 8D12, 1:100 dilution); Alexa Fluor 647 CD63 (BD Biosciences, cat. 561983, lot 2112938, clone H5C6, 1:100 dilution); APC CD63 (BioLegend, cat. 353008, lot B373947, clone H5C6, 1:100 dilution); APC/Cyanine7 CD34 (BioLegend, cat. 343514, lot B413134, clone 581, 1:100 dilution); and PE CD43 (BioLegend, cat. 343204, lot B359578, clone CD43-10G7, 1:100 dilution). All antibodies chosen are validated for flow cytometric analysis of human cells according to the manufacturer’s website.

### Generating AlphaFold3 models of Dn29 bound to attB

The full-length WT Dn29 protein sequence and minimal attB-L or attB-R sequence (attB-L: GTAGACAAGGAAGGTAATGA; attB-R: GAAATAAGTTTGATAGATAT) were input into the AlphaFold3 web server with the seed set to ‘auto’. Five models were generated for each query of Dn29 bound to an attB half-site. Outputs were manually inspected in pymol (version 3.0.2) to ensure correct orientation of Dn29 bound to the half-site, with the dinucleotide core of the DNA proximal to the NTD. One model (Dn29 × attB-R) out of the 10 generated models met this criterion and was selected for further analysis. The chosen model was compared to the *Listeria* integrase crystal structure of the LSR CTD and attP complex (PDB: 4KIS). Despite 4KIS being bound to attP instead of attB, domain-wise comparisons showed strong alignment: RMSDs were 1.341 and 1.707 for the zinc-ribbon domain and the recombinase domain, respectively (Extended Data Fig. 4b). Protein/DNA interface residues were identified with the InterfaceResidues pymol script using default settings.

### Predicting combinatorial mutations and feature importance with machine learning

The efficiency and specificity data of all Dn29 variants were split into a training and test set based on what round of experimentation they were generated in. The training set, called round 1, contained all variants from the two single-mutation validation experiments, where mutations were tested individually on top of variant 127 (Fig. 1g and Extended Data Fig. 2c−e) or variant 381 (Extended Data Fig. 2f). The testing set contained all higher-order combinations from the iterative rounds of driver mutation stacking (rounds 2−5). The efficiency (percent of integrations at attH1) was normalized to WT, and specificity (ratio of attH1/attH3 activity) was log transformed. The full amino acid sequences of the protein variants were one-hot encoded, a technique that transforms each amino acid in the sequence into a binary vector of length 21 (corresponding to the 20 standard amino acids plus a stop codon), where the position corresponding to that amino acid is set to 1 and all others are 0—this encoding is then flattened into a single vector representing the entire sequence. Activity in the training set was modeled using linear regression, ridge regression, XGBoost and CatBoost with the scikit-learn (version 1.0.2), xgboost (version 1.6.2) and catboost (version 1.2.5) Python libraries. Additional Python packages used include pandas (version 1.3.5), numpy (version 1.19.5), matplotlib (version 3.5.2), seaborn (version 1.73) and scipy (version 1.7.3).

For the ridge regression, optimal *α* was identified through minimization of the testing set *R*^2^ (*α* = 0.8 for efficiency model, *α* = 1.3 for specificity model). Hyperparameter optimizations were conducted for XGBoost and CatBoost by performing a randomized search, evaluating on negative mean squared error, using the following parameters: XGBoost: ‘n_estimators’: [100, 500, 1,000], ‘learning_rate’: [0.01, 0.05, 0.1], ‘max_depth’: [3, 5, 7], ‘subsample’: [0.5, 0.6, 0.7, 0.8, 1.0], ‘colsample_bytree’: [0.7, 0.8, 1.0]; CatBoost: ‘iterations’: [100, 200, 500, 1,000], ‘learning_rate’: [0.01, 0.05, 0.1, 0.2], ‘depth’: [4, 6, 8, 10], ‘l2_leaf_reg’: [1, 3, 5, 7, 9], ‘bagging_temperature’: [0, 1, 2, 3], ‘random_strength’: [1, 1.5, 2, 3], ‘border_count’: [32, 64, 128], ‘grow_policy’: [‘SymmetricTree’, ‘Depthwise’, ‘Lossguide’].

The following parameters were chosen for each model: XGBoost, specificity: ‘subsample’ = 0.5, ‘n_estimators’ = 1,000, ‘max_depth’ = 7, ‘learning_rate’ = 0.1, ‘colsample_bytree’ = 0.8; XGBoost, efficiency: ‘subsample’ = 0.7, ‘n_estimators’ = 100, ‘max_depth’ = 7, ‘learning_rate’ = 0.05, ‘colsample_bytree’ = 0.7; CatBoost, specificity: ‘random_strength’ = 1.5, ‘learning_rate’ = 0.1, ‘l2_leaf_reg’ = 1, ‘iterations’ = 1,000, ‘grow_policy’ = ‘Depthwise’, ‘depth’ = 4, ‘border_count’ = 128, ‘bagging_temperature’ = 1; CatBoost; efficiency: ‘random_strength’ = 1.5, ‘learning_rate’ = 0.1, ‘l2_leaf_reg’ = 7, ‘iterations’ = 500, ‘grow_policy’ = ‘Lossguide’, ‘depth’ = 4, ‘border_count’ = 32, ‘bagging_temperature = 2.

### In vitro transcription and purification of mRNA

Effector constructs were cloned into an in vitro transcription (IVT) plasmid as previously described^84^. This plasmid contained a mutated T7 promoter, 5’ untranslated region (UTR), P2A EGFP and 3’ UTR followed by a 145-bp poly(A) sequence. IVT templates were generated by PCR using primers oGX006 and oLGR009, which incorporate a poly(A) tail and correct the T7 promoter mutation. PCR reactions were performed using KAPA HiFi HotStart 2× (Roche) Master Mix with 6.25 ng of plasmid template per 25-µl reaction. The PCR protocol involved annealing at 63 °C, extending for 45 seconds per kilobase and running for 18 cycles. The reactions were purified using 0.8× volume of SPRI beads and eluted into water. The purified PCRs were analyzed by gel electrophoresis and NanoDrop to ensure correct size and determine concentration.

The IVT reactions were set up using the HiScribe T7 High-Yield RNA Synthesis Kit (NEB, E2040S), modified with full pseudo-UTP substitution using N1-Methyl-Pseudo-U (TriLink Biotechnologies, N-1081) and co-transcriptionally capped with CleanCap AG (TriLink Biotechnologies, N-7113). Each IVT reaction contained 5 mM ATP, CTP, GTP and pseudo-UTP, 4 mM CleanCAP AG, 1× Transcription Buffer, 3.75 ng µl^−1^ DNA template, 1 U µl^−1^ Murine RNAse Inhibitor (NEB, M0314L), 0.002 U µl^−1^ yeast inorganic pyrophosphatase (NEB, M2403L) and 5 U µl^−1^ T7 RNA polymerase. Reactions were incubated for 2.5 hours at 37 °C.

Next, the mRNA was purified using lithium chloride. To each reaction, 1.5× water and 1.25 × 7.5 M LiCl were added. The solution was chilled at −20 °C for 30 minutes and then spun at maximum speed (16,000*g*) for 15 minutes at 4 °C. The supernatant was discarded, and the pellet was rinsed with 70% ice-cold ethanol to remove residual salts. After another maximum speed spin for 10 minutes at 4 °C, the mRNA was resuspended in water and stored at −80 °C. The mRNA was analyzed on an Agilent TapeStation and by Qubit RNA High Sensitivity (Thermo Fisher Scientific) to ensure correct size and determine concentration.

### RNA electroporation and AAV transduction of primary human T cells

Two days before electroporation, T cells were seeded at 1 × 10^6^ fresh cells per milliliter and activated with a 1:1 bead-to-cell ratio with anti-CD3/CD28 Dynabeads (Life Technologies, 40203D). On the day of electroporation, the beads were magnetically removed, and the T cells were electroporated with 2 µg of LSR−dCas9−P2A−EGFP mRNA and 2 µg of sgRNA (Synthego) for LSR−dCas9 samples or 1 µg of LSR−P2A−EGFP mRNA for LSR samples using the Lonza P3 Primary Cell Kit. Each electroporation contained between 0.5 × 10^6^ and 1 × 10^6^ cells in 20 µl total volume and was electroporated using the 4D Nucleofector system and the DS-137 pulse code. Immediately after electroporation, 80 µl of pre-warmed culture media was added to the Nucleocuvette strip, which was then incubated at 37 °C for 15−30 minutes. Next, 2 × 10^5^ cells per condition were split into 96-well U-bottom plates in 100 µl of serum-free medium (TheraPEAK X-VIVO-15 Serum-free Hematopoietic Cell Medium, BEBP04-744Q) supplemented with 5 ng μl^−1^ IL-7 and 5 ng μl^−1^ IL-15. Cells were then transduced at an MOI of 1 × 10^5^ genome copies per cell with ssAAV or scAAV vectors of serotype 6 (AAV6) containing the e-attP sequence, attH1 sgRNA target sequence and an mCherry expression cassette, which were ordered from VectorBuilder. The next morning, cells were spun down at 300*g* for 5 minutes; the serum-free medium was removed; and cells were resuspended in 200 µl of fresh cX-VIVO. Cells were maintained and passaged as needed by the addition of cX-VIVO every 2−3 days.

### Plasmid and mRNA electroporation of primary human T cells

Peripheral blood mononuclear cells (PBMCs) from healthy human blood donors were collected under an approved institutional review board protocol by the Stanford Blood Center and used to isolate human T cells. In brief, leukoreduction chambers from processing of platelet donations were used to isolate PBMCs using density centrifugation with Ficoll (Lymphoprep; STEMCELL Technologies) within SepMate tubes (STEMCELL Technologies) according to the manufacturerʼs instructions. Next, primary human CD3^+^ T cells were isolated by negative selection using a Human CD3 T Cell Enrichment Kit (STEMCELL Technologies) according to the manufacturerʼs instructions. Isolated primary human CD3 T cells were counted using an automated cell counter (Countess; Thermo Fisher Scientific) and activated using anti-human CD3/CD28 Dynabeads (Cell Therapy Systems; Thermo Fisher Scientific) at a 1:1 ratio in X-VIVO 15 medium (Lonza) supplemented with 5% FBS (MilliporeSigma) and 50 IU ml^−1^ human IL-2 (PeproTech). T cells were activated at a 1:1 ratio of cells to Dynabeads and initially cultured in standard tissue culture incubators at approximately 1 × 10^6^ cells per milliliter of medium. After gene editing/electroporations, T cells were counted and reseeded at approximately 1 × 10^6^ cells per milliliter, with additional IL-2 and X-VIVO 15 complete media added every 2–3 days to maintain a culture density of approximately 1 × 10^6^ cells per milliliter.

Forty-eight hours after activation, Dynabeads were magnetically removed from activated T cell cultures by incubating for 2 minutes at room temperature on a magnet (EasySep Magnet; STEMCELL Technologies), and cells were counted using an automated cell counter (Countess; Thermo Fisher Scientific). For electroporations, 1−2 million T cells per editing condition were gently pelleted by centrifugation at 90*g* for 10 minutes, followed by careful aspiration of the supernatant. T cell pellets were resuspended in 20 μl per editing condition in P3 buffer (Lonza) and then mixed with prepared LSR mRNA and DNA templates. Then, 1.5 µg of LSR mRNA, 2 µg of donor plasmid, 1.5 µg of sgRNA plasmid and 20 µl of T cell suspension were mixed and aliquoted into a 96-well Nucleocuvette plate (Lonza). All plasmids were purified using the ZymoPure II Plasmid Midiprep Kit (Zymo Research). The 5.8-kb CD19 CAR-expressing plasmid contains the EF1α promoter, tNGFR EC domain (cell surface reporter), T2A, 1928z CAR and bGH poly(A). Total nucleic acid volume was limited to 5 µl. Electroporation occurred on a Gen2 Lonza 4D instrument with a 96-well plate attachment using pulse code EO-151. Immediately after electroporation, 80 μl of pre-warmed X-VIVO 15 media was added to each cuvette, and cells were rested within the cuvettes for 15 minutes in a standard 37 °C tissue culture incubator. The cells were then gently resuspended and transferred to standard 96-well round-bottom plates with 300 µl of total X-VIVO 15 complete medium with 50 IU ml^−1^ human IL-2. T cells were maintained at 0.5 × 10^6^ to 1 × 10^6^ cells per milliliter, and X-VIVO 15 complete medium with 50 IU ml^−1^ human IL-2 was refreshed every 2−3 days.

### T cell staining, flow cytometry and genomic harvesting

Three days after electroporation, 50 µl of T cells was collected for staining and flow cytometry. In brief, cells were centrifuged, washed once with 200 μl of cell staining buffer and stained with Ghost Dye Red 780 at a 1:1,000 dilution (Tonbo, 13-0865-T500) for 20 minutes in the dark at 4 °C. The cells were measured using an Attune NxT Cytometer with a 96-well autosampler (Invitrogen) and analyzed using FlowJo software (version 10.10.0) for viability, mCherry fluorescence (expressed on the AAV) and GFP fluorescence (effector expression). The remaining 150 µl of T cells in culture was centrifuged at 300*g* for 5 minutes, and the gDNA was harvested using QuickExtract DNA Solution (Biosearch Technologies) and analyzed by ddPCR as described above.

### In vitro cancer target cell-killing assays

At 13 days after non-viral gene editing, T cell editing was assessed by flow cytometry, and cells edited with goldDn29, goldDn29−dCas9 and Dn29−dCas9 were selected for the killing assay. T cells were mixed at indicated effector:target (E:T) ratios with target Nalm6 leukemia cells in 96-well plates, with four different Nalm6 conditions (16,000, 8,000, 4,000 or 2,000 cells per well) and 4,000 T cells per well. Cell killing was assessed by flow cytometry at 48 hours, and the percentage of Nalm6 tumor cell killing was calculated by taking 1 − (no. of Nalm6 cells alive in experimental condition / no. of Nalm6 cells alive in no-T-cell conditions). Effector cells were stained with human NGFR-APC (clone ME20.4, BioLegend, 345108), and target cells were stained with human CD19-PE (clone HIB19, BioLegend, 982402), for flow cytometric analysis.

### Generative artificial intelligence

Artificial intelligence language models (ChatGPT and Claude) were used for generating custom Python scripts for data analysis and visualization, assistance with copyediting and infilling preliminary drafts of some sections based on an author-provided outline. All content generated by artificial intelligence was thoroughly reviewed, edited and verified by the authors.

### Reporting summary

Further information on research design is available in the Nature Portfolio Reporting Summary linked to this article.

## Online content

Any methods, additional references, Nature Portfolio reporting summaries, source data, extended data, supplementary information, acknowledgements, peer review information; details of author contributions and competing interests; and statements of data and code availability are available at https://doi.org/10.1038/s41587-025-02895-3.

## Supplementary information

Supplementary Information Supplementary Figs. 1−7.

Reporting Summary

Supplementary Tables 1−8 Supplementary Tables 1−8.

## Data Availability

The NGS dataset is available on the National Center for Biotechnology Information Sequence Read Archive at BioProject PRJNA1172311 (ref. ^85^). Plasmids for human cell expression and IVT of Dn29, hifiDn29, goldDn29, superDn29, Dn29−dCas9, hifiDn29−dCas9, goldDn29−dCas9 and superDn29−dCas9, as well as attP, e-attP and sgRNA plasmids, are available on Addgene.

## Code availability

RNA-seq analysis scripts and parameter settings are available in our GitHub repository (https://github.com/julianaqmartins/bulkRNAseq) and archived at Zenodo (https://doi.org/10.5281/zenodo.17239032)^86^.
