# Supplementary material for: Site-specific DNA insertion into the human genome with engineered recombinases
Source: Nat Biotechnol. 2025 Nov 6;44(11):1880–93. doi: 10.1038/s41587-025-02895-3 (PMC13751307; doi:10.1038/s41587-025-02895-3)
Supplement: Supplementary file 1 [file 41587_2025_2895_MOESM1_ESM.pdf]

nature biotechnology

Supplementary information

https://doi.org/10.1038/s41587-025-02895-3

Site-specific DNA insertion into the human genome with engineered recombinases

In the format provided by the authors and unedited

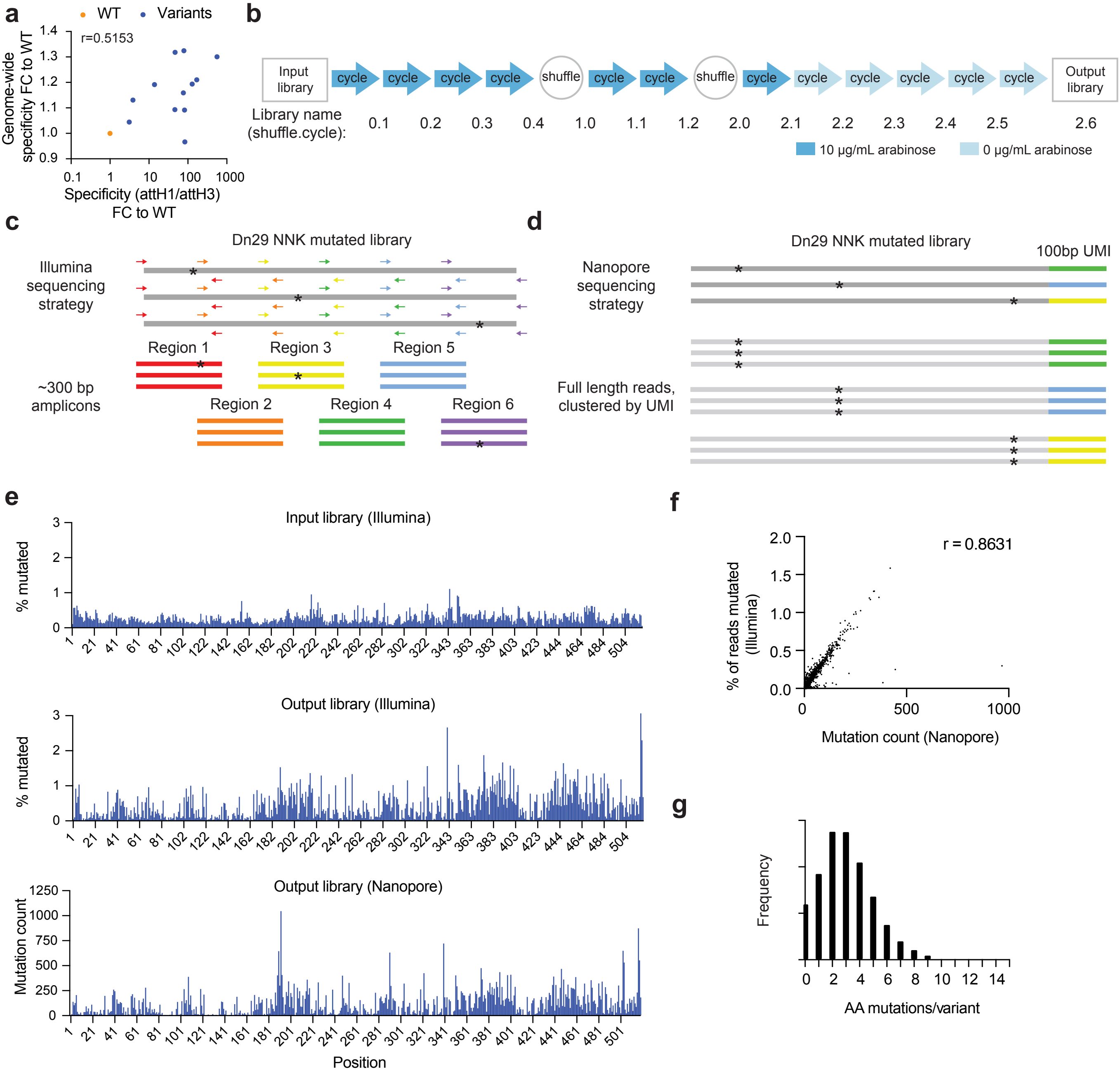

Supplementary Figure 1: High-throughput sequencing and validation of directed evolution library
a, Correlation between genome-wide specificity (ratio of on-target insertions relative to all on- or off-target integration events) and specificity proxy (on-target/single off-target), shown as fold change to WT. Dots represent the mean of *n* = 2 biological replicates. Pearson *r* = 0.5153. **b,** Overview of selection cycles and DNA shuffling steps in directed evolution campaign. **c,** Illumina sequencing strategy for Dn29 NNK library. The 1557 bp CDS is sequenced in PCR fragments, generating 6 regions <300 bp each. **d,** Nanopore sequencing strategy for Dn29 NNK library. Library is cloned into N x 100 bp UMI plasmid backbone, and reads are clustered by UMI for consensus sequence generation. **e,** Percentage of mutated residues at each Dn29 coding sequence position: input library (Illumina) vs output library (Illumina and Nanopore). **f,** Correlation of mutational frequency in output library: Illumina vs Nanopore. Each dot represents a coding sequence position. Pearson *r* = 0.8631, two-tailed *P*<0.0001. **g,** Distribution of amino acid mutations per variant in the output library (Nanopore).

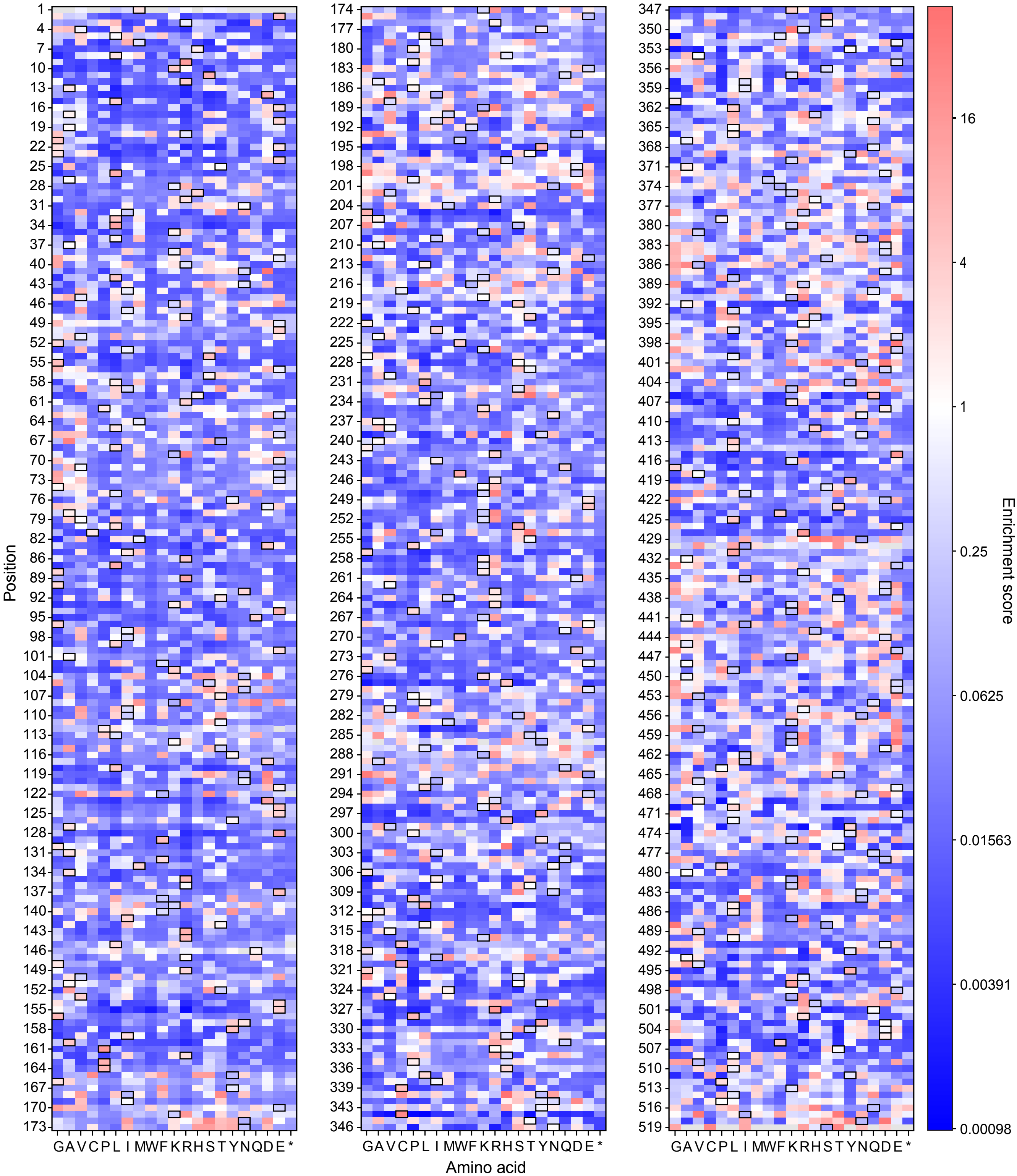

Supplementary Figure 2: Enrichment score of output library normalized to input library. Boxed cells indicate WT amino acids, red and blue cells indicate enrichment or depletion, respectively, white cells indicate enrichment score of 1 (no enrichment), and gray cells represent an amino acid dropout in the input library.

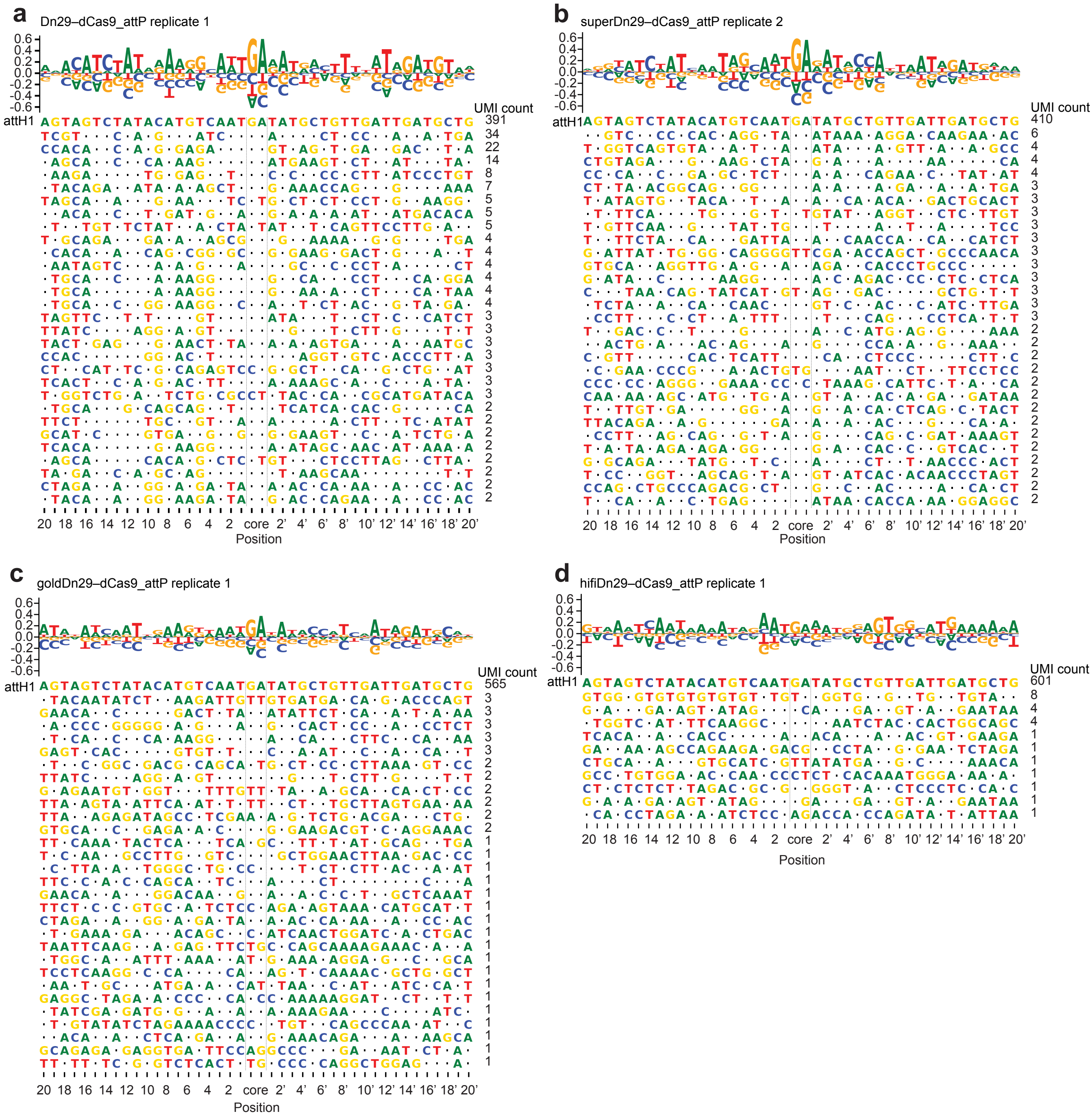

Supplementary Figure 3. Alignment of the top 30 genomic integration sites for (**a**) Dn29–dCas9, (**b**) superDn29–dCas9, (**c**) goldDn29–dCas9, and (**d**) hifiDn29–dCas9. Top panel shows sequence logo derived from all integration sites. The first row below each logo shows the attH1 sequence (sgRNA-targeted pseudosite). Individual integration sites are listed below with their corresponding UMI counts on the right. Each letter represents a position with mismatches to attH1, and dots represent matches. Data show a representative replicate.

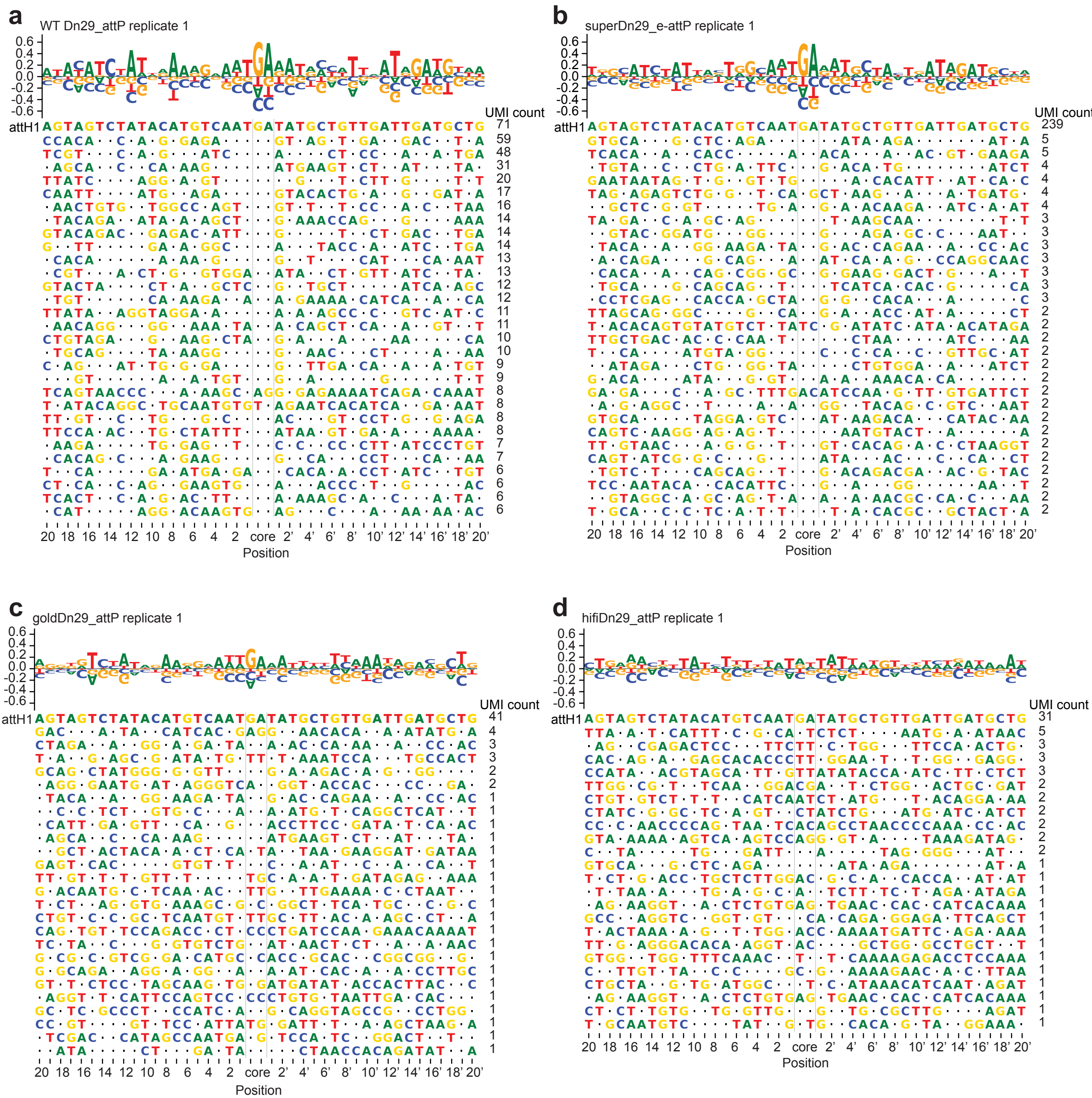

Supplementary Figure 4. Alignment of the top 30 genomic integration sites for (**a**) Dn29, (**b**) superDn29, (**c**) goldDn29, and (**d**) hifiDn29. Top panel shows sequence logo derived from all integration sites. The first row below each logo shows the attH1 sequence. Individual integration sites are listed below with their corresponding UMI counts on the right. Each letter represents a position with mismatches to attH1, and dots represent matches. Data show a representative replicate.

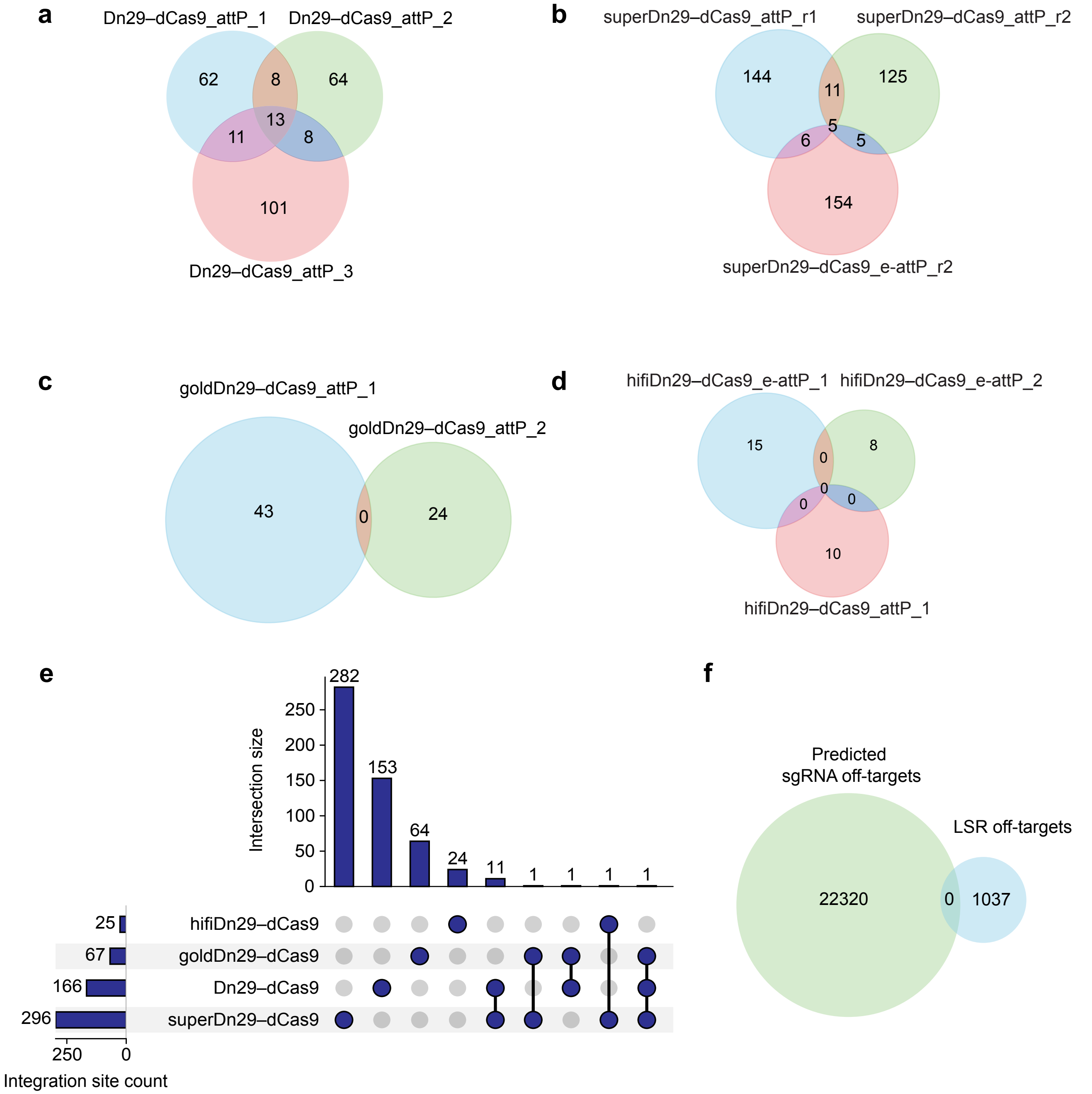

Supplementary Figure 5: Comparison of off-target sites across LSR–dCas9 replicates and variants.
a-d, Venn diagrams of off-target integration sites shared between biological replicates of (**a**) Dn29–dCas9, (**b**) superDn29–dCas9, (**c**) goldDn29–dCas9 and (**d**) hifiDn29–dCas9. **e,** Comparison of off-target integration sites shared between LSR-dCas9 variants. Each row represents all integration sites identified in two biological replicates per variant. **f,** Venn diagram showing overlap between LSR off-target sites and predicted H1-g3 sgRNA off-target sites. Analysis includes LSR integration sites from all replicates and variants that fall within 500 bp of sgRNA off-targets predicted by Cas-OFFinder[1] with up to 5 mismatches and up to 2 nucleotide bulges in either DNA or RNA sequences.

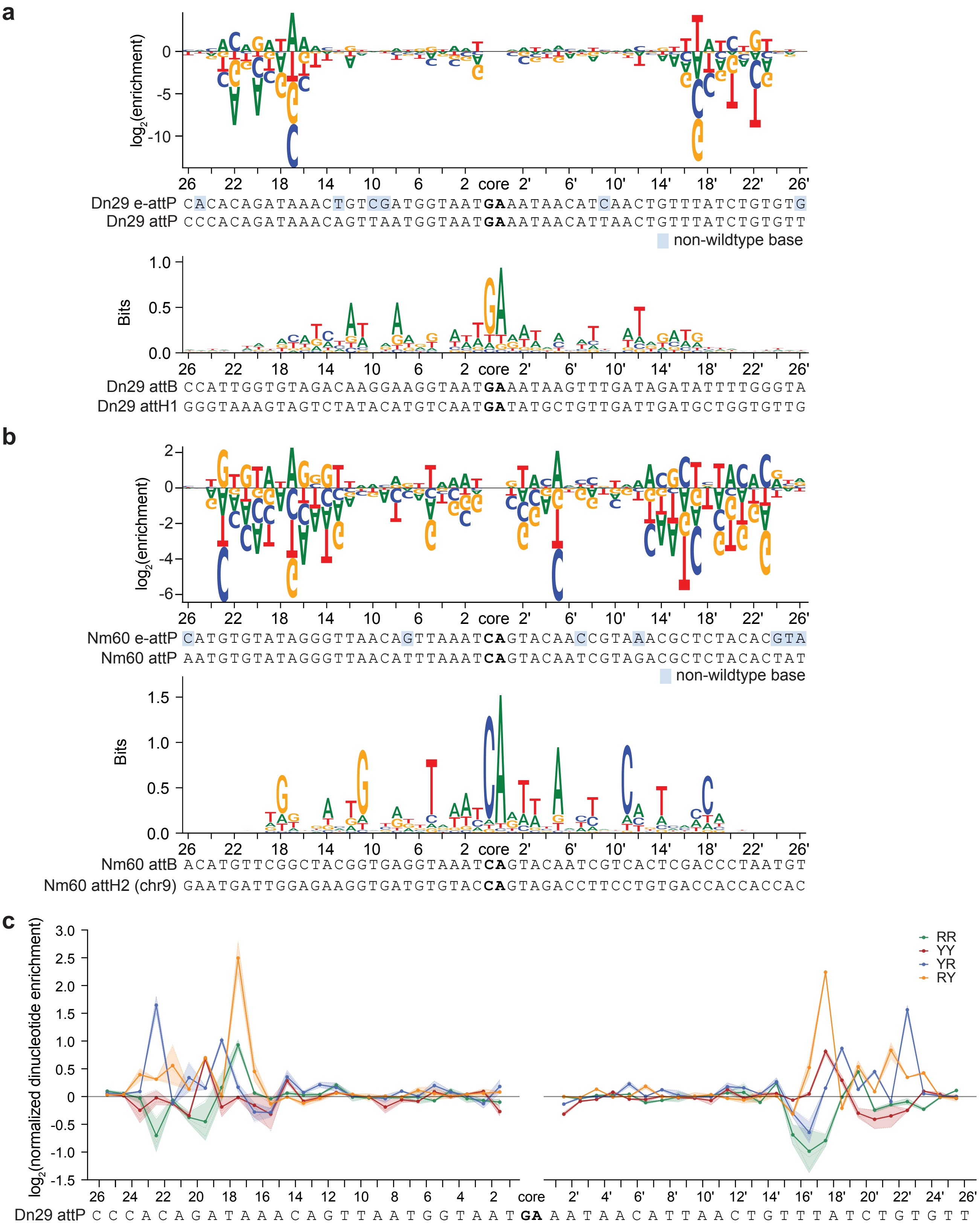
a
log2(enrichment)
0
-5
-10
26 22 18 14 10 6 2 core 2' 6' 10' 14' 18' 22' 26'
Dn29 e-attP CACACAGATAAACTGTCGATGGTAATGAAATAACATCAACTGTTTATCTGTGTG
Dn29 attP CCCACAGATAAACAGTTAATGGTAATGAAATAACATTAACTGTTTATCTGTGTT
non-wildtype base
Bits
1.0
0.5
0.0
Dn29 attB CCATTGGTGTAGACAAGGAAGGTAATGAAATAAGTTTGATAGATATTTTGGGTA
Dn29 attH1 GGGTAAAGTAGTCTATACATGTCAATGATATGCTGTTGATTGATGCTGGTGTTG
b
log2(enrichment)
2
0
-2
-4
-6
Nm60 e-attP CATGTGTATAGGGTTAACAGTTAAATCAGTACAACCGTAAACGCTCTACACGTA
Nm60 attP AATGTGTATAGGGTTAACATTTAAATCAGTACAATCGTAGACGCTCTACACTAT
non-wildtype base
Bits
1.5
1.0
0.5
0.0
Nm60 attB ACATGTTCGGCTACGGTGAGGTAAATCAGTACAATCGTCACTCGACCCTAATGT
Nm60 attH2 (chr9) GAATGATTGGAGAAGGTGATGTGTACCAGTAGACCTTCCTGTGACCACCACCAC
c
log2(normalized dinucleotide enrichment)
3.0 2.5 2.0 1.5 1.0 0.5 0 -0.5 -1.0 -1.5
RR
YY
YR
RY
26 24 22 20 18 16 14 12 10 8 6 4 2 core 2' 4' 6' 8' 10' 12' 14' 16' 18' 20' 22' 24' 26'
Dn29 attP C C C A C A G A T A A A C A G T T A A T G G T A A T GA A A T A A C A T T A A C T G T T T A T C T G T G T T

Supplementary Figure 6: Substrate discrimination and dinucleotide preferences in attP recognition
a,b, Sequence logos showing attP and attB preferences for (**a**) Dn29 and (**b**) Nm60. Top panels show nucleotides enriched and depleted in attP from the substrate screen (average of two biological replicates), along with attP and e-attP sequences. Bottom panels show attB consensus motifs derived from genome-wide integration sites (n = 381 Dn29–dCas9 integration sites and n = 269 Nm60–dCas9 integration sites), along with attB and the sgRNA targeted attH sequences. **c,** Dinucleotide enrichment analysis of integrant library sequences using a 2-bp sliding window, with observed dinucleotide frequencies normalized to expected frequencies of adjacent single nucleotides. Dashed lines represent the mean of each dinucleotide across two biological replicates. Solid lines represent the mean of the dinucleotide category (RR= purine-purine, RY=purine-pyrimidine, YR= pyrimidine-purine, YY=pyrimidine-pyrimidine). Shading represents ± 1 s.d.

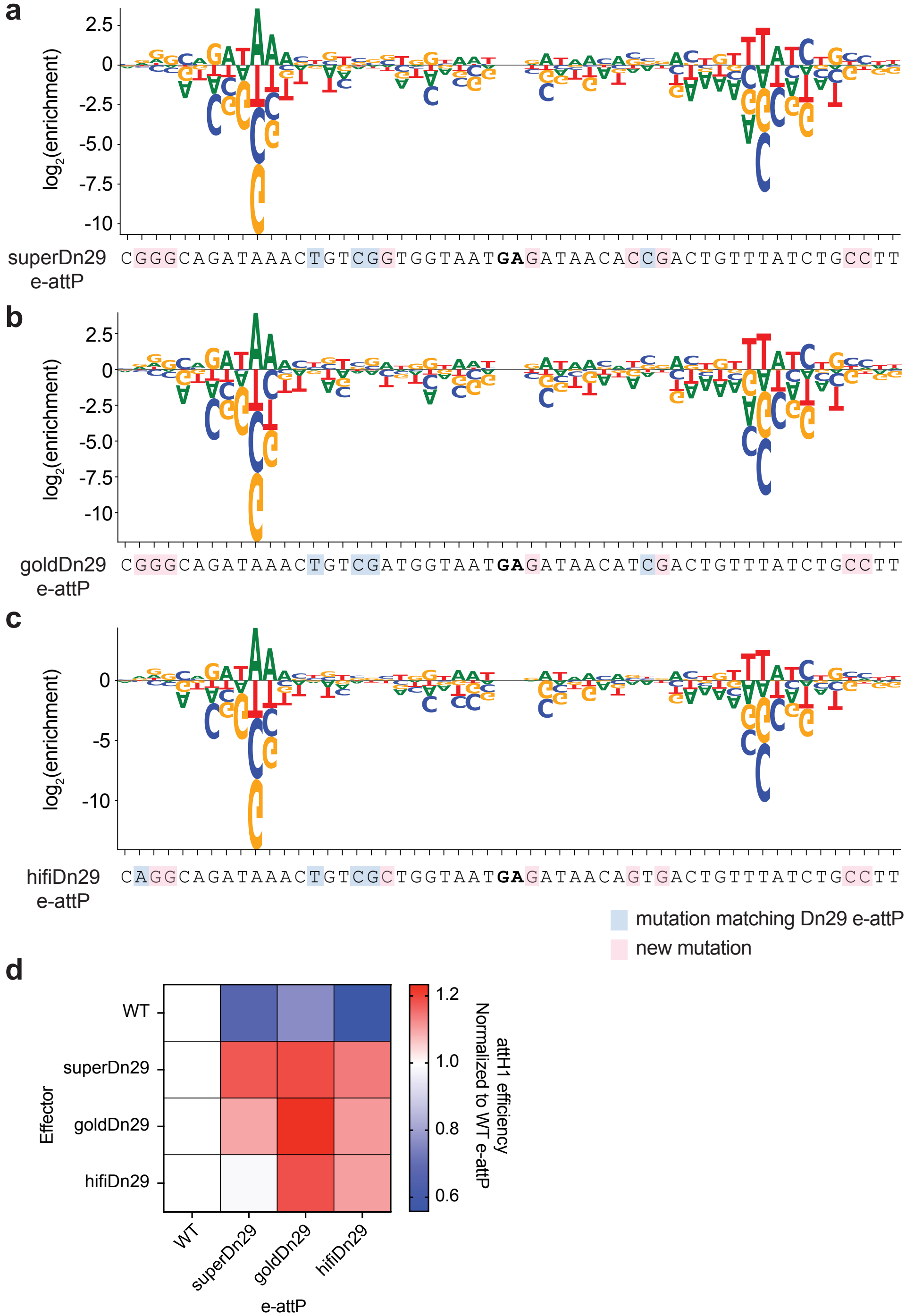

Supplementary Figure 7: Variant-specific e-attP sequence optimization

a-c, Sequence logos showing nucleotide enrichment and depletion in attP substrates for (**a**) superDn29, (**b**) goldDn29, and (**c**) hifiDn29 variants. Blue positions indicate mutations in the variant e-attP that match the WT e-attP sequence. Red positions indicate mutations unique to the variant compared to WT e-attP. Data represent the mean of n = 2 biological replicates. **d,** Heatmap showing integration efficiency at attH1 for all variants paired with all variant e-attPs, normalized to integration efficiency with wildtype e-attP. Data represent mean of n = 3 biological replicates.

References

1. Bae, S., Park, J. & Kim, J. S. Cas-OFFinder: a fast and versatile algorithm that searches for potential off-target sites of Cas9RNA-guided endonucleases. Bioinformatics 30, 1473−1475 (2014).
