# Supplementary material for: Site-specific DNA insertion into the human genome with engineered recombinases
Source: Nat Biotechnol. 2025 Nov 6;44(11):1880–93. doi: 10.1038/s41587-025-02895-3 (PMC13751307; doi:10.1038/s41587-025-02895-3)
Supplement: Supplementary file 2 [file 41587_2025_2895_MOESM2_ESM.pdf]

nature portfolio

Corresponding author(s): Patrick Hsu

Last updated by author(s): 10/02/2025

Reporting Summary

Nature Portfolio wishes to improve the reproducibility of the work that we publish. This form provides structure for consistency and transparency in reporting. For further information on Nature Portfolio policies, see our Editorial Policies and the Editorial Policy Checklist.

Statistics

For all statistical analyses, confirm that the following items are present in the figure legend, table legend, main text, or Methods section.

n/a	Confirmed	
☐	☒	The exact sample size (*n*) for each experimental group/condition, given as a discrete number and unit of measurement
☐	☒	A statement on whether measurements were taken from distinct samples or whether the same sample was measured repeatedly
☐	☒	The statistical test(s) used AND whether they are one- or two-sided<br>*Only common tests should be described solely by name; describe more complex techniques in the Methods section.*
☒	☐	A description of all covariates tested
☐	☒	A description of any assumptions or corrections, such as tests of normality and adjustment for multiple comparisons
☐	☒	A full description of the statistical parameters including central tendency (e.g. means) or other basic estimates (e.g. regression coefficient) AND variation (e.g. standard deviation) or associated estimates of uncertainty (e.g. confidence intervals)
☐	☒	For null hypothesis testing, the test statistic (e.g. *F*, *t*, *r*) with confidence intervals, effect sizes, degrees of freedom and *P* value noted<br>*Give P values as exact values whenever suitable.*
☒	☐	For Bayesian analysis, information on the choice of priors and Markov chain Monte Carlo settings
☒	☐	For hierarchical and complex designs, identification of the appropriate level for tests and full reporting of outcomes
☐	☒	Estimates of effect sizes (e.g. Cohen's *d*, Pearson's *r*), indicating how they were calculated

Our web collection on statistics for biologists contains articles on many of the points above.

Software and code

Policy information about availability of computer code

Data collection	Next generation sequencing was collected using the following software: NextSeq 1000/2000 Control Software Suite v1.7.1, Miseq Control Software v4.1.0, MinKNOW UI v6.5.15, and Illumina Basespace v7.38.0. Flow cytometry was collecting using Attune Cytometric Software v5.3.0. ddPCR was collected using the Bio-Rad QX Manager Software (version 2.1.0). qPCR was collected using the LightCycler 480 Software (v1.5.1.62).
Data analysis	Custom python scripts were used to analyze NGS data as described in Methods, using nanoq (v.0.9.0), cutadapt (v.1.18), mmseqs easy-linclust (v.14.7e284), Medaka (v.1.9.1), BBmerge (v39.06), Bio (v1.73), seaborn (v0.11.2),Trim Galore (v0.6.7), STAR (v2.7.10a), Picard (v2.27.4), RSeQC (v4.0.0), Qualimap (v2.2.2), dupRadar (v3.21), Salmon (v1.10.0), nf-core/rnaseq pipeline (v3.12.0), Nextflow (v24.10.0), Docker (v28), R (v4.3.1), DESeq2 (v1.38.1), BWA (v0.7.19), bedtools (v2.31.0), and Samtools (v1.22). NGS quality scores were visualized with FastQC (v0.12.0) and (v0.11.9) and MultiQC (v1.15). Flow cytometry was analyzed using FlowJo v10.10.0. Data visualization was performed using python and GraphPad Prism Version 10.3.0. Crystal and alphafold structures were visualized using Pymol Version 3.0.2. ddPCR was analyzed using the Bio-Rad QX Manager Software (version 2.1.0). Numerical data was analyzed using Microsoft Excel version 16.89.1. Sequencing data was analyzed using Geneious Prime (version 11.0.20.1+1). Machine learning was performed and analyzed using standard python packages including scikit-learn (v1.0.2), pandas (v1.3.5), numpy (v1.19.5), matplotlib (v3.5.2), xgboost (v1.6.2), scipy (v1.7.3), catboost (v1.2.5), with all parameters reported in the methods.

For manuscripts utilizing custom algorithms or software that are central to the research but not yet described in published literature, software must be made available to editors and reviewers. We strongly encourage code deposition in a community repository (e.g. GitHub). See the Nature Portfolio guidelines for submitting code & software for further information.

Data

Policy information about availability of data

All manuscripts must include a data availability statement. This statement should provide the following information, where applicable:

- Accession codes, unique identifiers, or web links for publicly available datasets
- A description of any restrictions on data availability
- For clinical datasets or third party data, please ensure that the statement adheres to our policy

The NGS dataset is available on the NCBI Sequence Read Archive at Bioproject PRJNA1172311. Plasmids for human cell expression and in vitro transcription of Dn29, hifiDn29, goldDn29, superDn29, Dn29-dCas9, hifiDn29-dCas9, goldDn29-dCas9, and superDn29-dCas9, as well as attP, e-attP, and sgRNA plasmids are available on Addgene.

Research involving human participants, their data, or biological material

Policy information about studies with human participants or human data. See also policy information about sex, gender (identity/presentation), and sexual orientation and race, ethnicity and racism.

Reporting on sex and gender	*Use the terms sex (biological attribute) and gender (shaped by social and cultural circumstances) carefully in order to avoid confusing both terms. Indicate if findings apply to only one sex or gender; describe whether sex and gender were considered in study design; whether sex and/or gender was determined based on self-reporting or assigned and methods used. Provide in the source data disaggregated sex and gender data, where this information has been collected, and if consent has been obtained for sharing of individual-level data; provide overall numbers in this Reporting Summary. Please state if this information has not been collected. Report sex- and gender-based analyses where performed, justify reasons for lack of sex- and gender-based analysis.*
Reporting on race, ethnicity, or other socially relevant groupings	*Please specify the socially constructed or socially relevant categorization variable(s) used in your manuscript and explain why they were used. Please note that such variables should not be used as proxies for other socially constructed/relevant variables (for example, race or ethnicity should not be used as a proxy for socioeconomic status). Provide clear definitions of the relevant terms used, how they were provided (by the participants/respondents, the researchers, or third parties), and the method(s) used to classify people into the different categories (e.g. self-report, census or administrative data, social media data, etc.) Please provide details about how you controlled for confounding variables in your analyses.*
Population characteristics	*Describe the covariate-relevant population characteristics of the human research participants (e.g. age, genotypic information, past and current diagnosis and treatment categories). If you filled out the behavioural & social sciences study design questions and have nothing to add here, write "See above."*
Recruitment	*Describe how participants were recruited. Outline any potential self-selection bias or other biases that may be present and how these are likely to impact results.*
Ethics oversight	*Identify the organization(s) that approved the study protocol.*

Note that full information on the approval of the study protocol must also be provided in the manuscript.

Field-specific reporting

Please select the one below that is the best fit for your research. If you are not sure, read the appropriate sections before making your selection.

☒ Life sciences ☐ Behavioural & social sciences ☐ Ecological, evolutionary & environmental sciences

For a reference copy of the document with all sections, see nature.com/documents/nr-reporting-summary-flat.pdf

Life sciences study design

All studies must disclose on these points even when the disclosure is negative.

Sample size	Sample sizes chosen according to or exceeding standards in the field.
Data exclusions	For the computational models of higher-order mutation combinations, 2 higher order mutants containing the mutation N214Y were removed from the test set because this mutation was a dropout in the training set, so the model was not valid for this mutation. 11 variants that contained 0 measurable off-target integrations into attH3 by ddPCR were removed from the test set, because the specificity was not calculable (divide by 0 error).
Replication	Each experiment was performed with biological replicates, at a minimum 2-3, at the standard for the field. For qPCR, three technical replicates were performed per biological replicate.
Randomization	Covariates were controlled through the following approaches: (1) All measurements were normalized to wild-type controls included in each independent experiment to account for inter-experimental variation in transfection efficiency, cell health, and reagent batch effects. (2) Edge

wells were systematically avoided in all key transfections to eliminate position-dependent effects. (3) Samples were randomized across plate positions within each experiment to prevent confounding of biological conditions with spatial variation.

Blinding: Investigators were blinded for ddPCR gating analyses.

Reporting for specific materials, systems and methods

We require information from authors about some types of materials, experimental systems and methods used in many studies. Here, indicate whether each material, system or method listed is relevant to your study. If you are not sure if a list item applies to your research, read the appropriate section before selecting a response.

Materials & experimental systems

n/a	Involved in the study	
☐	☒	Antibodies
☐	☒	Eukaryotic cell lines
☒	☐	Palaeontology and archaeology
☒	☐	Animals and other organisms
☒	☐	Clinical data
☒	☐	Dual use research of concern
☒	☐	Plants

Methods

n/a	Involved in the study	
☒	☐	ChIP-seq
☐	☒	Flow cytometry
☒	☐	MRI-based neuroimaging

Antibodies

Antibodies used: APC CD81 (BD, Cat:551112, Lot:2061009, clone JS-81, 1:100 dilution); APC CD147 (ThermoFisher Scientific, Cat:A15706, Lot 540242, clone 8D12, 1:100 dilution); Alexa Fluor 647 CD63 (BD, Cat:561983, Lot:2112938, clone H5C6, 1:100 dilution); APC CD63 (BioLegend, Cat:353008, Lot:B373947, clone H5C6, 1:100 dilution); APC/Cyanine7 CD34 (BioLegend, Cat: 343514, Lot:B413134, clone 581, 1:100 dilution); PE CD43 (BioLegend, Cat:343204, Lot:B359578, clone CD43-10G7, 1:100 dilution). Human NGFR-APC (Biolegend, cat #345108, Clone: ME20.4, lot B450617 1:100 dilution), Human CD19-PE (Biolegend, Cat: 982402, Clone: HIB19, lot B383907, 1:100 dilution). Alexa Fluor 647 conjugated Anti-phospho Histone H2A.X (Ser139) antibody (Sigma-Aldrich, Cat: 05-636-AF647, clone JBW301, Lot 4214083, 1:1000 dilution).

Validation: All antibodies chosen are validated for flow cytometric analysis of human cells according to the manufacturer's website. All flow experiments contained negative controls and non-stained controls.

Eukaryotic cell lines

Policy information about cell lines and Sex and Gender in Research

Cell line source(s): HEK293FT were obtained from Thermo Fisher. (Cat. #R70007) (Female)
Primary human T cells were obtained through STEMCELL Technologies from deidentified healthy donors (Cat #200-0092). (Male or female)
H1 human embryonic stem cells were obtained from WiCell Research Institute (Male)
WTC-11 induced pluripotent stem cells were obtained from Coriell Institute for Medical Research (GM25256) (Male)
Peripheral blood mononuclear cells (PBMCs) from healthy human blood donors were collected under an approved IRB protocol by the Stanford Blood Center

Authentication: None of the cell lines used were authenticated.

Mycoplasma contamination: HEK293FT, WTC11 ,and H1's tested negative for mycoplasma, tested monthly.
Primary human T cells and PBMCs were not testsed for mycoplasma.

Commonly misidentified lines (See ICLAC register): None of the cell lines used were found on the ICLAC database

Plants

Seed stocks	*Report on the source of all seed stocks or other plant material used. If applicable, state the seed stock centre and catalogue number. If plant specimens were collected from the field, describe the collection location, date and sampling procedures.*
Novel plant genotypes	*Describe the methods by which all novel plant genotypes were produced. This includes those generated by transgenic approaches, gene editing, chemical/radiation-based mutagenesis and hybridization. For transgenic lines, describe the transformation method, the number of independent lines analyzed and the generation upon which experiments were performed. For gene-edited lines, describe the editor used, the endogenous sequence targeted for editing, the targeting guide RNA sequence (if applicable) and how the editor was applied.*
Authentication	*Describe any authentication procedures for each seed stock used or novel genotype generated. Describe any experiments used to assess the effect of a mutation and, where applicable, how potential secondary effects (e.g. second site T-DNA insertions, mosiacism, off-target gene editing) were examined.*

Flow Cytometry

Plots

Confirm that:

☒ The axis labels state the marker and fluorochrome used (e.g. CD4-FITC).

☒ The axis scales are clearly visible. Include numbers along axes only for bottom left plot of group (a 'group' is an analysis of identical markers).

☒ All plots are contour plots with outliers or pseudocolor plots.

☒ A numerical value for number of cells or percentage (with statistics) is provided.

Methodology

Sample preparation	HEK293FTs - cells are cultured in 96 well format. On the day of harvesting cells for flow, the cells are dissociated using TrypLE for 5 minutes and resuspended in BD Stain Buffer with FBS (554656).<br>T cells - 50 µL of T cells were collected for staining and flow cytometry. Cells were centrifuged, washed once with 200 µl cell BD Stain buffer, and stained with Ghost Dye™ Red 780 at a 1:1000 dilution (Tonbo, Cat #13-0865-T500) for 20 minutes in the dark at 4°C.<br>H1-derived Hematopoietic progenitor cells - 250 µL of non-adherent cells were collected from the supernatant using wide bore P1000 tips and transferred to a V-bottom 96 well plate. Next, the cells were pelleted at 400g for 5 minutes, supernatant discarded, and resuspended in 95 mL Stain Buffer (BD) containing 1 µL of each antibody with a wide bore pipette. The following antibodies were used: APC CD81 (BD, Cat:551112), APC CD147 (Thermo Fisher, Ref: A15706), Alexa Fluor® 647 CD63 (BD, Cat: 561983), APC/Cyanine7 CD34 (BioLegend, Cat: 343514), PE CD43 (BioLegend, Cat: 343204). The cells were incubated in the dark for 20 minutes to 1 hour, and washed once with Stain Buffer.
Instrument	Attune NxT Flow Cytometer with Autosampler (Thermo Fisher)
Software	To collect flow cytometry data, the Attune Cytometric Software v5.3.0 was utilized. Data was analyzed using Flowjo v10.10.0.
Cell population abundance	A minimum of 20,000 cells were analyzed per well.
Gating strategy	Live cells were determined through FSC-A and SSC-A gating, or when relevant, through live-dead staining with Sytox Orange (Thermo Fisher) and Ghost Dye™ Red 780 (Tonbo, Cat #13-0865-T500). Next, FSC-A/FSC-H was utilized to determine singlets. Fluorescent proteins were gated using a non-transfected negative control or a mismatched LSR control for plasmid recombination assays. Antibodies were gated using a non-stained negative control.

☒ Tick this box to confirm that a figure exemplifying the gating strategy is provided in the Supplementary Information.
